# Light whole genome sequence for SNP discovery across domestic cat breeds

**DOI:** 10.1186/1471-2164-11-406

**Published:** 2010-06-24

**Authors:** James C Mullikin, Nancy F Hansen, Lei Shen, Heather Ebling, William F Donahue, Wei Tao, David J Saranga, Adrianne Brand, Marc J Rubenfield, Alice C Young, Pedro Cruz, Carlos Driscoll, Victor David, Samer WK Al-Murrani, Mary F Locniskar, Mitchell S Abrahamsen, Stephen J O'Brien, Douglas R Smith, Jeffrey A Brockman

**Affiliations:** 1 Genome Technology Branch and NIH Intramural Sequencing Center, National Human Genome Research Institute, National Institutes of Health, Bethesda, Maryland 20892, USA; 2 Agencourt Bioscience Corporation, Beverly, Massachusetts 01915, USA; 3 Laboratory of Genomic Diversity, National Cancer Institute, Frederick, Maryland 21702, USA; 4 Hill's Pet Nutrition Inc., PO Box 1658, Topeka, KS 66601, USA

## Abstract

**Background:**

The domestic cat has offered enormous genomic potential in the veterinary description of over 250 hereditary disease models as well as the occurrence of several deadly feline viruses (feline leukemia virus -- FeLV, feline coronavirus -- FECV, feline immunodeficiency virus - FIV) that are homologues to human scourges (cancer, SARS, and AIDS respectively). However, to realize this bio-medical potential, a high density single nucleotide polymorphism (SNP) map is required in order to accomplish disease and phenotype association discovery.

**Description:**

To remedy this, we generated 3,178,297 paired fosmid-end Sanger sequence reads from seven cats, and combined these data with the publicly available 2X cat whole genome sequence. All sequence reads were assembled together to form a 3X whole genome assembly allowing the discovery of over three million SNPs. To reduce potential false positive SNPs due to the low coverage assembly, a low upper-limit was placed on sequence coverage and a high lower-limit on the quality of the discrepant bases at a potential variant site. In all domestic cats of different breeds: female Abyssinian, female American shorthair, male Cornish Rex, female European Burmese, female Persian, female Siamese, a male Ragdoll and a female African wildcat were sequenced lightly. We report a total of 964 k common SNPs suitable for a domestic cat SNP genotyping array and an additional 900 k SNPs detected between African wildcat and domestic cats breeds. An empirical sampling of 94 discovered SNPs were tested in the sequenced cats resulting in a SNP validation rate of 99%.

**Conclusions:**

These data provide a large collection of mapped feline SNPs across the cat genome that will allow for the development of SNP genotyping platforms for mapping feline diseases.

## Background

Along with dogs, the domestic cat enjoys extensive veterinary surveillance, more than any other animal. A rich literature of feline veterinary models reveals a unique opportunity to explore genetic determinants responsible for genetic diseases, infectious disease susceptibility, behavioral and neurological phenotypes, reproduction and physiology (see [1] and [2] for citations). As a highly venerated pet this extraordinarily successful domestic species comprises as many as one billion individuals worldwide. House cats have become a familiar companion to people since their original domestication from the Asian wildcat (*Felis silvestris lybica*), recently estimated at approximately 10,000 years ago in the Middle East's Fertile Crescent[3]. In spite of our affection for cats, advances in clinical resolution of genetic maladies and complex diseases has been slower than for other species largely due to a delay in achieving a useful whole genome sequence of the cat. This has changed recently with the completion of a draft 1.9X genome sequence of a female Abyssinian cat named Cinnamon who gave us our first glimpse and hope of developing the species as an active player in the genomics era[1,4].

The availability of a sufficiently dense single-nucleotide polymorphism (SNP) map for a species provides a resource which enables the power of automated high-throughput genotyping to associate regions of the genome to hereditary diseases, quantitative traits, and other phenotypes. High density SNP maps are available for many species including human, mouse, dog, chicken, and rice (for a complete list see [5]). The cat genome has a moderate collection of SNPs, however the 327,037 available SNPs are clustered into alternating genomic segments of high SNP density and homozygous regions; approximately 60% of Cinnamon's genome is homozygous[4]. This patchwork pattern of the current 1.9X genome sequence was derived from a single inbred cat. To supplement feline SNP map and the genome assembly, we created fosmid libraries and sequenced six additional cats of different breeds and one African wild cat. These sequences dramatically improve the SNP map by increasing the total number of useful SNPs and by filling in the long stretches of genomic homozygosity (~60% of the genome) reported in the 1.9X genome sequence of Cinnamon[4]. In order to make the best use of the additional sequence reads for SNP discovery, we generated a new assembly; the new reads increase the depth-of-coverage of the genome by 50%. This translates to 25% more genomic sequence. Thus this resource provides an improved cat genome assembly as well as a greatly improved SNP map (Assembly: NCBI Accession ACBE00000000, NCBI dbSNP handle CAT_POLY_V17E, and [6]).

## Construction and content

### Samples and sequence generation

DNA from six domestic cats and one wildcat was collected and isolated using the PAXgene Blood DNA kit as per manufactures instructions (Qiagen, Inc., Valencia, CA.) and stored at -80°C until library construction. The domestic cat samples represented six different breeds from pet owners in the Topeka, KS area, and the wildcat sample was from a captive animal residing at the Audubon Nature Institute, LA. Fosmid libraries were prepared in vector pCC2fos from each DNA sample with an average insert size of approximately 37 kb. A total of 3,178,297 paired-end sequencing reads were generated from the seven libraries (Table 1) using standard methods with SPRI-based DNA purification and Big Dye terminator sequencing reagents on ABI3730xl instruments.

**Table 1:** Total sequencing reads generated, SNP counts, SNP rate, and gender

Cat Name	Cat	Reads	SNPs	SNP rate (per *x *bases)	Gender
**Pixel**	**Burmese**	**331,813**	**174,212**	**524**	♀
**Zeelie**	**Persian**	**298,332**	**174,706**	**510**	♀
**Tipper**	**Cornish Rex**	**272,607**	**164,054**	**503**	♂
**Scooter**	**Ragdoll**	**298,409**	**168,455**	**510**	♂
**Speedy**	**Domestic Shorthair**	**310,364**	**158,148**	**569**	♀
**Cocoa**	**Siamese**	**293,712**	**152,984**	**516**	♀
**Nancy**	**African wild cat**	**1,373,060**	**938,386**	**360**	♀
Cinnamon*	**Abyssinian**	**8,186,934**	**1,323,794**	**1520**	♀

* Cinnamon is listed but those reads were not part of the sequence generated from this effort.

### Reads, assembly and mapping

Assembly of the cat genome was carried out in a similar method as published for the 1.9X assembly of Cinnamon[4], except in this case the Phusion method[7] was used for contig and scaffold generation. All 11.4M reads (Table 1) were used, comprising approximately 2.8-fold read redundancy. Contig N50 size is 4.6 kb, an increase of nearly 100% from 2.4 kb in the 1.9X assembly, with total assembled bases at 2.0 Gb, an increase from 1.64 Gb in the 1.9X assembly by 22% (see Additional file 1 Table S1 for a complete listing of the assembly statistics). Mapping of the assembly onto chromosomes used a similar method as previously described[4] with the total sequence placed onto cat chromosomes at 1.71 Gb, an increase from 1.36 Gb. Comparison of this new assembly and the 1.9X assembly to the highly collated NIH Intramural Sequencing Center (NISC) generated sequence across feline ENCODE regions[8] shows substantial improvement in coverage from 72% to 84% (Additional file 2 Table S2), as well as excellent order and orientation (Additional file 3 and Additional file 4). The 84% coverage figure is a sampling estimate derived from assessing the exact coverage of 26.6 Mb of BAC clone sequence from ENCODE target regions to this assembly, resulting in a higher level of coverage across these regions than an estimated genome average. For example the estimated euchromatic genome size is 2.5 Gb; thus the overall assembled base-pair coverage would be closer to 2.0/2.5 = 80%. The ~4% discrepancy could be due to ENCODE's selection of relatively gene-rich and more highly evolutionarily conserved regions which are likely easier to assemble. In fact, the two lowest covered regions, ENr112 and ENr113, at about 47% coverage, are ENCODE regions with no genes and very low multi-species conservation.

### SNPs and DIPs

SNPs are called using the ssahaSNP method[9] by comparing the sequence of each read relative to the consensus sequence assembly, with a breakdown of total SNPs discovered per cat provided in Table 1. Some fraction of these SNPs is discovered in more than one cat, thus the non-redundant total, 3,077,846, is less than the totals across all the cats, 3,254,739. The SNP rate is calculated relative to the reference sequence, and is determined as the number of neighborhood quality standard (NQS) bases divided by the SNPs detected from a given cat's sequence traces. The NQS method[10] uses a set of parameters to decrease the probability of a base being called incorrectly, which in turn lowers the false reporting of discrepant bases. The settings of the NQS parameters used here are as follows: the base has a PHRED[11,12] quality score of > = 23, the five bases to either side have a PHRED quality score > = 15, and nine of these ten flanking bases are perfect matches. Since the assembly is made up of all reads from all cats, the SNP rates should be viewed in a relative sense, from Cinnamon with the lowest SNP rate at one SNP per 1520 NQS bases to Nancy with the highest rate at one SNP per 360 NQS bases. The SNP rate is much lower for Cinnamon for two reasons. First, Cinnamon dominates the assembly with the most reads, thus comparing a sequence read from Cinnamon to the assembly has an increased chance of comparing to the same haploid. Second, Cinnamon is inbred and her genome is 60% homozygous, further increasing the chance of comparing a read to the same haploid. At the other extreme, the wild cat, Nancy, gives the highest SNP rate since the African wildcat, *Felis silvestris cafra*, is one of several continental subspecies of wildcat, the parent species for cat domestication. Domestic cat breeds descend from a founder event (domestication itself) which reduced genomic diversity appreciably relative to the wildcat species[3]. Domestic cat breeds displayed a SNP rate of 1 SNP/5-600 bp. In the 1.9X cat assembly, the rate of polymorphisms is estimated at one SNP per 600 bases within the heterozygous segments, thus the estimates for the other breeds agree quite well with this previous measure. In addition to SNPs, 682,085 deletion and insertion polymorphisms (DIPs) are detected.

### Sampling SNP variation empirically

To validate the accuracy of SNP detection we randomly chose 555 SNPs detected from the fosmid-end sequence of six domestic cats sequenced in this study. We selected SNPs located at least 750 bases away from a sequence contig gap (to make primer design feasible) reducing the number of testable SNPs to 393 of which 348 yielded primers using a primer design package[13]. Two sets of PCR primer plates (47 each) passed stringent primer QC and were sequenced across the six cats plus one additional unrelated domestic shorthair.

Sequence traces were analyzed using PolyPhred version 6.11[14-16] and the targeted variant bases were viewed using Consed[17]. Of 94 variants, 92 were confirmed, one had low quality sequence traces for the cat carrying the detected variant (Nancy), and one variant detected from the ragdoll, Scooter, was not observed in this cat. Removing the single low-quality amplimer gives an overall validation rate at 92/93 = 99%. Additional file 5 Table S3 gives a complete listing of all SNPs and genotypes from this validation experiment.

Forty-five detected DIPs from the sequence traces fell within the PCR amplicons. Of these, 43 were empirically validated, one was not tested and one did not validate (a single base insertion). Therefore, the DIP validation rate would be 43/45 = 96%.

## PCR re-sequencing

### gDNA QC

gDNA concentration was determined using a DyNA Quant 200 fluorometer (Hoefer) and the dsDNA specific dye Hoechst Dye 22358 according to the manufacturer's protocol. The gDNA sample was then tested for functionality in PCR reactions with positive and negative control primers.

Pos_For: TGTAAAACGACGGCCAGTATCCCACTGTTAGGAGAACTGC

Pos_Rev: CAGGAAACAGCTATGACCGGTCAGGAAAGGGACACAGATA

Negative control primers were the forward and reverse sequencing primers to lac-Z of M13.

M13_For: TGTAAAACGACGGCCAGT

M13_Rev: CAGGAAACAGCTATGACC

To each gDNA a trace amount of a plasmid with a unique non-feline insert was added. This plasmid was used as a biological barcode. The identifying inserts were amplified and checked using the universal sequencing primers above. The gDNAs were then diluted to a working concentration of 2.5 ng/ul.

### Primer QC and Sequencing

Primers were obtained from Eurofins MWG Operon in individual tubes and reconstituted to 100 uM in 10 mM TRIS, pH 8.0, 0.1 mM EDTA. The primer pairs were tested at a concentration of 0.16 uM each in 10 ul PCR reactions containing iQ supermix (BioRad) and 5 ng of control feline DNA (Tipper sample used for first round and Speedy used for second round of QC). Cycling conditions were: activate enzyme at 95C for 3 min, followed by 40 cycles of 95C for 15 sec, 60C for 15 sec, 72C for 60 sec, then 72C for 5 min and hold at 10C. A 5 ul aliquot of the PCR reaction was examined by agarose gel to look for multiple or missing bands. The PCR products were then diluted to 0.4 ng/ul and sequenced in 6 ul reactions using M13 Universal forward and reverse primers and BDT version 3.1 (Applied Biosystems) using standard ABI protocols. The reactions were sequenced on 3730 DNA Sequencers (Applied Biosystems). The sequence traces were then individually inspected for quality. Primer pairs that resulted in high-quality traces were passed. Primers not passing this round were retested using one additional control DNA. Primers failing both rounds were not used.

### PCR

PCR amplification of amplimers was performed in 10 ul reactions in 384-well plates. The reaction conditions were as described above.

## Utility and Discussion

### Distribution

**Figure 1:**
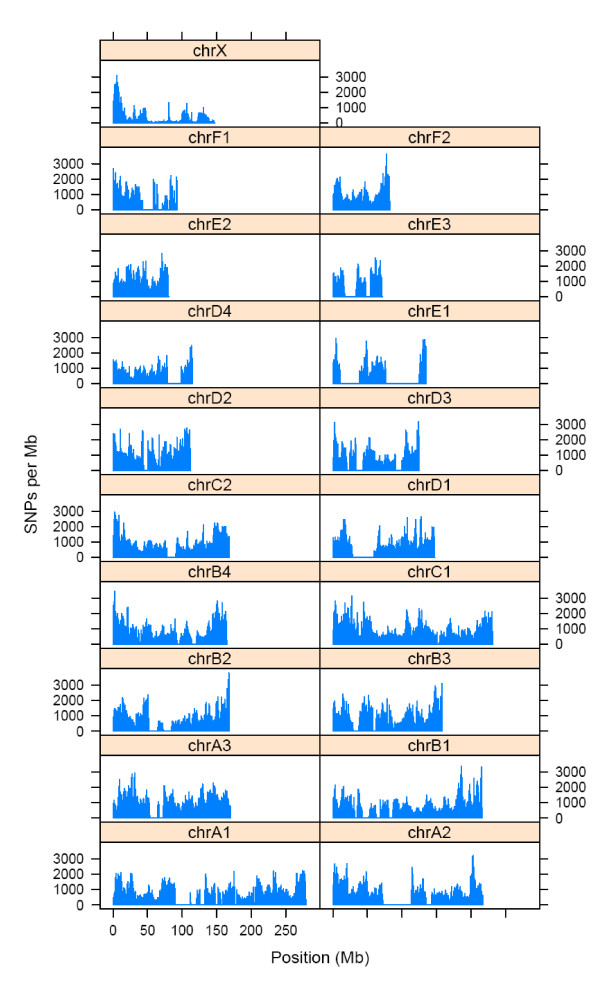
**SNP density in 1 Mb windows**. Number of SNPs in windows of 1 Mb across each chromosome. Zero values are regions that do not have mapped sequence, totaling about 416 Mb, thus the densities are undetermined.

The existing map of 327 k SNPs from the 1.9X Cinnamon assembly includes large homozygous segments covering approximately 60% of the cat genome (see Figure 5 in [4]). Figure 1 shows the incidence of SNPs along cat chromosomes, where the number of SNPs is totaled within adjacent 1 Mb windows across each chromosome. In the new assembly, there are only 8 windows with less than 100 SNPs per Mb across the autosomes (out of 2,569 windows, not counting the unmapped regions). On chromosome X there are 34 windows with less than 100 SNPs out of 143 windows, which suggests that X has relatively lower heterozygosity due to at least three factors: 1) two of the eight cats are male, thus reducing the number of X chromosomes to fourteen instead of sixteen chromosomes for an autosomal locus 2) the effective population size for the X chromosome is ¾ that of the autosomes, and 3) male hemizygosity allows much stronger purifying selection to occur around X-linked functional loci.

**Figure 2:**
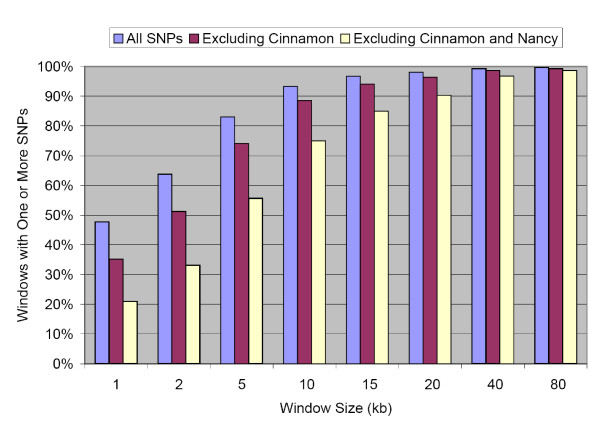
**SNP distribution**. The fraction of windows with one or more SNPs for a range of window sizes and three categories of SNPs: all SNPs, all except Cinnamon and all except Cinnamon and Nancy.

Figure 2 presents the SNP distribution as a fraction of windows of a given size that has at least one SNP. Average SNP spacing in base-pairs is shown in Table 2. For both SNP counts and the average base-pair distance between SNPs there are three SNP categories: **A**) SNPs excluding those discovered only from Cinnamon and/or Nancy, **B**) SNPs excluding those only discovered in Cinnamon, and **C**) all SNPs (Table 2). The reason to count SNPs in these categories is that for some purposes, SNPs derived from the wildcat Nancy are not as useful as SNPs derived from the domesticated cat breeds. Likewise, inclusion of the highly variable densities of SNPs discovered from Cinnamon's much deeper sequencing would reflect this cat's particular pattern of homozygous and heterozygous regions. Thus, SNPs most useful for domestic cat association screens would be those in category **A **which totals 964,285 SNPs (Table 2). An additional restriction of just those category **A **SNPs that are mapped to a cat chromosome reduces this count to 844,313. However, relative to the number of mapped bases, we still have a SNP on average spaced every 2000 bases, and even non-chromosomally mapped SNPs will be useful once these segments are mapped in improved future assemblies. Looking again at Figure 2, over 80% of the 15 kb windows across the genome contain at least one category **A **SNP.

**Table 2:** SNP counts and bases per SNP

		SNP Counts			Bases per SNP		
Chromosome	Non-N Bases	A	B	C	A	B	C
chrA1	164,170,763	77,824	151,421	240,266	2,110	1,084	683
chrA2	120,172,290	59,782	118,896	180,685	2,010	1,011	665
chrA3	109,094,838	55,010	110,129	192,266	1,983	991	567
chrB1	131,184,541	62,260	118,189	196,456	2,107	1,110	668
chrB2	101,553,943	49,898	95,152	161,014	2,035	1,067	631
chrB3	96,970,780	47,679	93,489	167,719	2,034	1,037	578
chrB4	108,425,265	53,709	104,123	170,033	2,019	1,041	638
chrC1	160,223,031	76,483	147,928	245,762	2,095	1,083	652
chrC2	107,198,630	53,479	98,226	163,338	2,004	1,091	656
chrD1	81,705,395	45,881	88,125	130,989	1,781	927	624
chrD2	67,243,459	37,877	71,535	134,493	1,775	940	500
chrD3	71,434,721	40,297	79,074	119,894	1,773	903	596
chrD4	67,338,148	34,295	65,034	88,513	1,963	1,035	761
chrE1	44,074,055	24,513	50,193	87,236	1,798	878	505
chrE2	50,431,338	27,836	56,317	94,520	1,812	895	534
chrE3	36,523,145	24,444	47,449	68,476	1,494	770	533
chrF1	45,373,584	24,292	45,507	83,877	1,868	997	541
chrF2	56,475,142	29,011	55,998	93,247	1,947	1,009	606
chrX	83,845,181	19,431	32,619	65,352	4,315	2,570	1,283
chrUnCf	217,904,849	101,325	198,166	323,505	2,151	1,100	674
chrUn	70,839,159	18,959	34,246	70,797	3,736	2,069	1,001

Total	1,992,182,257	964,285	1,861,816	3,078,438	2,066	1,070	647

		**A**	SNPs excluding Cinnamon and Nancy				
		**B**	SNPs excluding Cinnamon				
		**C**	All SNPs				

With the genotype information, we can estimate how many informative SNPs are available for a genotyping study among cat breeds. There are 964,285 SNPs discovered from at least one of the six breeds sequenced (category **A **SNPs in Table 2). A SNP will not be informative unless it has a minor allele frequency (MAF) of at least 5%. However, the MAFs of these SNPs are not known until they have been genotyped. With the limited number of genotypes given in Additional file 5 Table S3, we observe that 24/92 (26%) are only observed in one cat, and therefore could be quite rare variants. However, the other 68/92 (74%) are seen in 2 or more cats, and are therefore quite likely to be polymorphic, and thus informative. This informative fraction increases to 50/57 (88%) for SNPs detected from domestic cat samples. Thus, about 849 k (88% of 964,285) SNPs remain that are likely to have an informative MAF (> = 5%) among cat breeds from this SNP resource.

In anticipation of using these SNPs for a genotyping chip, one would like to select the SNPs at fairly even intervals across the genome. If a SNP is selected every 15 kb from the category **A **SNPs, 80% of the genome can be covered, requiring about 100,000 SNPs in a genotyping array. The remaining 20% can be filled in with either more widely spaced category **A **SNPs, or using additional category **B **or **C **SNPs. Thus perhaps another 20 k genotype assays for the remaining regions would yield a 120 k SNP chip. This is more than double the estimated number proposed for genome-wide association mapping as reported previously[4].

### Wildcat geographical origin

Finally we investigated the geographical origin of the wildcat Nancy. With nearly 1.4 M reads available for this wild cat, this does provide a valuable resource for studying subspecies of *Felis silvestris*. Nancy was identified as wild-caught 15 years ago in the Arabian Peninsula, so she should geographically fall into the African subspecies *Felis silvestris lybica*. A STRUCTURE analysis was completed using a genomic DNA sample from Nancy, and genotypes from 18 of the 36 short tandem repeat (STR) loci were used to resolve genomic distinctions among wildcat subspecies effectively[3]. Nancy showed no evidence of domestic cat introgression but instead clusters with cats from southern Africa rather than the Near East, so she likely descends form the subspecies *Felis silvestris cafra *rather than *F.s. libyca*. For additional details of this analysis, see Additional file 4 and Additional file 6 Figure S1.

## Conclusions

The primary goal of this effort is to expand feline SNP resources, empowering future linkage and association studies to map feline disease phenotypes to genomic loci. The usefulness of such a resource is clearly proven in many species, most notably for the human and canine genome[18-20]. A recent review[1] highlights this need for additional cat SNPs to aid the development of a genotype array chip of 100,000-150,000 SNPs. The SNPs discovered by this effort should allow the design of such a chip derived from the 964,285 available SNPs from the domestic breed cats.

The value of the sequence generated by this effort will become even greater as the publicly funded effort (see *Felis catus *entries in [21]) to generate a high quality draft of Cinnamon's genome is completed, hopefully within the next year. With a high quality draft covering over 90% of the cat genome, even more SNPs can be extracted from the 3M reads from these seven cats, probably 25% more if the assembled sequence increases to 2.5 Gb from the current 2.0 Gb. This resource is further enhanced by having all reads generated from paired-ends of fosmid templates. The insert sizes are all about 40 kb in size with fairly tight distributions of less than 10% coefficient-of-variation. Given an increasingly higher quality assembly of Cinnamon, these paired-end reads could pinpoint structural rearrangements among cat breeds using available methods[22].

## Availability and requirements

All SNPs and DIPs described are freely available through dbSNP [5] and through the web browser interface [6].

## Authors' contributions

JCM drafted the manuscript. SWKA and MFL organized the sample collection. HE, WFD, WT, DJS, AB, and MJR performed the fosmid library construction and sequencing. PC, VD, ACY and NISC performed the PCR primer design and sequencing. VD and CD did the STR typing and analysis to identify the likely origin of the wildcat. LS, NFH and JCM performed the sequence alignment and SNP/DIP detection and validation review. JCM, JAB, SJO, DRS and MSA conceived of the study, and participated in its design and coordination and helped to draft the manuscript. All authors read and approved the final manuscript.

## Supplementary Material

Additional file 1 **Whole genome assembly statistics**. Table S1 comparing the assembly statistics for this assembly and the previously published 1.9X assembly. Click here for file

Additional file 2 **Assembly coverage of ENCODE regions**. Table S2 listing whole genome shotgun assembly coverage statistics relative to high quality cat BAC clone assemblies of ENCODE regions for this assembly and the previously published 1.9X assembly Click here for file

Additional file 3 **Order and orientation across ENCODE regions**. Plots of assembly order and orientation across all 44 ENCODE regions. Click here for file

Additional file 4 **Descriptions of how Additional files 3 and 6 were generated**. Methods for generating plots of order and orientation across ENCODE regions and the STRUCTURE analysis of Nancy. Click here for file

Additional file 5 **PCR validation results for 94 variants**. Table S3 lists the variants by position on the genome assembly, which alleles are expected, and the alleles observed across 8 cats. Pink colored cells indicate the cat(s) from which the alternate allele was discovered in the light whole genome sequence. Click here for file

Additional file 6 **STRUCTURE analysis results**. Figure S1 shows results of STRUCTURE analysis showing Nancy and representative known-origin wildcat individuals. Click here for file
