# Supplementary material for: Light whole genome sequence for SNP discovery across domestic cat breeds
Source: BMC Genomics. 2010 Jun 24;11:406. doi: 10.1186/1471-2164-11-406 (PMC2996934; doi:10.1186/1471-2164-11-406)
Supplement: Additional file 1 — Whole genome assembly statistics. Table S1 comparing the assembly statistics for this assembly and the previously published 1.9X assembly. [file 1471-2164-11-406-S1.DOCX]

Table S1. Whole genome assembly statistics.

|  | This study | 1.9X cat genome |
| --- | --- | --- |
| Estimated Genome Size | 2.7Gb | 2.7Gb |
| Estimated Euchromatic Genome Size | 2.5Gb | 2.5Gb |
| Coverage | 2.8X | 1.9X |
| Total reads | 11,365,231 | 8,186,934 |
| Read in assembly | 10,052,886 | 6,334,156 |
| Unassembled reads | 1,312,345 | 1,852,778 |
| Contig Count | 604,560 | 817,956 |
| Total Contig Bases | 1,990,628,213 | 1,642,698,337 |
| Contig N50 | 4,581 | 2,378 |
| Scaffold Count | 219,283 | 217,790 |
| Scaffold bases including gaps | 3,180,521,163 | 3,937,914,851 |
| Scaffold N50 including gaps | 162,128 | 117,081 |
| RH markers | 1,680 | 1,680 |
| Total mapped bases | 1,706,681,403 | 1,359,395,505 |
| Total unmapped bases | 283,946,810 | 283,302,832 |
| Total unmapped contigs | 107,988 | 154,476 |
