# Supplementary material for: Light whole genome sequence for SNP discovery across domestic cat breeds
Source: BMC Genomics. 2010 Jun 24;11:406. doi: 10.1186/1471-2164-11-406 (PMC2996934; doi:10.1186/1471-2164-11-406)
Supplement: Additional file 2 — Assembly coverage of ENCODE regions. Table S2 listing whole genome shotgun assembly coverage statistics relative to high quality cat BAC clone assemblies of ENCODE regions for this assembly and the previously published 1.9X assembly [file 1471-2164-11-406-S2.DOC]

Table S2. Whole genome shotgun assembly coverage statistics relative to high quality cat BAC clone assemblies of ENCODE regions.

|  |  | 1.9X WGS Assembly | | | 2.8X WGS Assembly | | |
| --- | --- | --- | --- | --- | --- | --- | --- |
| ENCODE Region | NISC Assembled Bases | Bases Covered | Percent Covered | Contigs | Bases Covered | Percent Covered | Contigs |
| ENr112 | 327,931 | 108,141 | 33.0 | 58 | 150,802 | 46.0 | 55 |
| ENr113 | 116,835 | 34,296 | 29.4 | 25 | 56,153 | 48.1 | 24 |
| ENr324 | 396,940 | 246,855 | 62.2 | 107 | 267,757 | 67.5 | 75 |
| ENr222 | 502,898 | 276,013 | 54.9 | 132 | 372,347 | 74.0 | 117 |
| ENr123 | 542,258 | 325,886 | 60.1 | 135 | 411,706 | 75.9 | 110 |
| ENr323 | 516,985 | 329,840 | 63.8 | 109 | 397,817 | 76.9 | 81 |
| ENr313 | 406,862 | 239,197 | 58.8 | 102 | 314,132 | 77.2 | 79 |
| ENm006 | 1,124,083 | 856,192 | 76.2 | 247 | 868,667 | 77.3 | 155 |
| ENr223 | 513,411 | 329,872 | 64.3 | 154 | 402,745 | 78.4 | 110 |
| ENm012 | 1,017,870 | 640,186 | 62.9 | 255 | 804,781 | 79.1 | 182 |
| ENm001 | 1,874,160 | 1,233,733 | 65.8 | 434 | 1,492,001 | 79.6 | 296 |
| ENm013 | 1,035,115 | 678,610 | 65.6 | 263 | 830,965 | 80.3 | 178 |
| ENm014 | 1,163,319 | 772,556 | 66.4 | 274 | 947,633 | 81.5 | 194 |
| ENm002 | 1,037,217 | 728,684 | 70.3 | 191 | 856,714 | 82.6 | 125 |
| ENr221 | 509,406 | 359,802 | 70.6 | 141 | 421,812 | 82.8 | 68 |
| ENm004 | 1,330,890 | 1,029,680 | 77.4 | 260 | 1,110,670 | 83.5 | 152 |
| ENr114 | 479,384 | 323,459 | 67.5 | 175 | 401,177 | 83.7 | 132 |
| ENm003 | 508,123 | 378,725 | 74.5 | 113 | 430,474 | 84.7 | 59 |
| ENr213 | 469,814 | 317,706 | 67.6 | 114 | 399,149 | 85.0 | 72 |
| ENr311 | 475,687 | 335,275 | 70.5 | 98 | 405,354 | 85.2 | 73 |
| ENm008 | 428,390 | 304,423 | 71.1 | 82 | 368,338 | 86.0 | 47 |
| ENm010 | 480,060 | 357,427 | 74.5 | 114 | 416,265 | 86.7 | 68 |
| ENm009 | 1,238,843 | 931,382 | 75.2 | 240 | 1,076,637 | 86.9 | 183 |
| ENr333 | 560,081 | 412,824 | 73.7 | 124 | 488,412 | 87.2 | 88 |
| ENm011 | 419,643 | 312,011 | 74.4 | 100 | 368,898 | 87.9 | 57 |
| ENr312 | 577,332 | 404,725 | 70.1 | 211 | 508,280 | 88.0 | 133 |
| ENr133 | 449,342 | 351,689 | 78.3 | 109 | 397,326 | 88.4 | 67 |
| ENr231 | 498,676 | 379,122 | 76.0 | 176 | 444,224 | 89.1 | 74 |
| ENm005 | 1,457,233 | 1,166,682 | 80.1 | 313 | 1,304,863 | 89.5 | 188 |
| ENm007 | 450,869 | 333,928 | 74.1 | 80 | 407,715 | 90.4 | 54 |
| ENr233 | 435,416 | 323,089 | 74.2 | 144 | 394,585 | 90.6 | 108 |
| ENr332 | 400,691 | 313,205 | 78.2 | 102 | 363,298 | 90.7 | 64 |
| ENr111 | 504,116 | 401,116 | 79.6 | 102 | 457,923 | 90.8 | 57 |
| ENr331 | 502,002 | 395,478 | 78.8 | 103 | 456,732 | 91.0 | 56 |
| ENr321 | 497,482 | 375,425 | 75.5 | 108 | 454,987 | 91.5 | 63 |
| ENr121 | 424,510 | 331,018 | 78.0 | 99 | 389,111 | 91.7 | 57 |
| ENr232 | 436,816 | 372,454 | 85.3 | 96 | 402,957 | 92.2 | 56 |
| ENr322 | 465,814 | 378,128 | 81.2 | 95 | 429,779 | 92.3 | 50 |
| ENr211 | 481,402 | 411,967 | 85.6 | 95 | 447,696 | 93.0 | 55 |
| ENr334 | 451,766 | 374,176 | 82.8 | 99 | 424,025 | 93.9 | 55 |
| ENr212 | 461,545 | 389,007 | 84.3 | 90 | 438,011 | 94.9 | 48 |
| ENr131 | 249,585 | 224,711 | 90.0 | 52 | 238,827 | 95.7 | 19 |
| ENr132 | 374,658 | 339,546 | 90.6 | 54 | 363,792 | 97.1 | 22 |
| Total | 26,595,460 | 19,128,241 | 71.9% | 6,175 | 22,385,537 | 84.2% | 4,006 |
