# Supplementary material for: Light whole genome sequence for SNP discovery across domestic cat breeds
Source: BMC Genomics. 2010 Jun 24;11:406. doi: 10.1186/1471-2164-11-406 (PMC2996934; doi:10.1186/1471-2164-11-406)
Supplement: Additional file 3 — Order and orientation across ENCODE regions. Plots of assembly order and orientation across all 44 ENCODE regions. [file 1471-2164-11-406-S3.PDF]

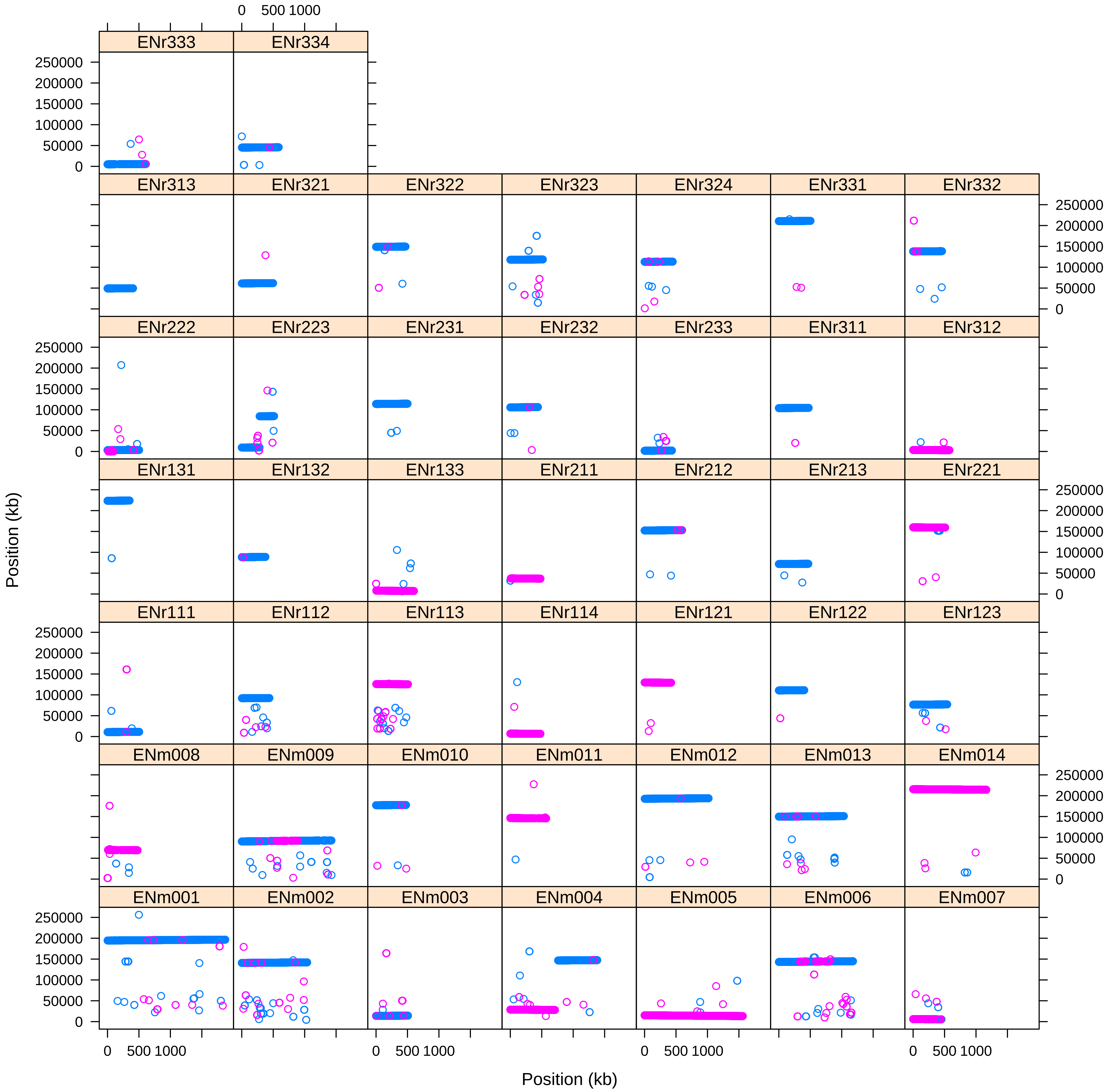

ENr333
ENr334
ENr313
ENr321
ENr322
ENr323
ENr324
ENr331
ENr332
ENr222
ENr223
ENr231
ENr232
ENr233
ENr311
ENr312
ENr131
ENr132
ENr133
ENr211
ENr212
ENr213
ENr221
ENr111
ENr112
ENr113
ENr114
ENr121
ENr122
ENr123
ENm008
ENm009
ENm010
ENm011
ENm012
ENm013
ENm014
ENm001
ENm002
ENm003
ENm004
ENm005
ENm006
ENm007
0
50000
100000
150000
200000
250000
0
500
1000
Position (kb)
Position (kb)

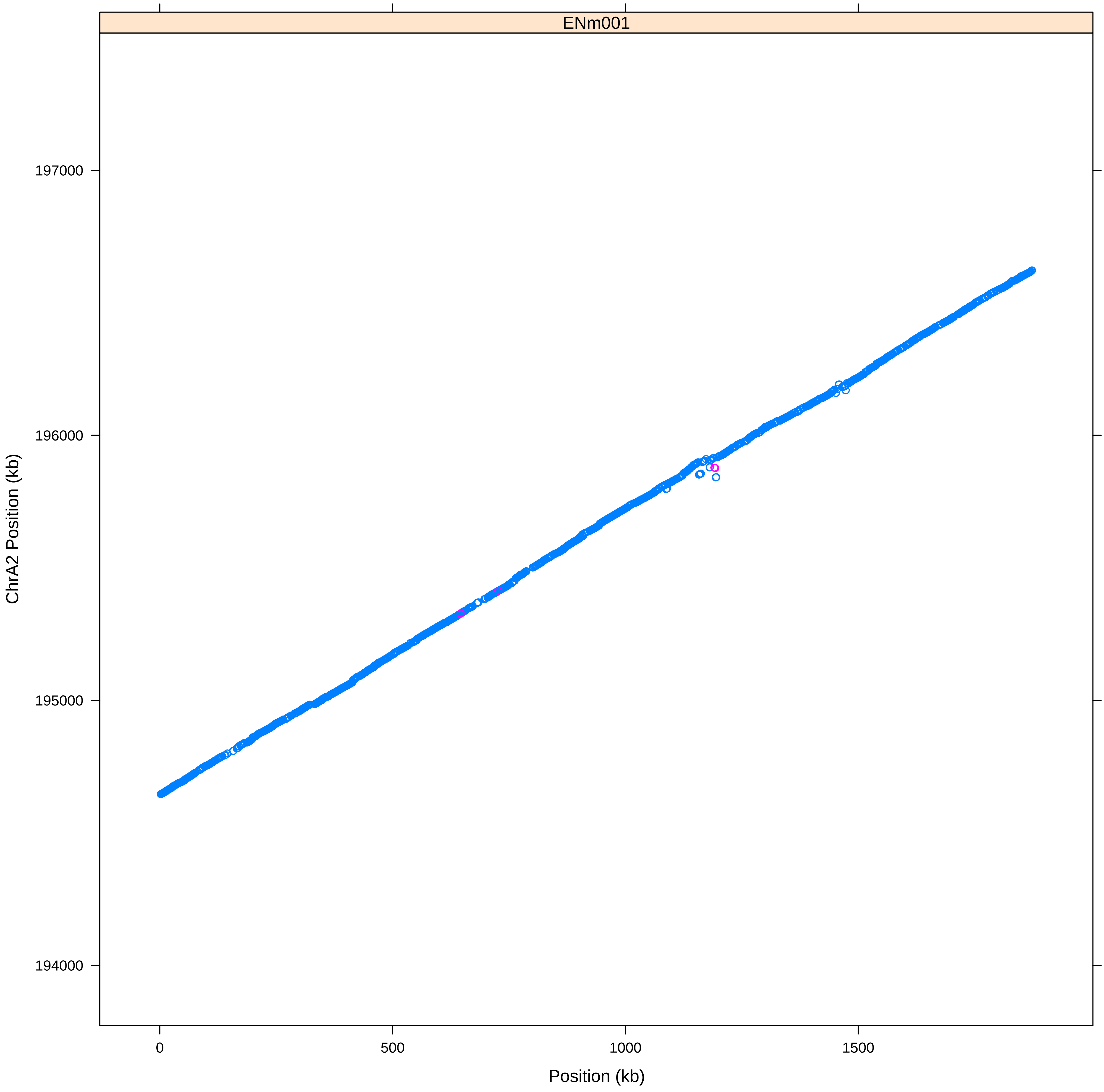
ENm001
197000
196000
195000
194000
ChrA2 Position (kb)
0
500
1000
1500
Position (kb)

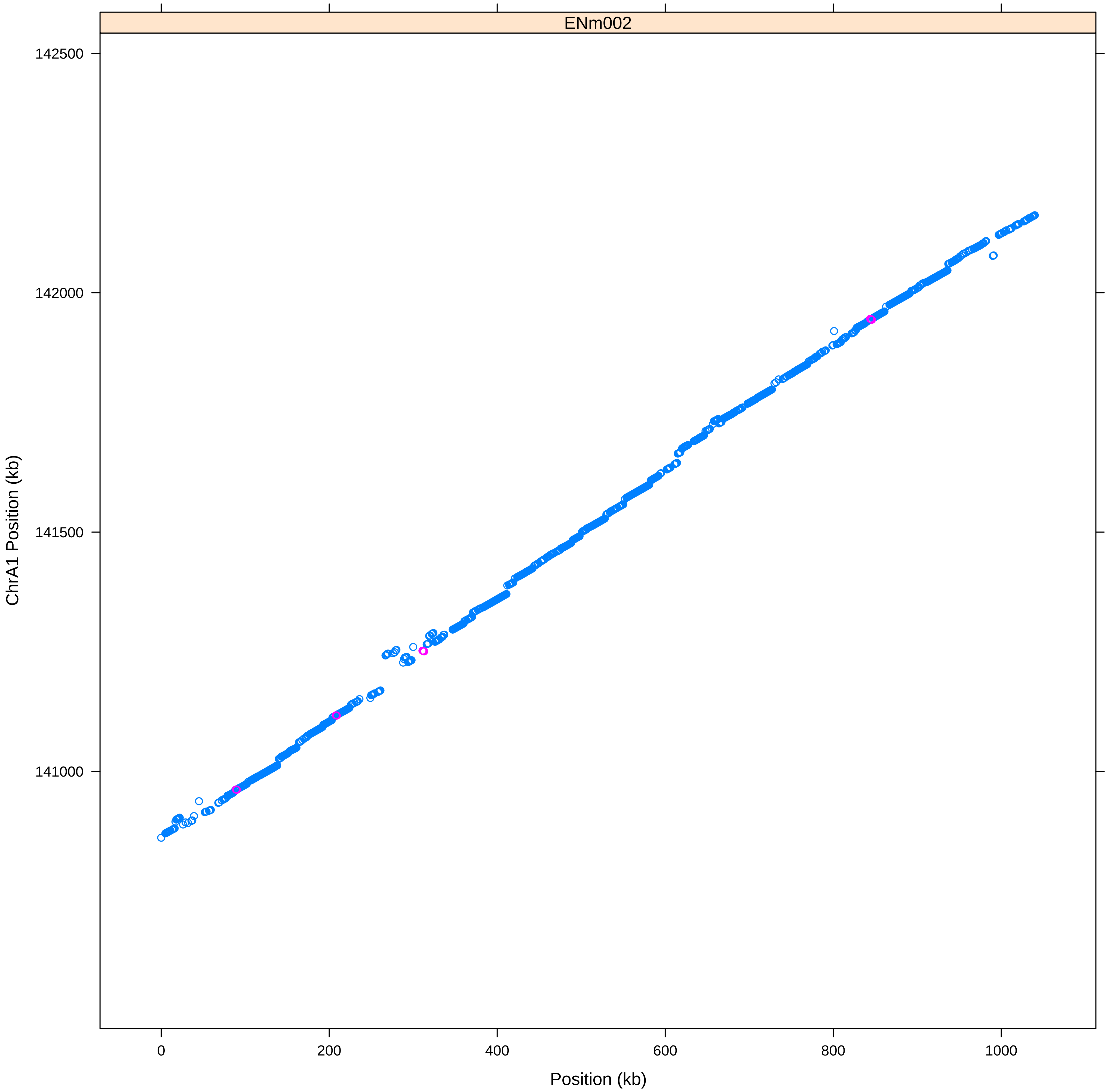

ENm002
ChrA1 Position (kb)
142500
142000
141500
141000
0
200
400
600
800
1000
Position (kb)

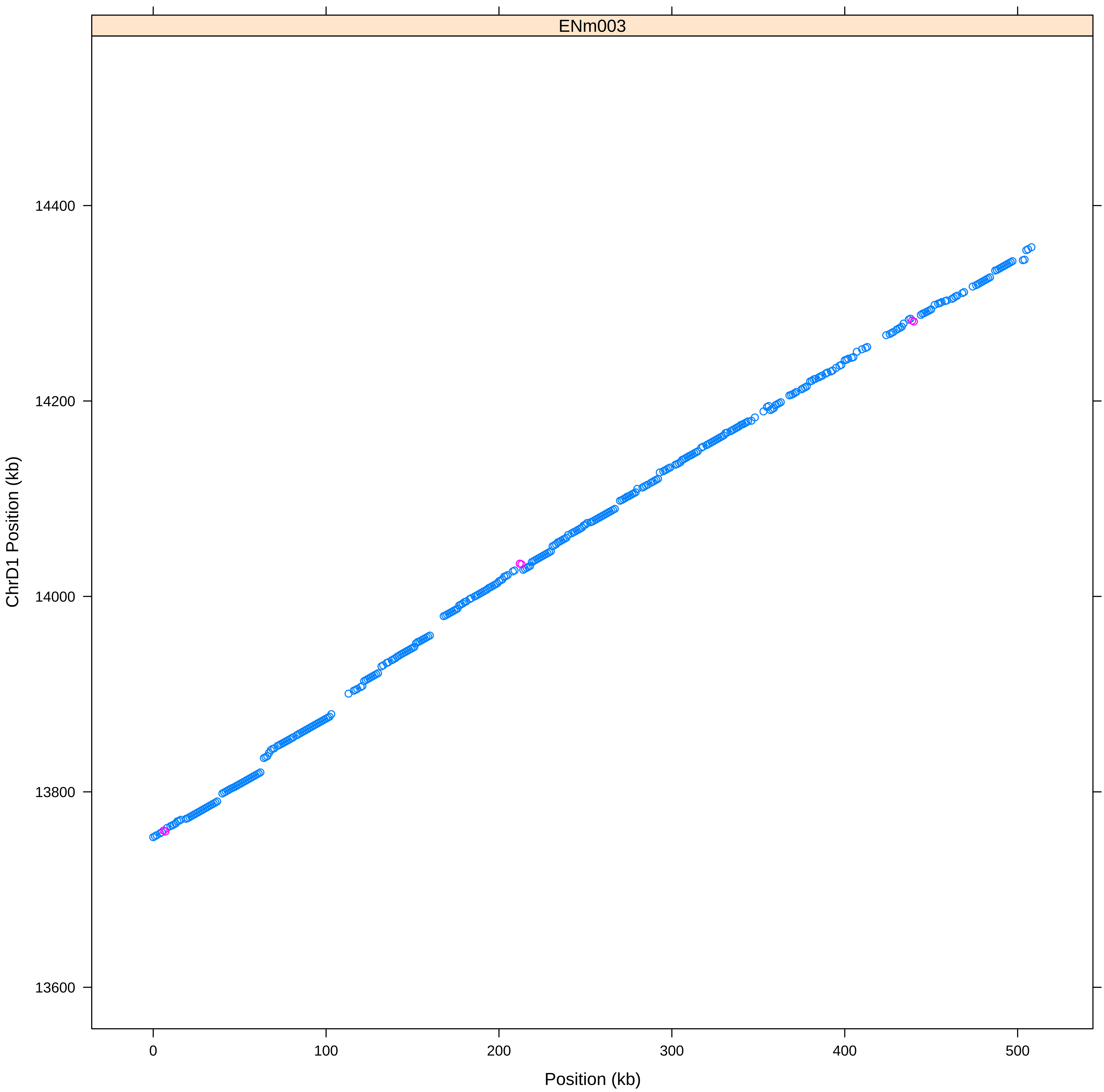

ENm003
ChrD1 Position (kb)
14400
14200
14000
13800
13600
Position (kb)
0
100
200
300
400
500

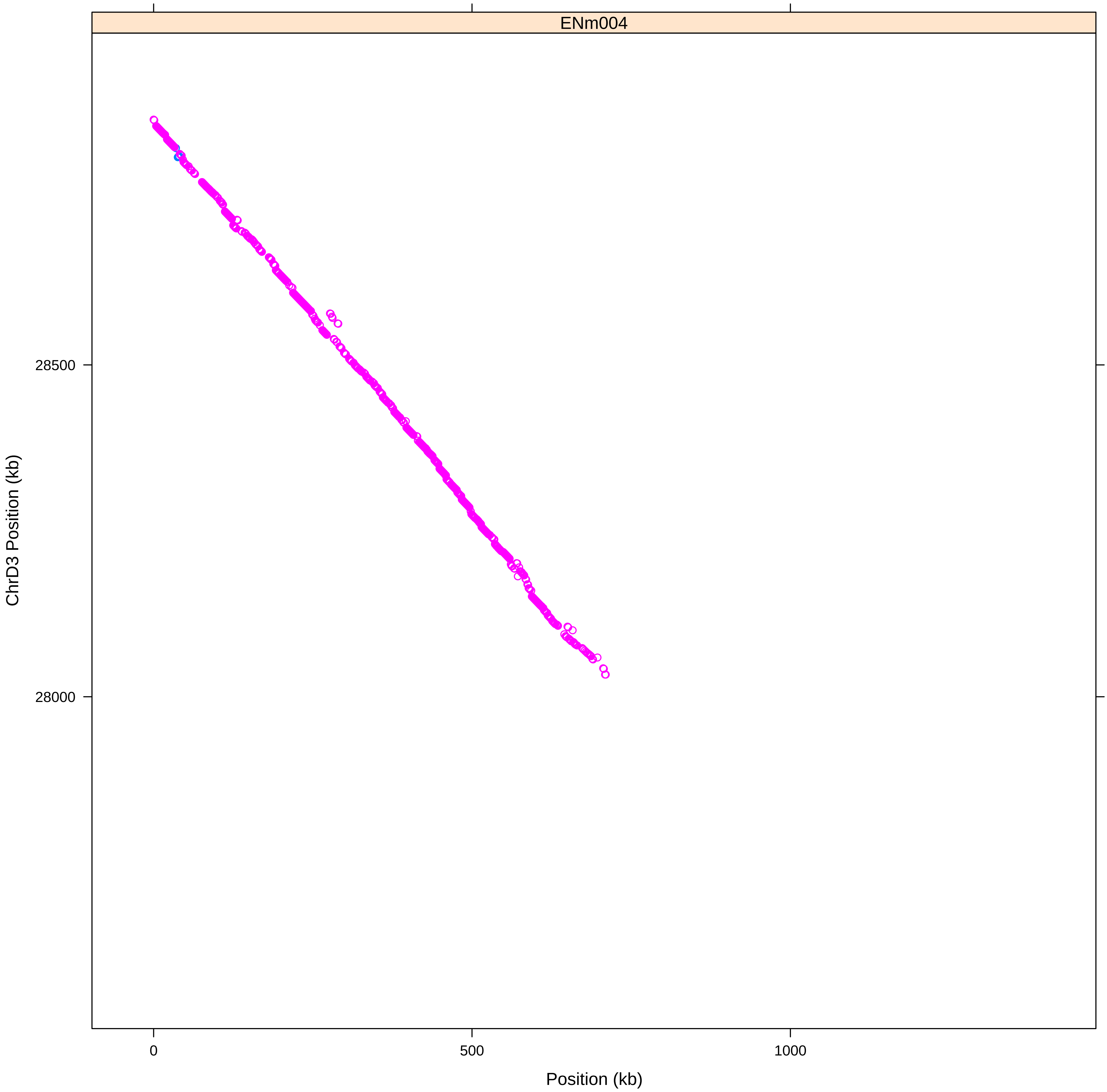

ENm004
ChrD3 Position (kb)
28500
28000
0
500
1000
Position (kb)

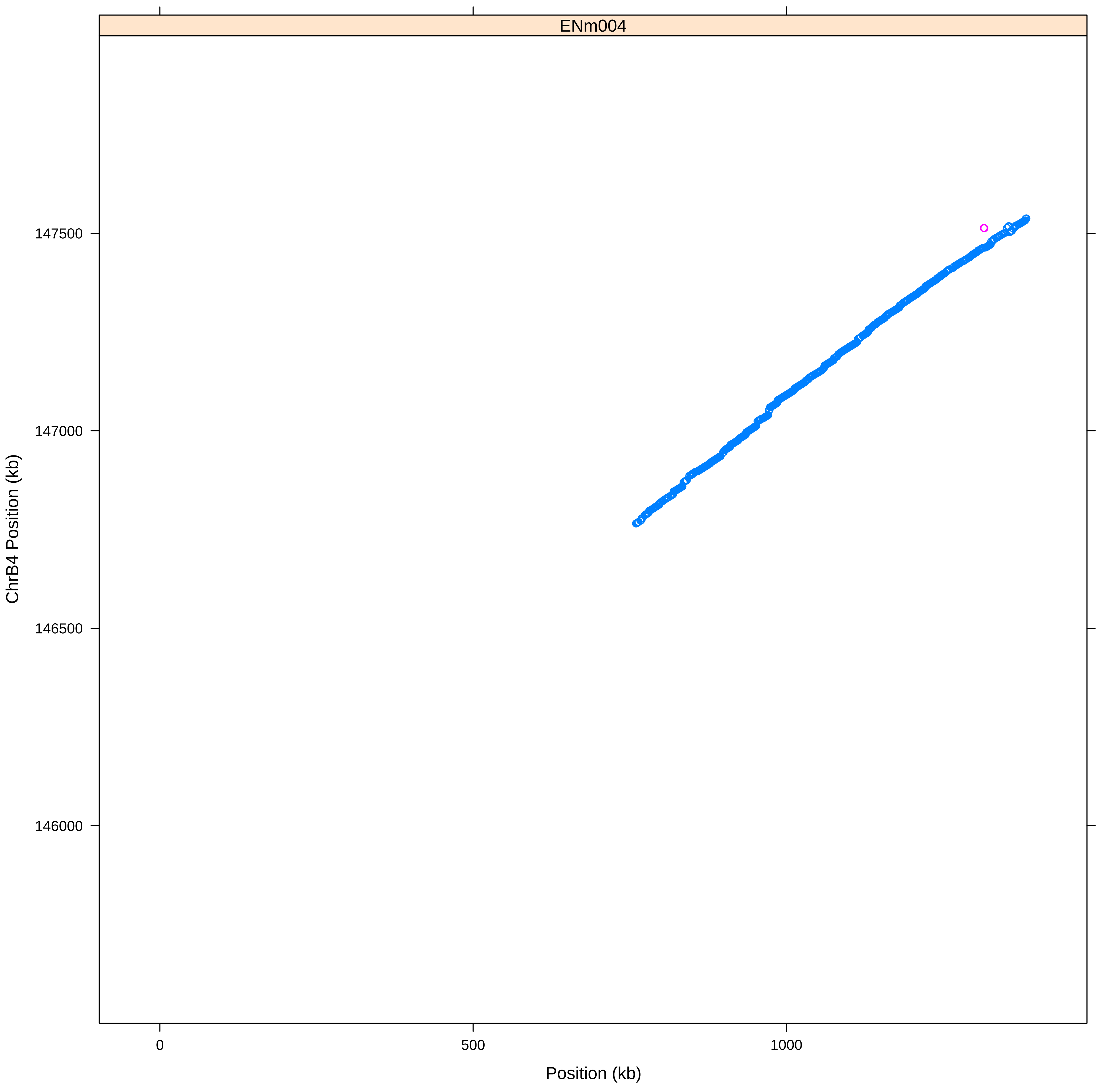

ENm004
ChrB4 Position (kb)
147500
147000
146500
146000
0
500
1000
Position (kb)

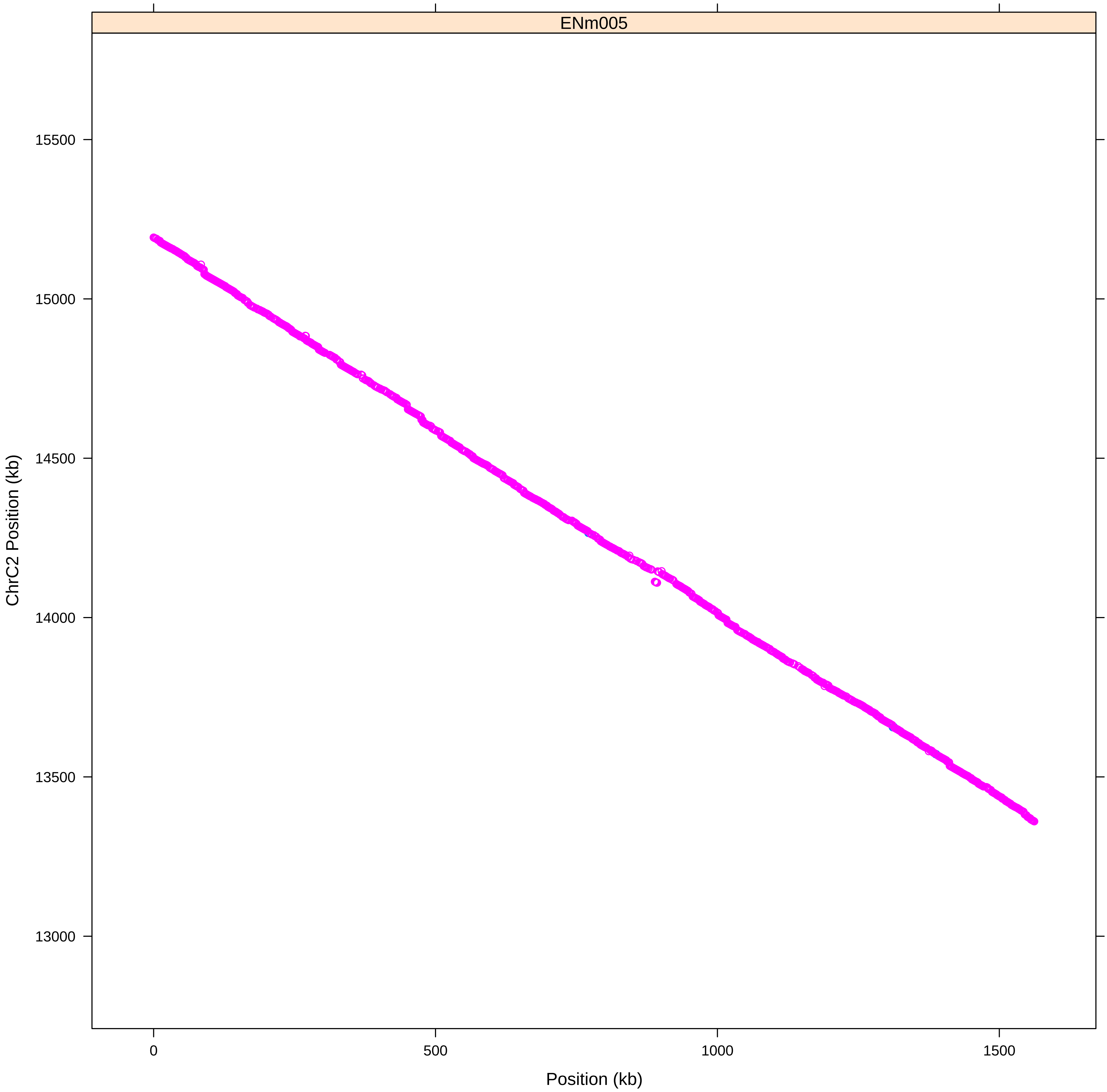
ENm005
15500
15000
14500
14000
13500
13000
ChrC2 Position (kb)
0
500
1000
1500
Position (kb)

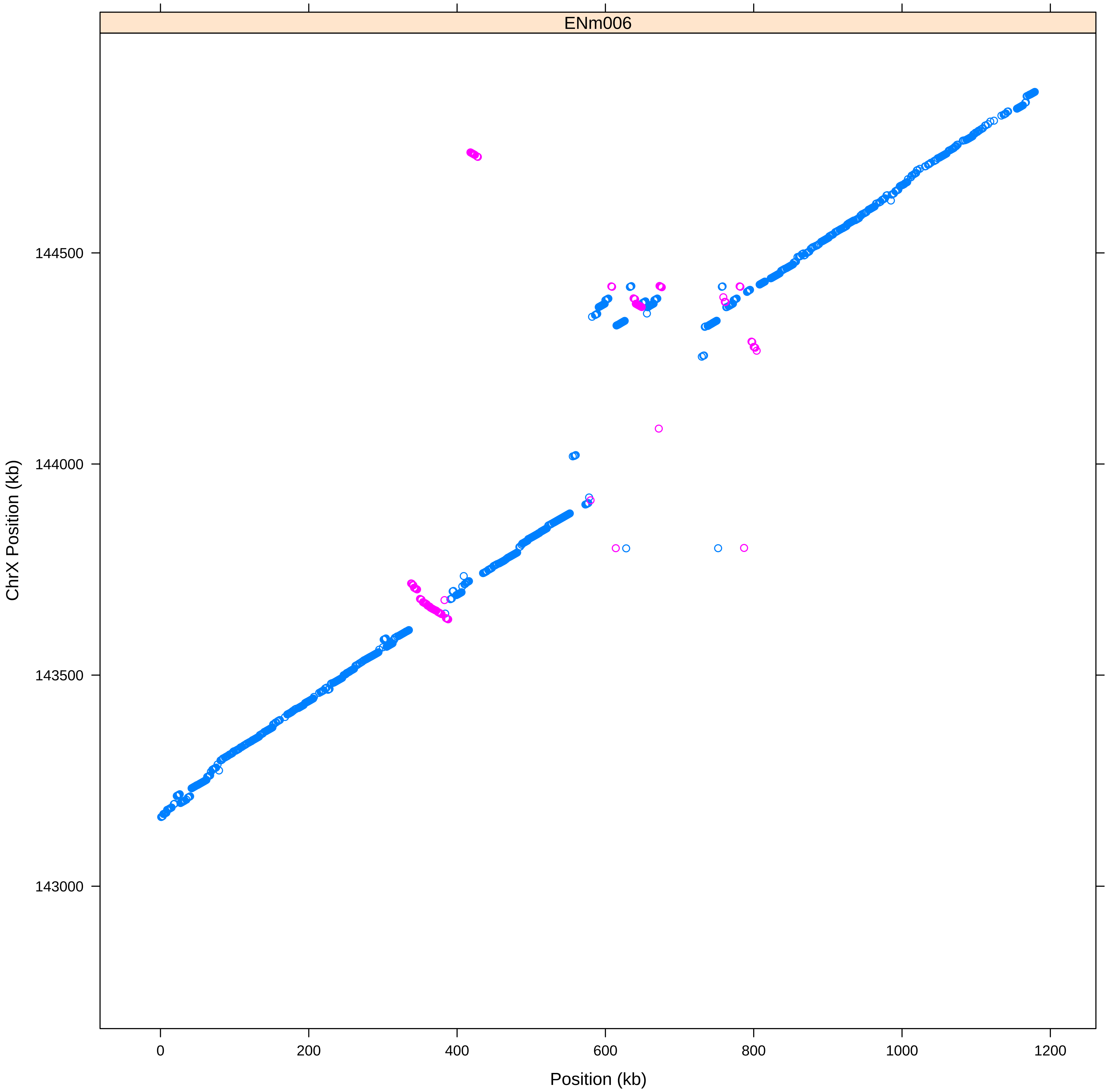

ENm006
ChrX Position (kb)
144500
144000
143500
143000
0
200
400
600
800
1000
1200
Position (kb)

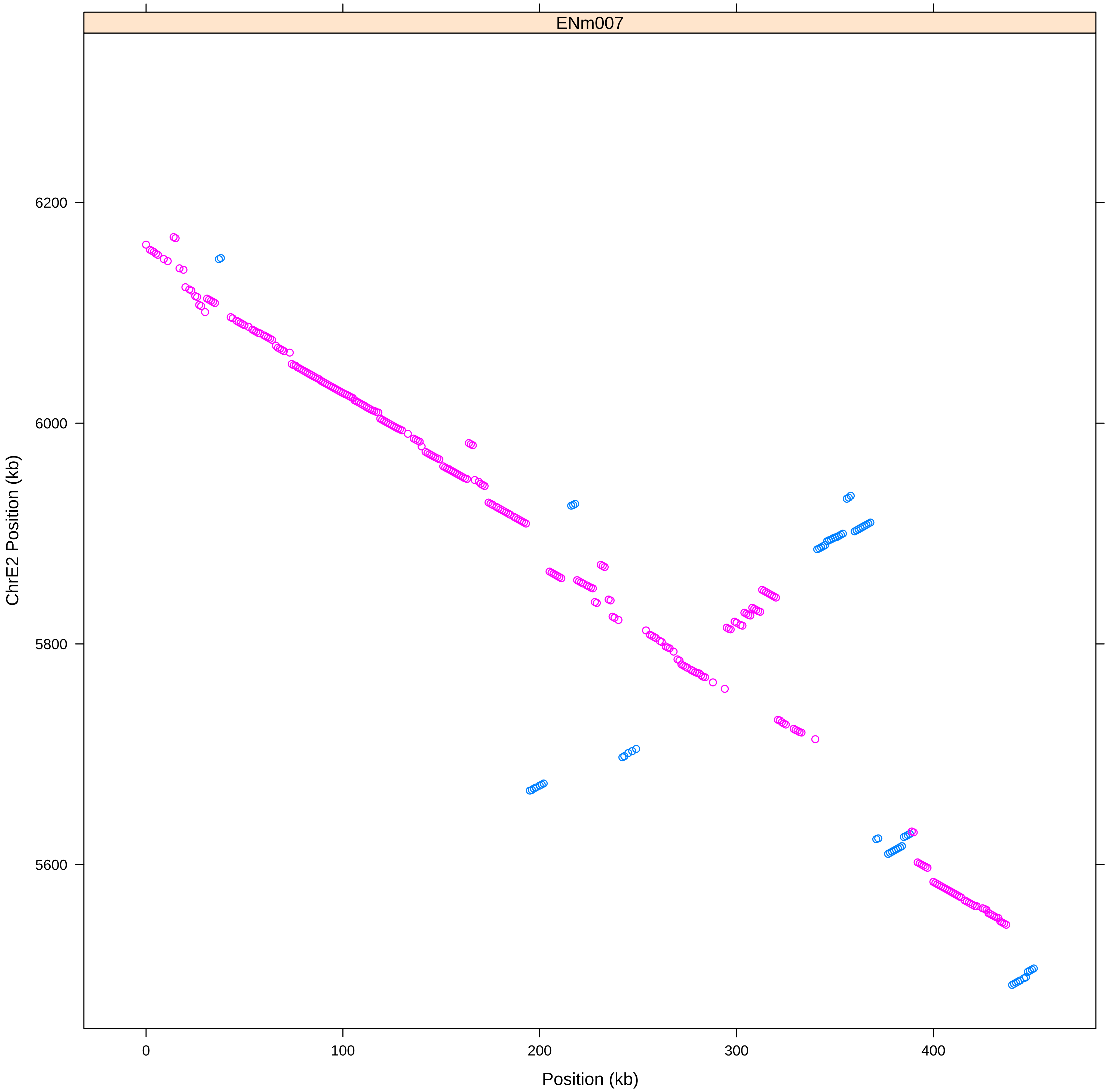
ENm007
ChrE2 Position (kb)
6200
6000
5800
5600
0
100
200
300
400
Position (kb)

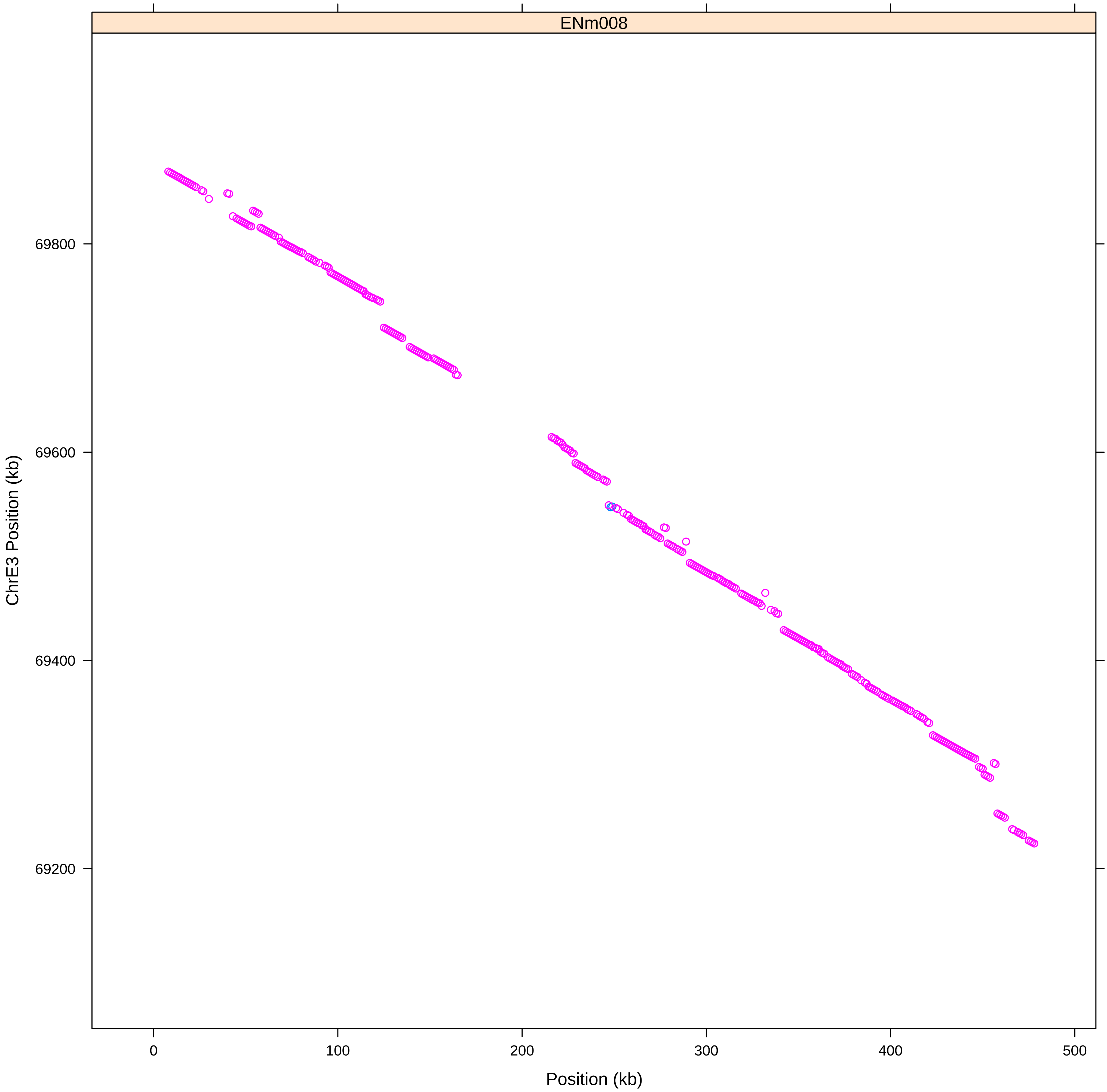
ENm008
ChrE3 Position (kb)
69800
69600
69400
69200
0
100
200
300
400
500
Position (kb)

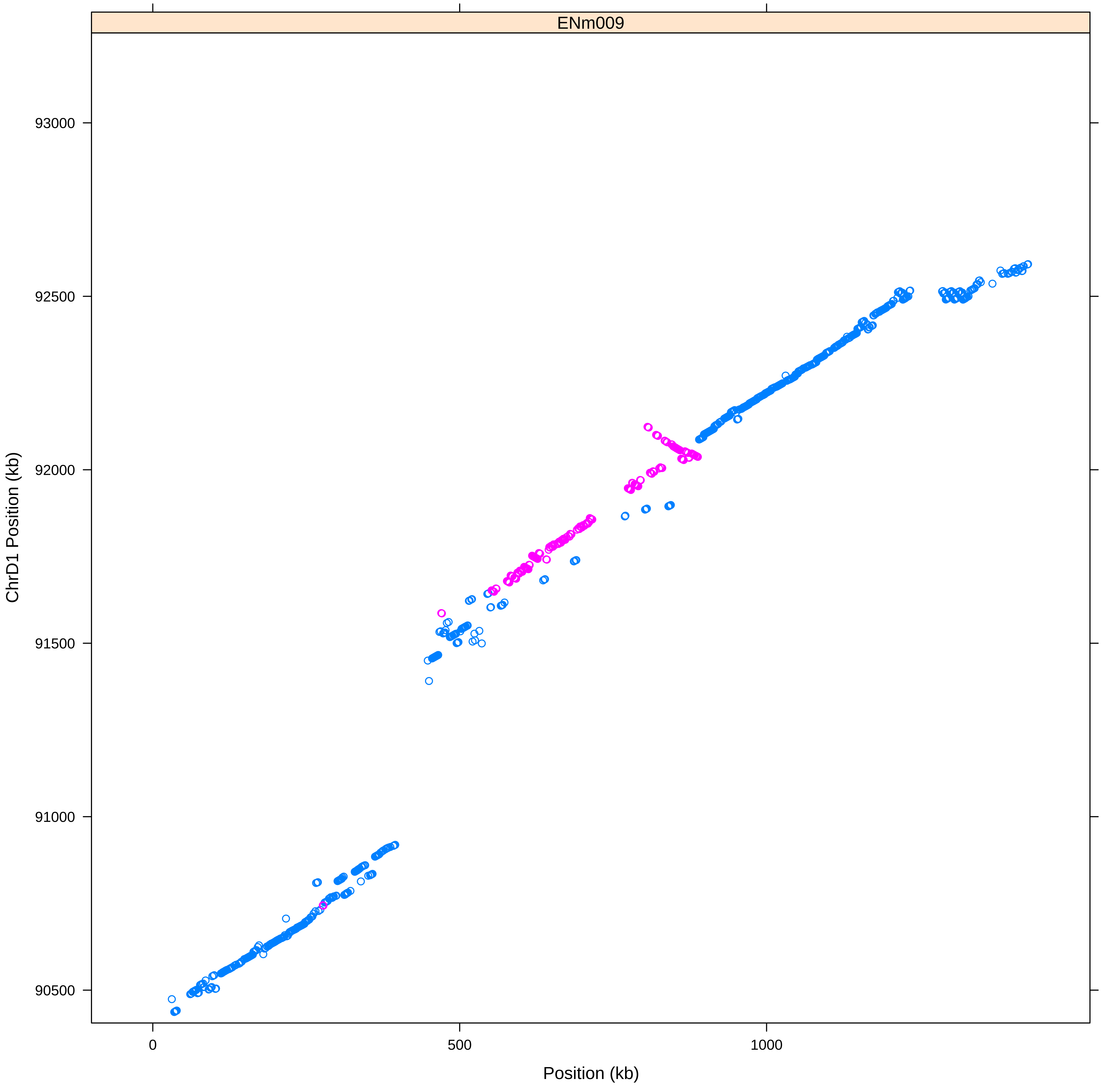
ENm009
ChrD1 Position (kb)
93000
92500
92000
91500
91000
90500
0
500
1000
Position (kb)

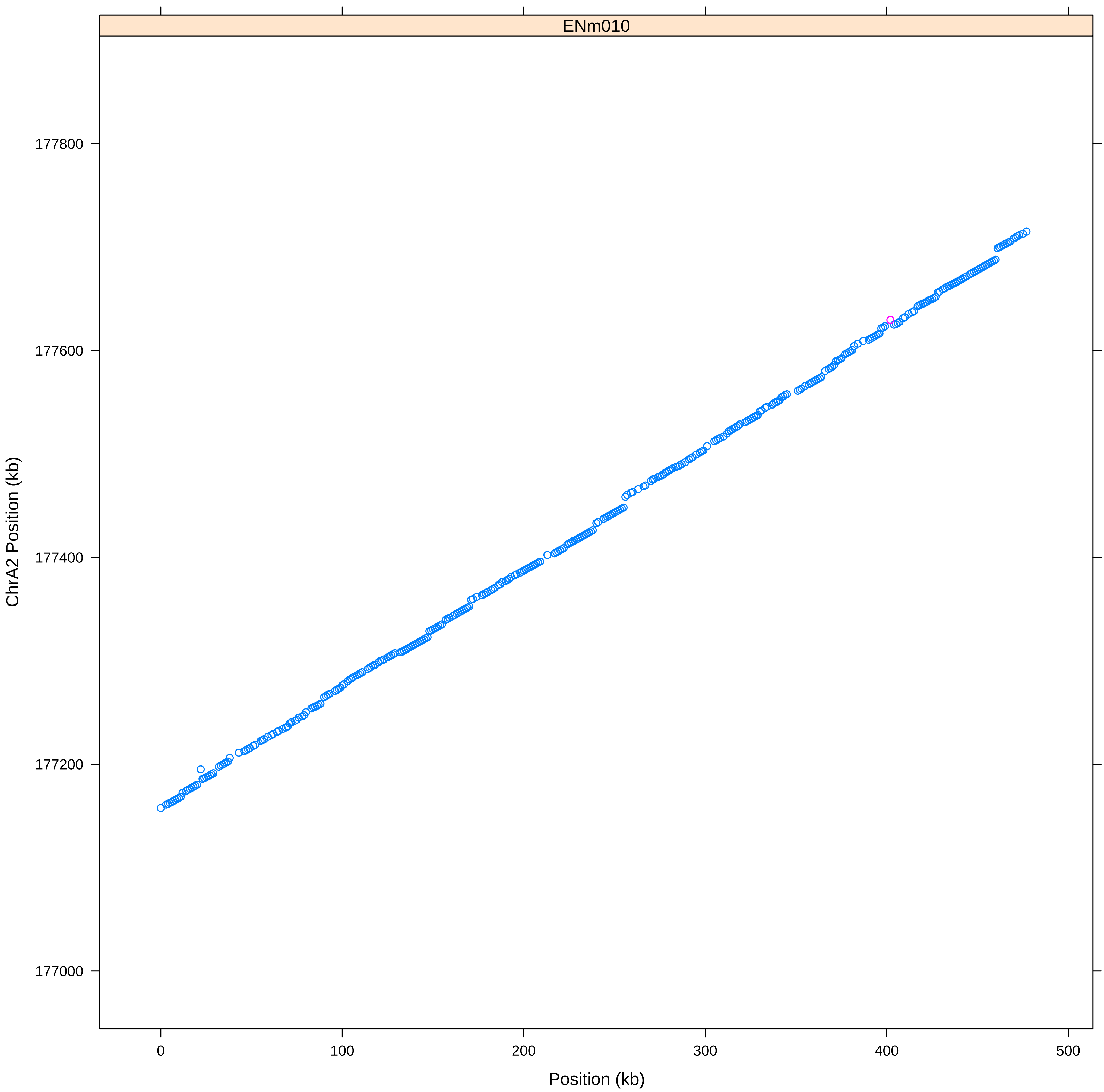

ENm010
ChrA2 Position (kb)
177800
177600
177400
177200
177000
Position (kb)
0
100
200
300
400
500

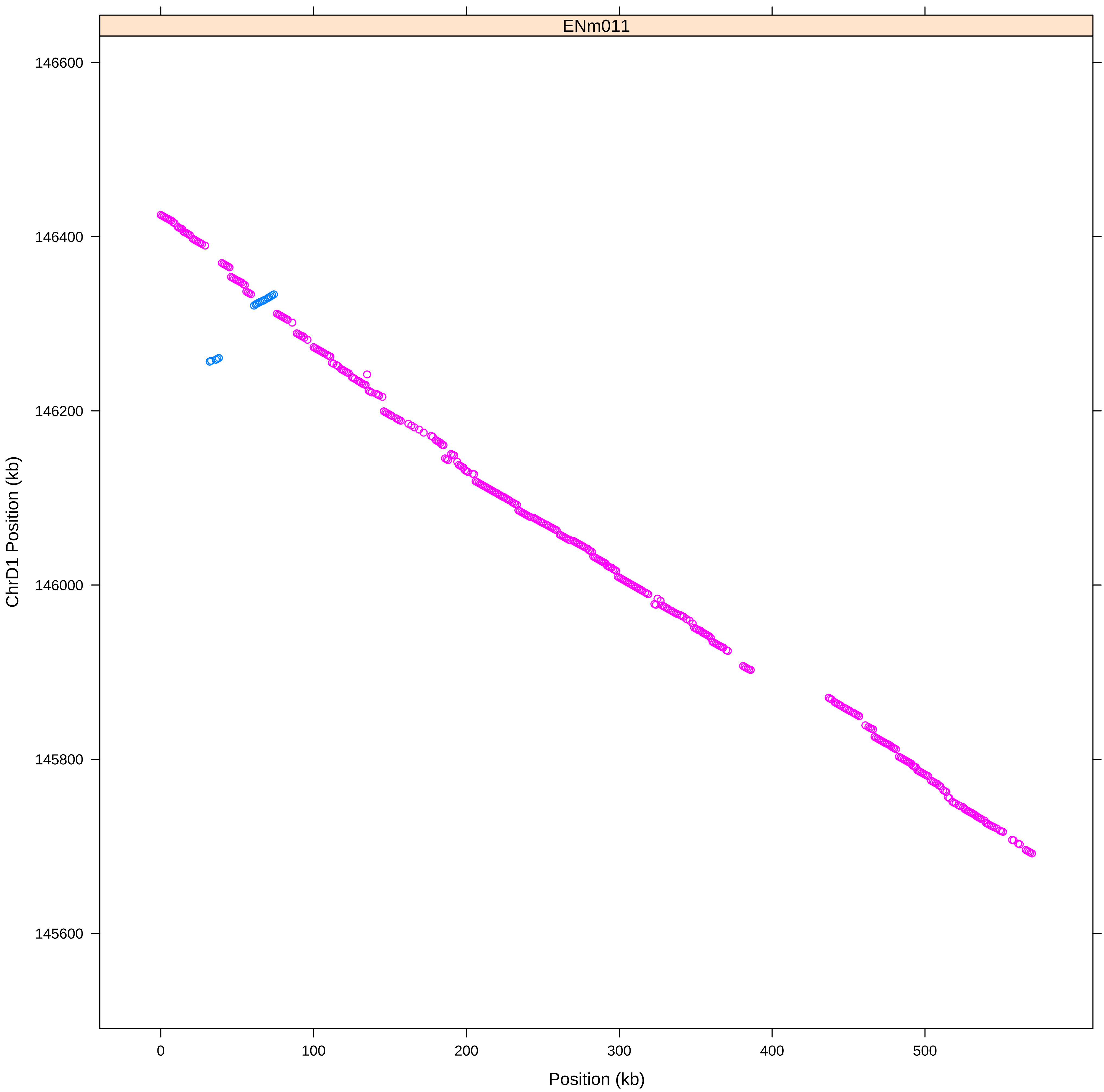
ENm011
ChrD1 Position (kb)
146600
146400
146200
146000
145800
145600
0
100
200
300
400
500
Position (kb)

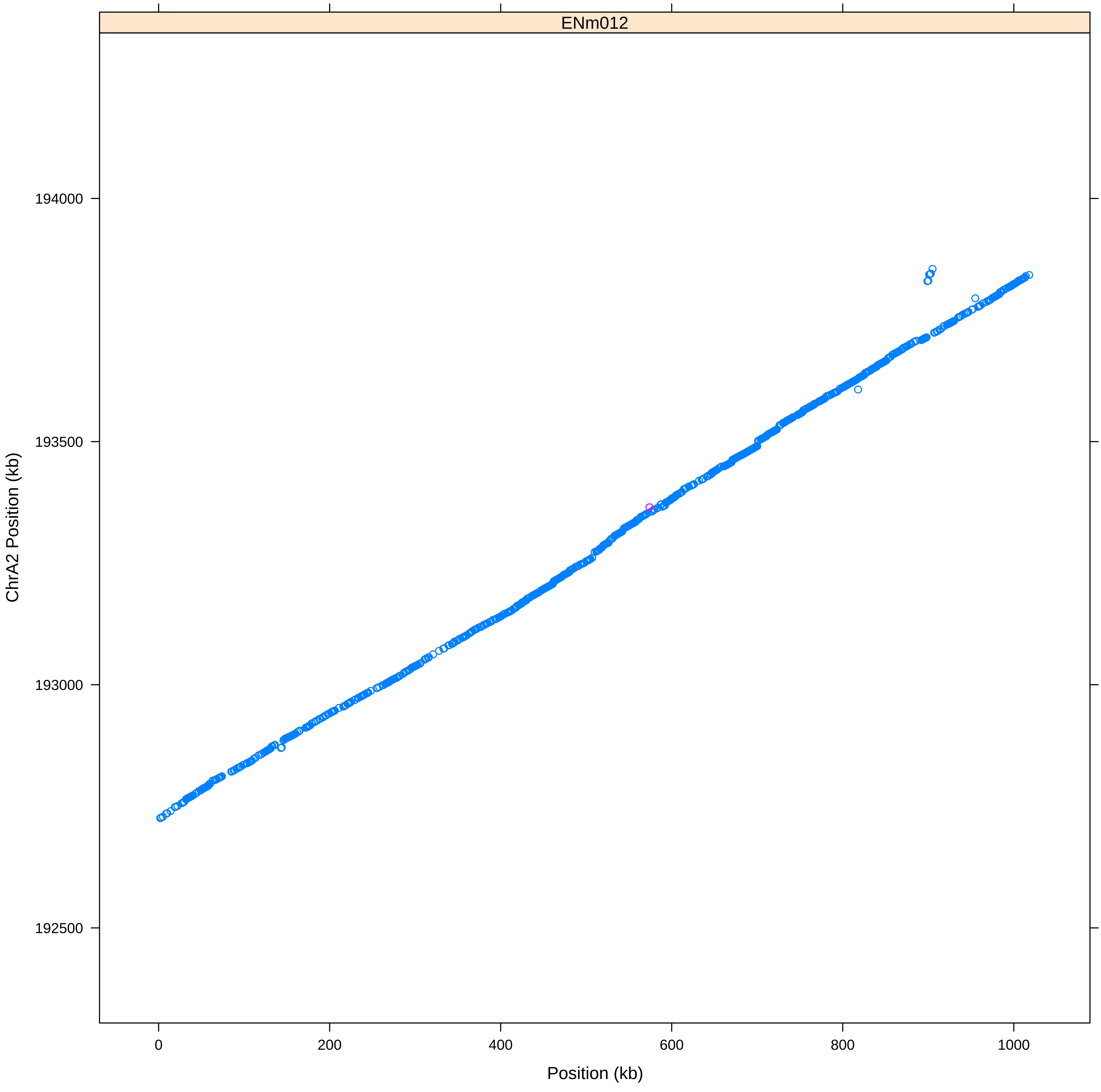
ENm012
ChrA2 Position (kb)
194000
193500
193000
192500
0
200
400
600
800
1000
Position (kb)

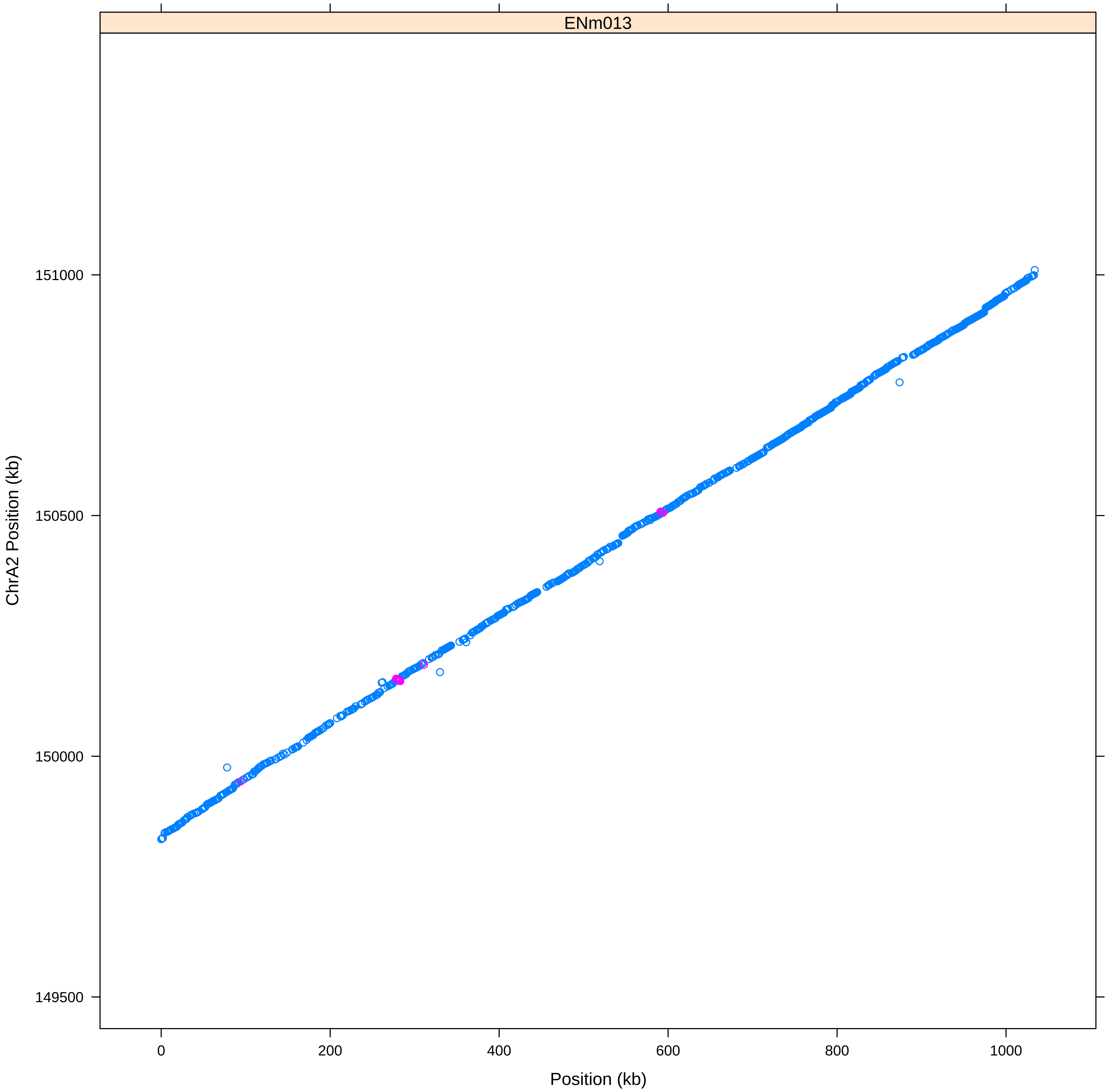

ENm013
ChrA2 Position (kb)
151000
150500
150000
149500
0
200
400
600
800
1000
Position (kb)

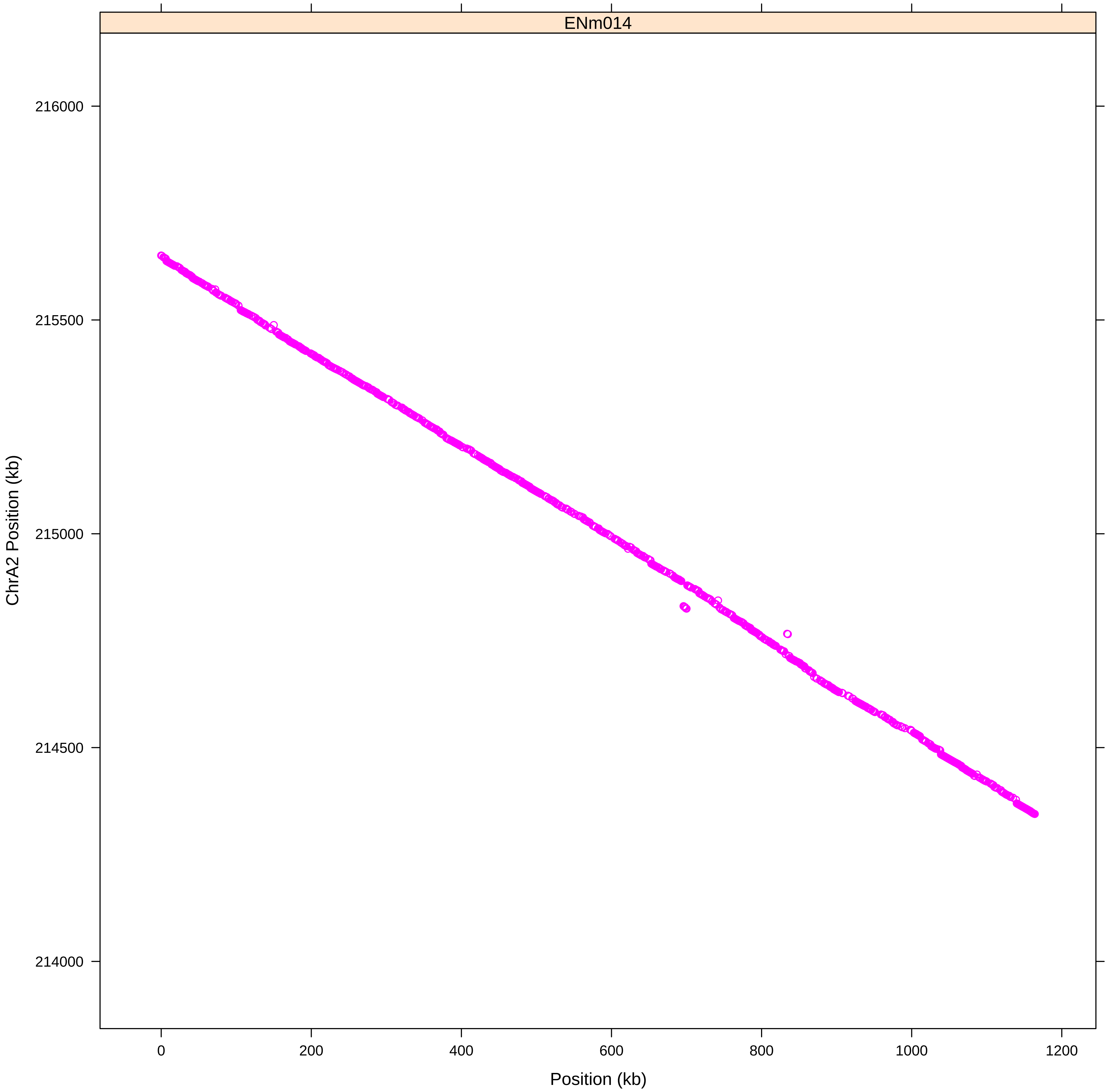

ENm014
ChrA2 Position (kb)
216000
215500
215000
214500
214000
Position (kb)
0
200
400
600
800
1000
1200

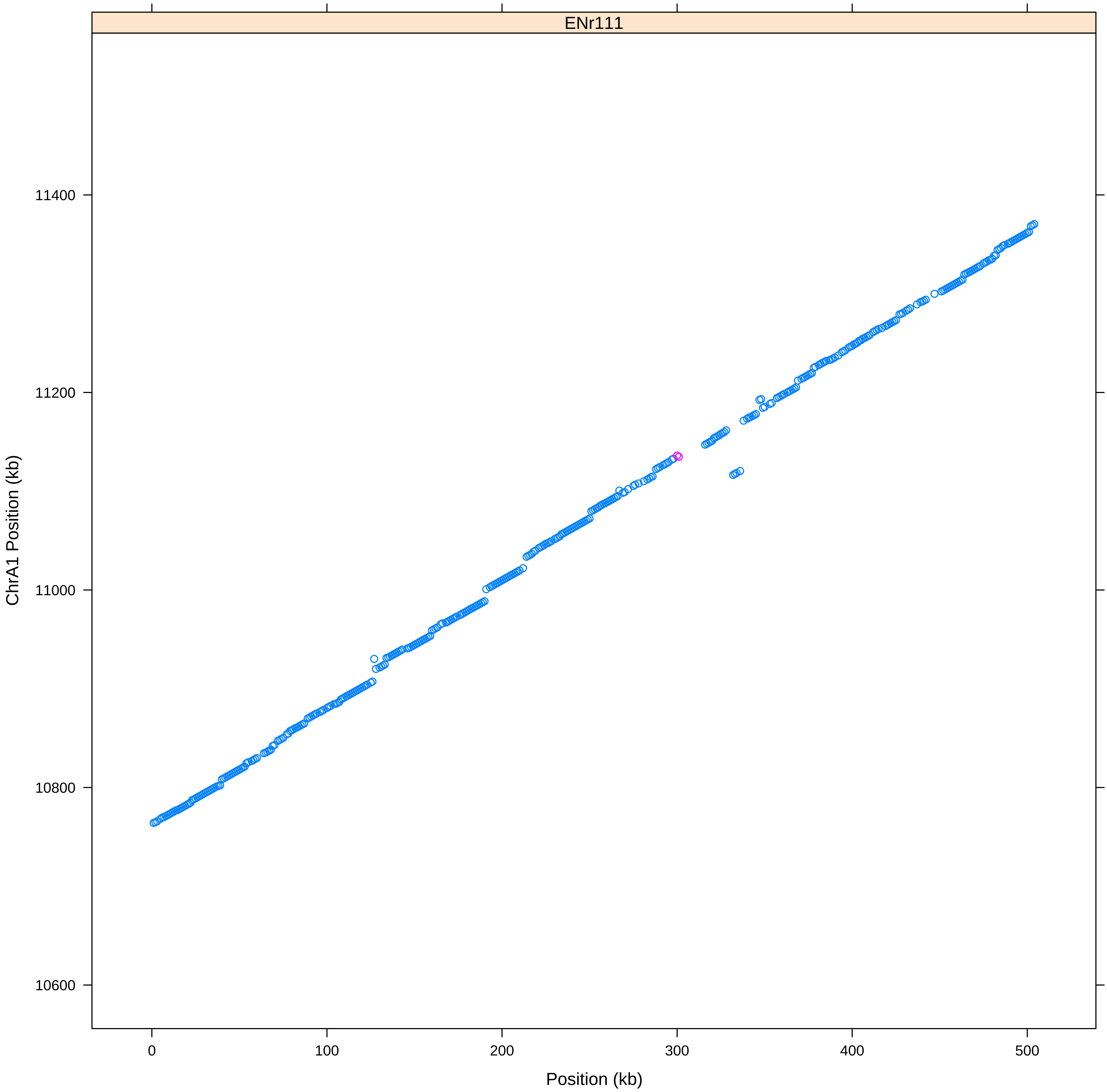
ENr111
ChrA1 Position (kb)
11400
11200
11000
10800
10600
0
100
200
300
400
500
Position (kb)

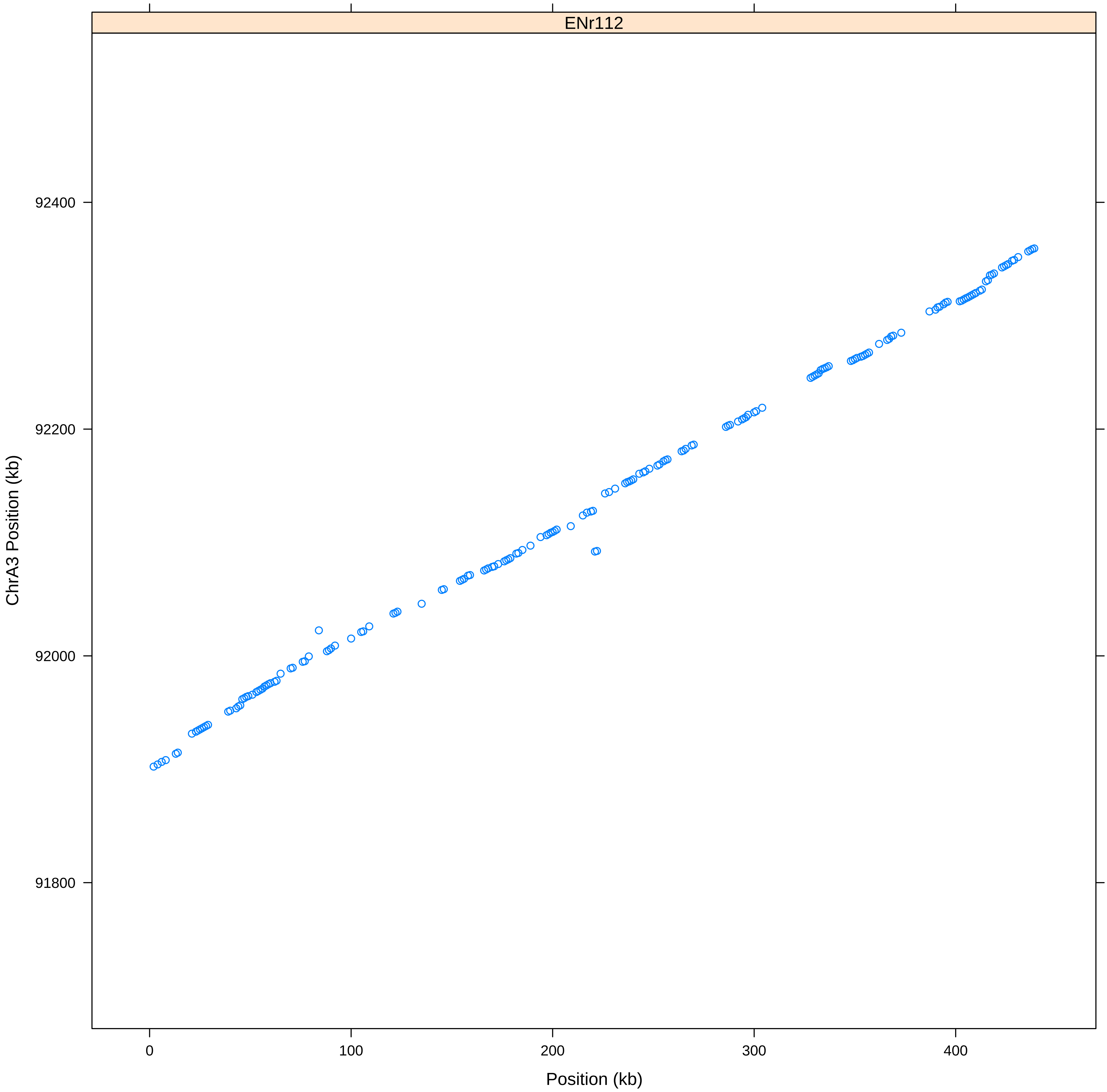
ENr112
ChrA3 Position (kb)
92400
92200
92000
91800
Position (kb)
0
100
200
300
400

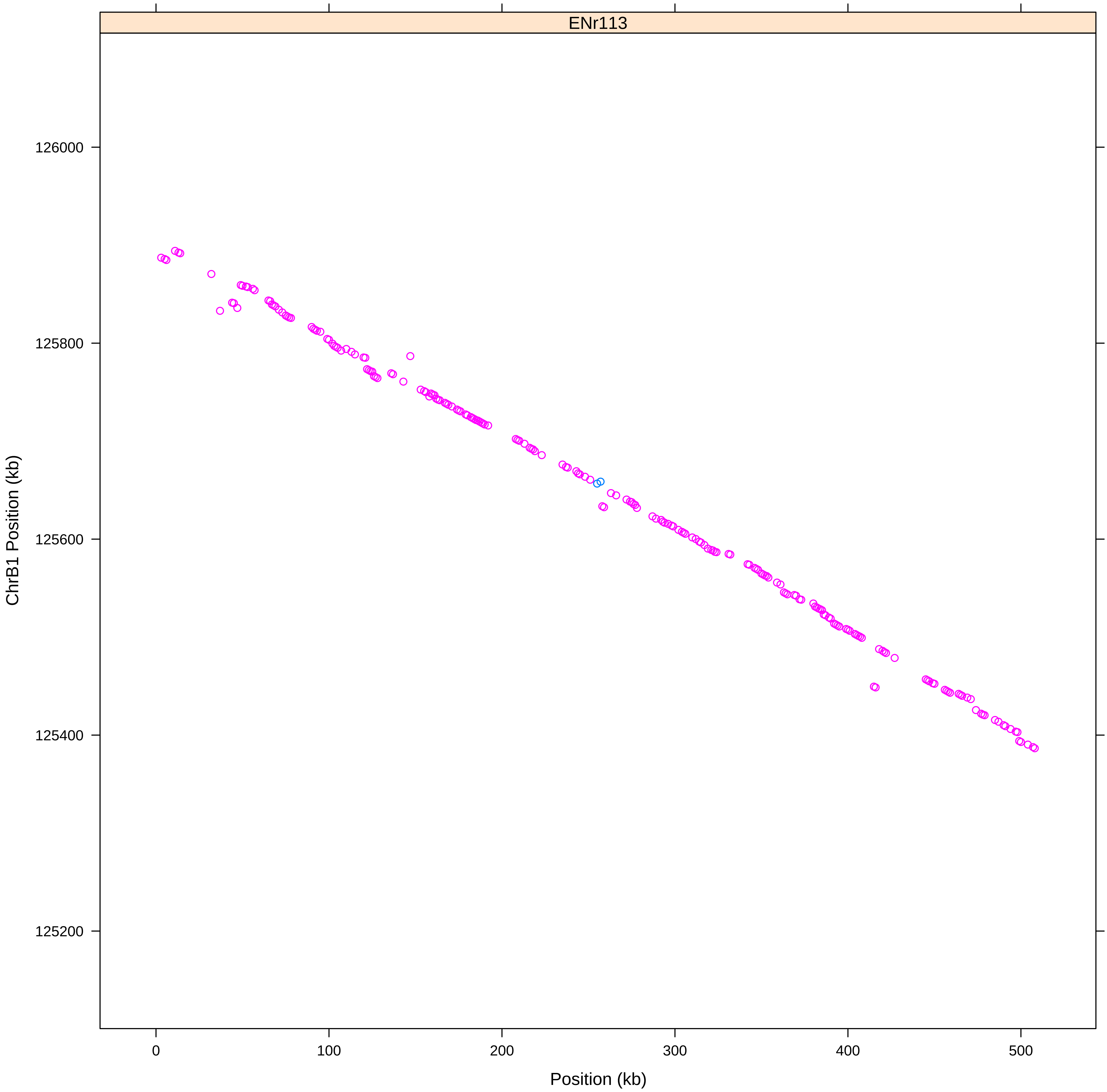
ENr113
ChrB1 Position (kb)
126000
125800
125600
125400
125200
0
100
200
300
400
500
Position (kb)

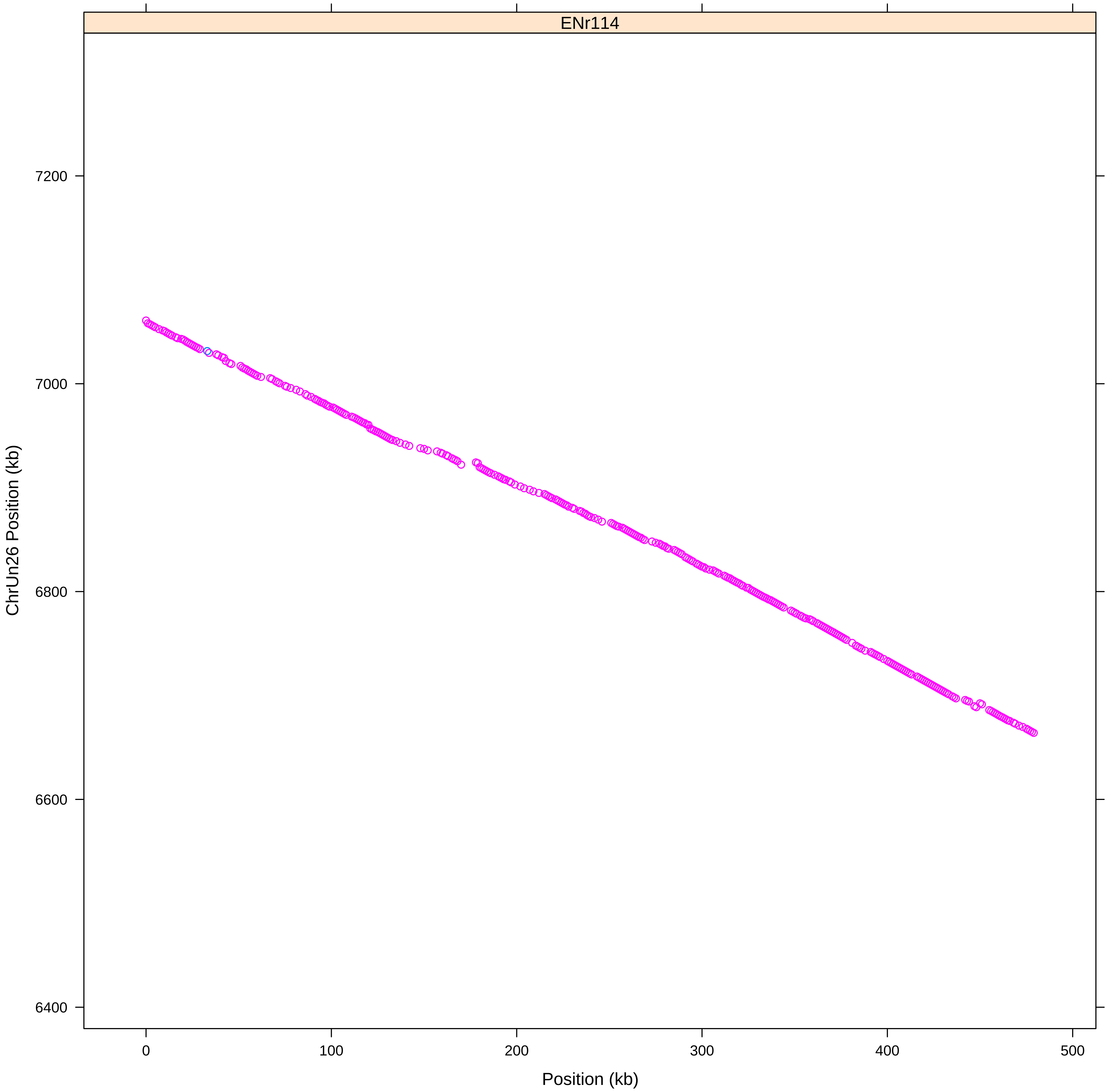
ENr114
ChrUn26 Position (kb)
7200
7000
6800
6600
6400
0
100
200
300
400
500
Position (kb)

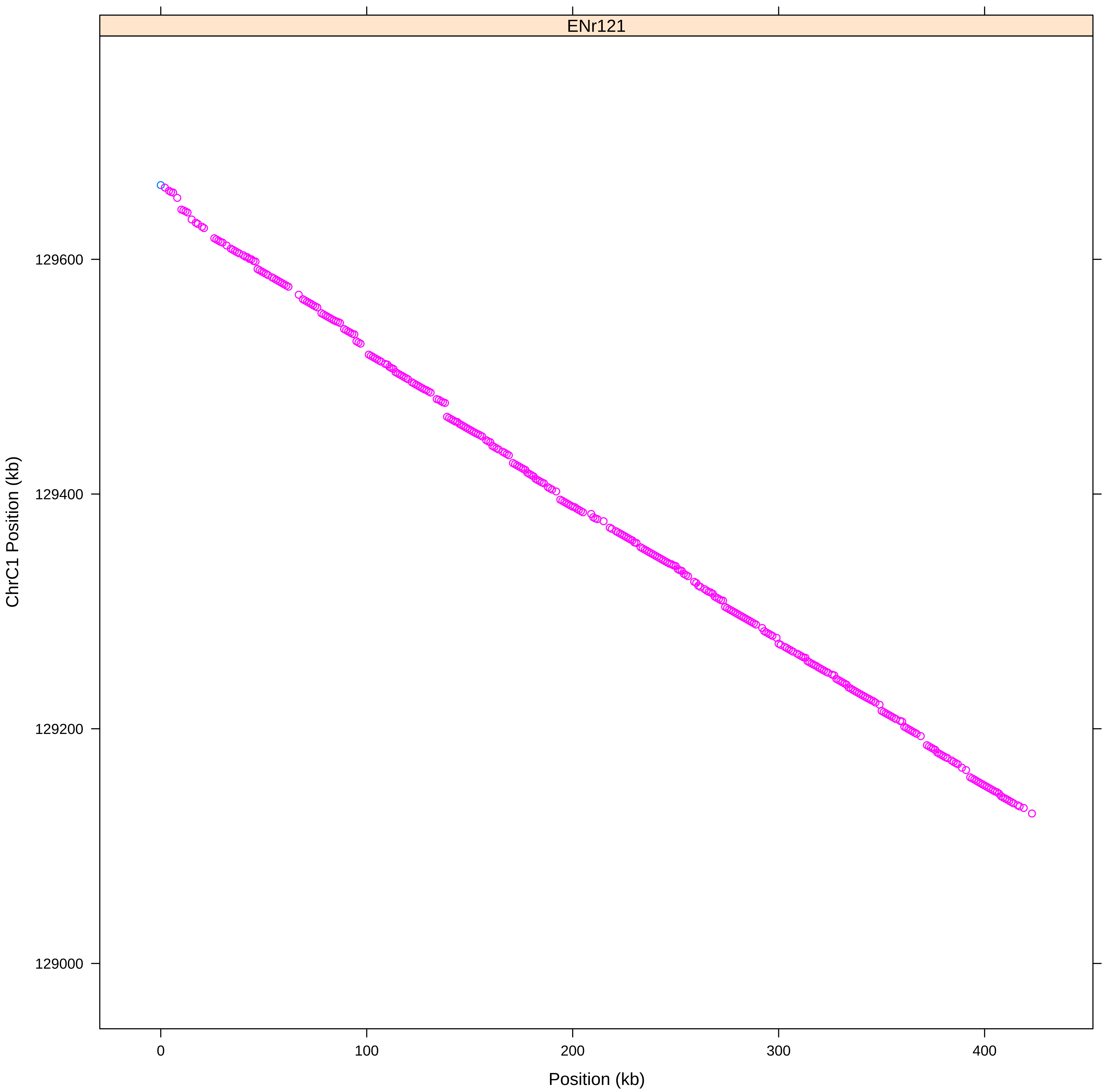

ENr121
ChrC1 Position (kb)
129600
129400
129200
129000
Position (kb)
0
100
200
300
400

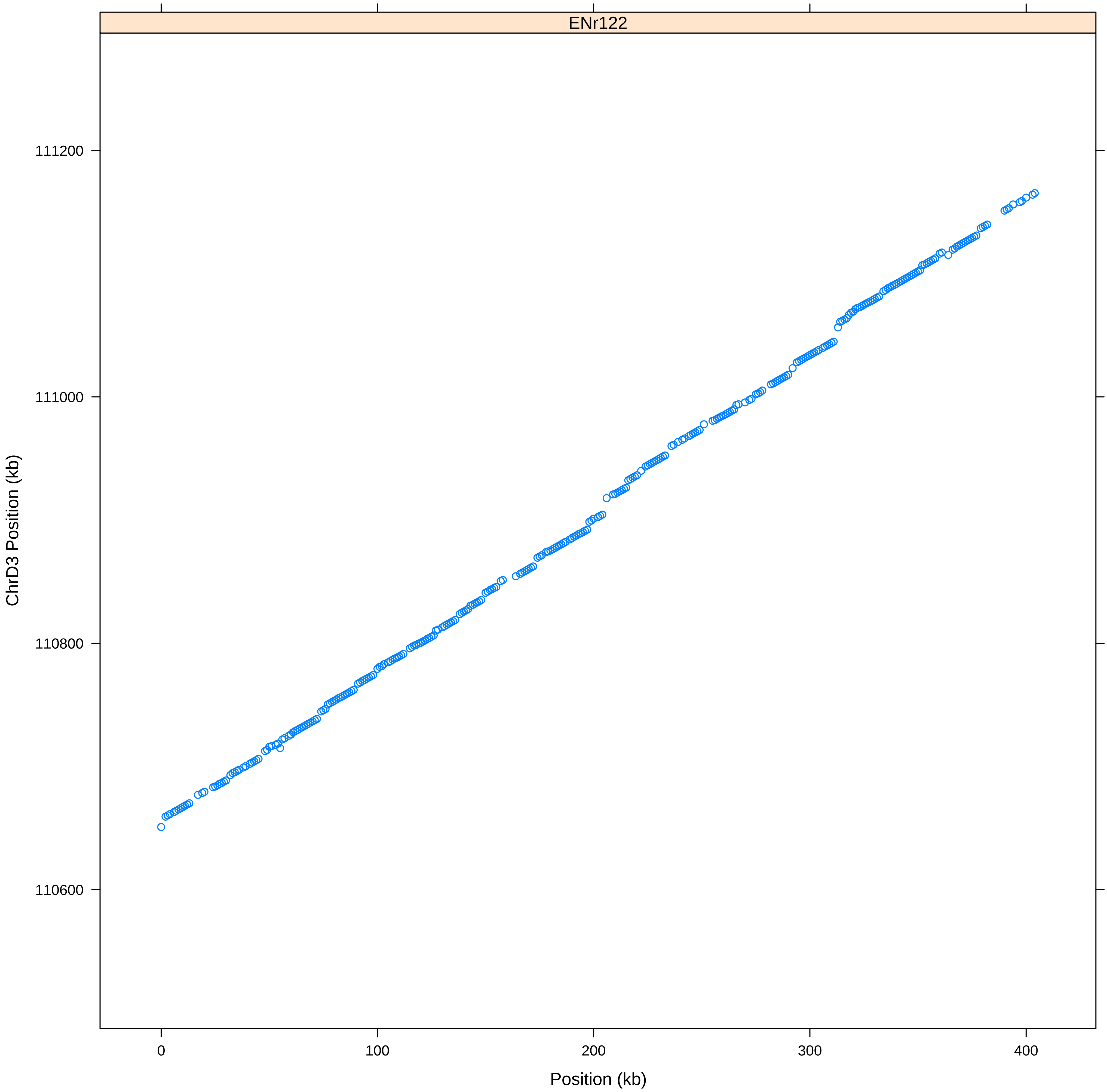
ENr122
ChrD3 Position (kb)
111200
111000
110800
110600
0
100
200
300
400
Position (kb)

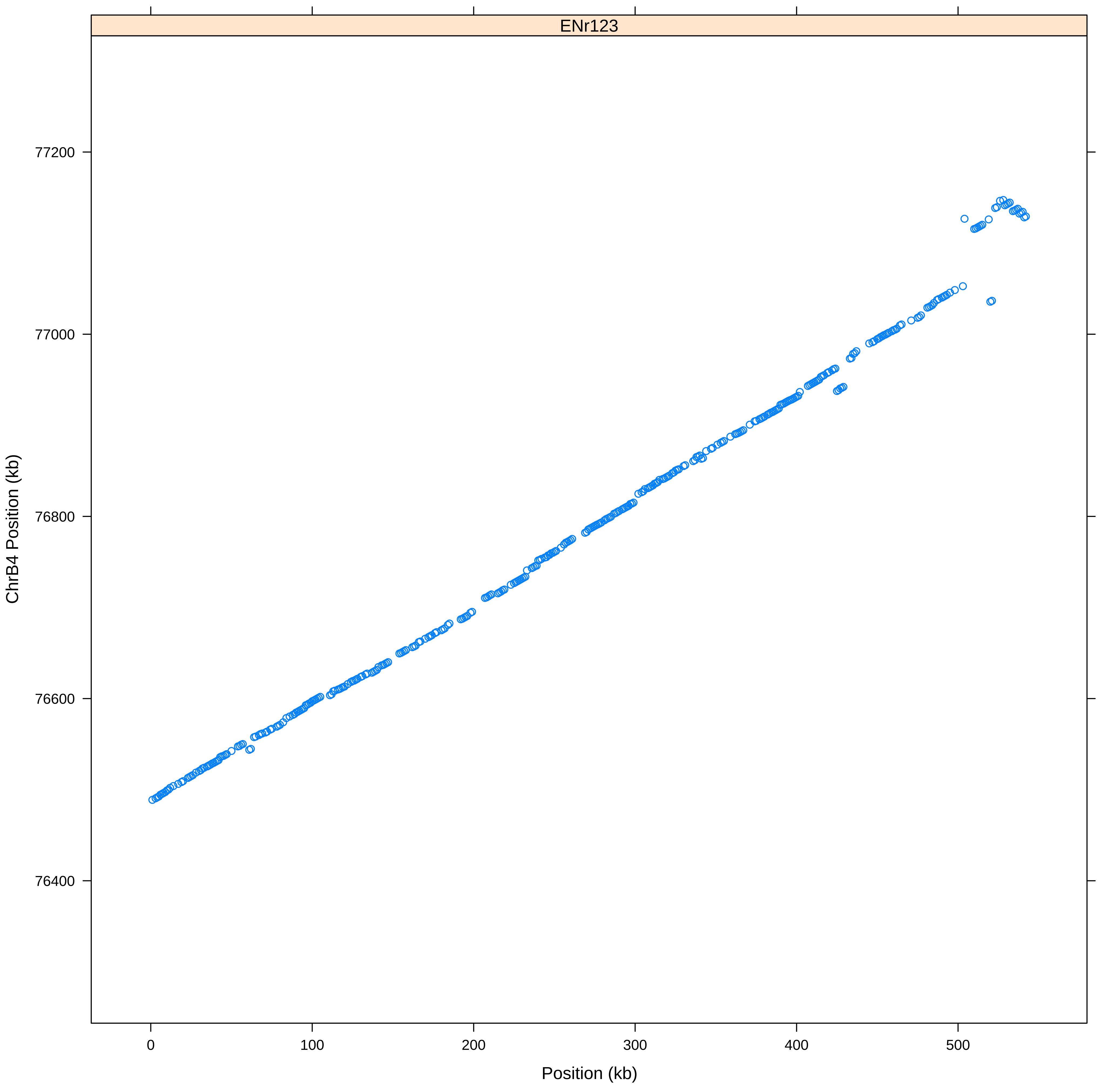
ENr123
ChrB4 Position (kb)
77200
77000
76800
76600
76400
0
100
200
300
400
500
Position (kb)

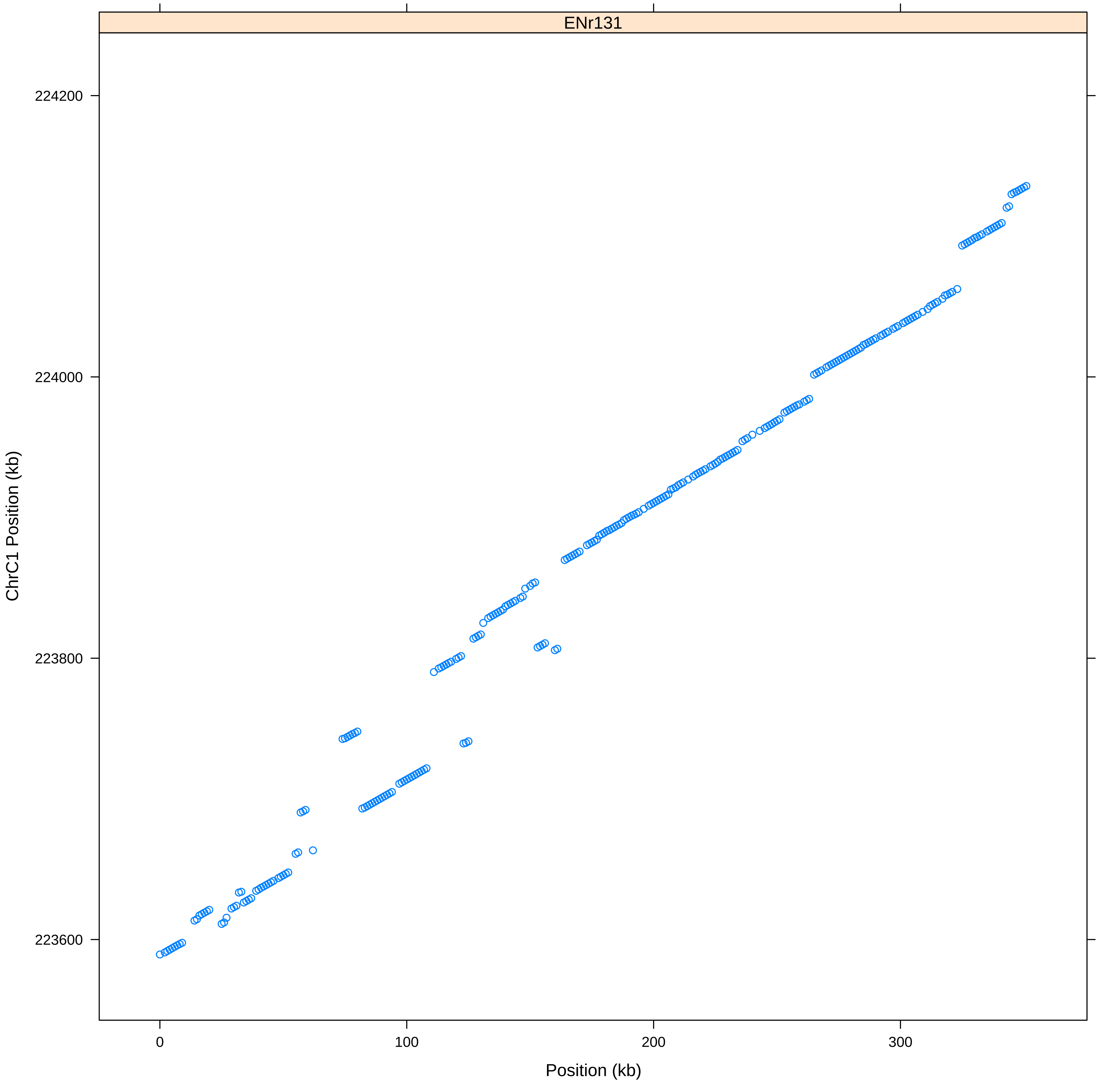
ENr131
224200
224000
223800
223600
ChrC1 Position (kb)
0
100
200
300
Position (kb)

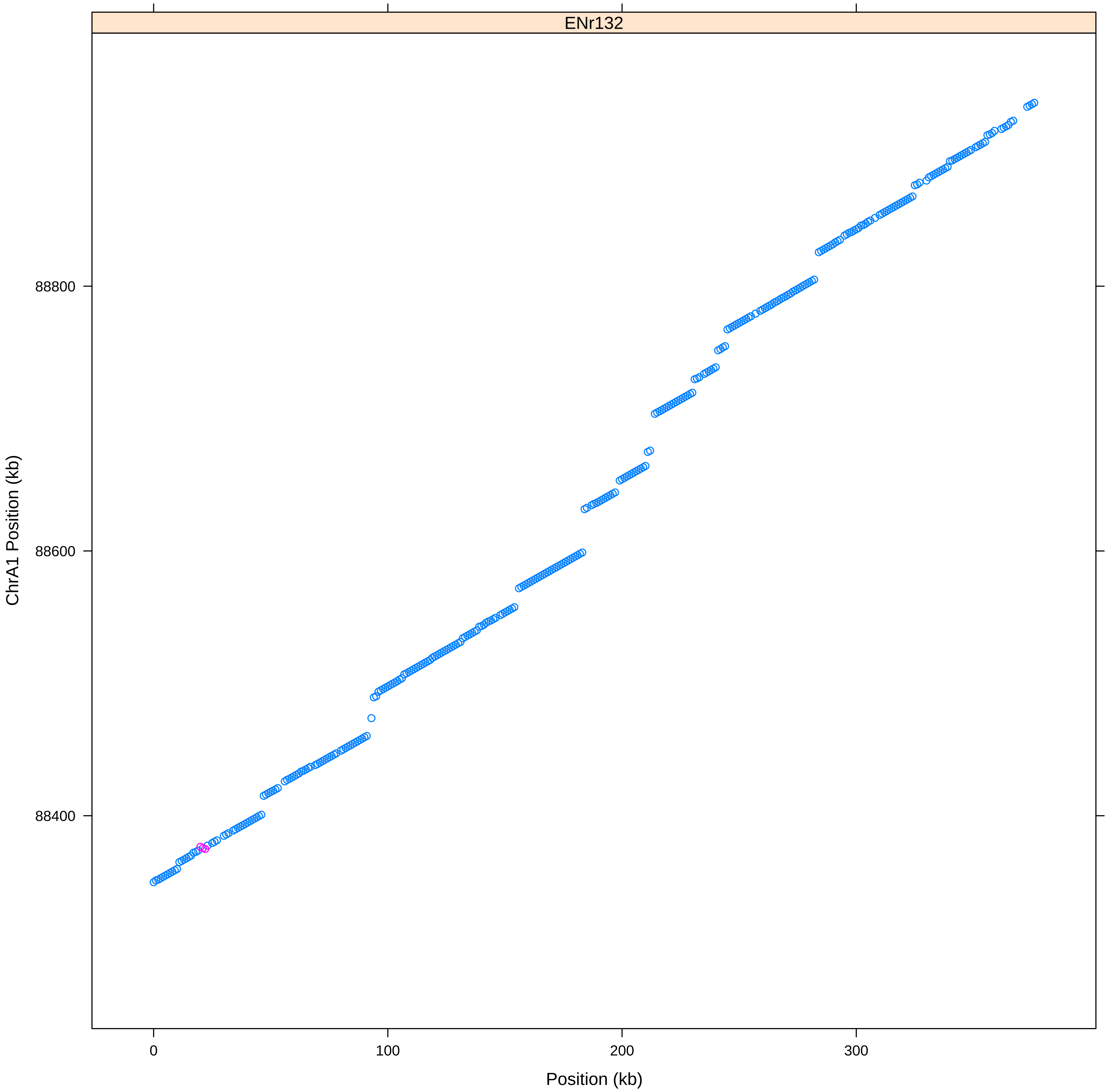

ENr132
ChrA1 Position (kb)
88400
88600
88800
Position (kb)
0
100
200
300

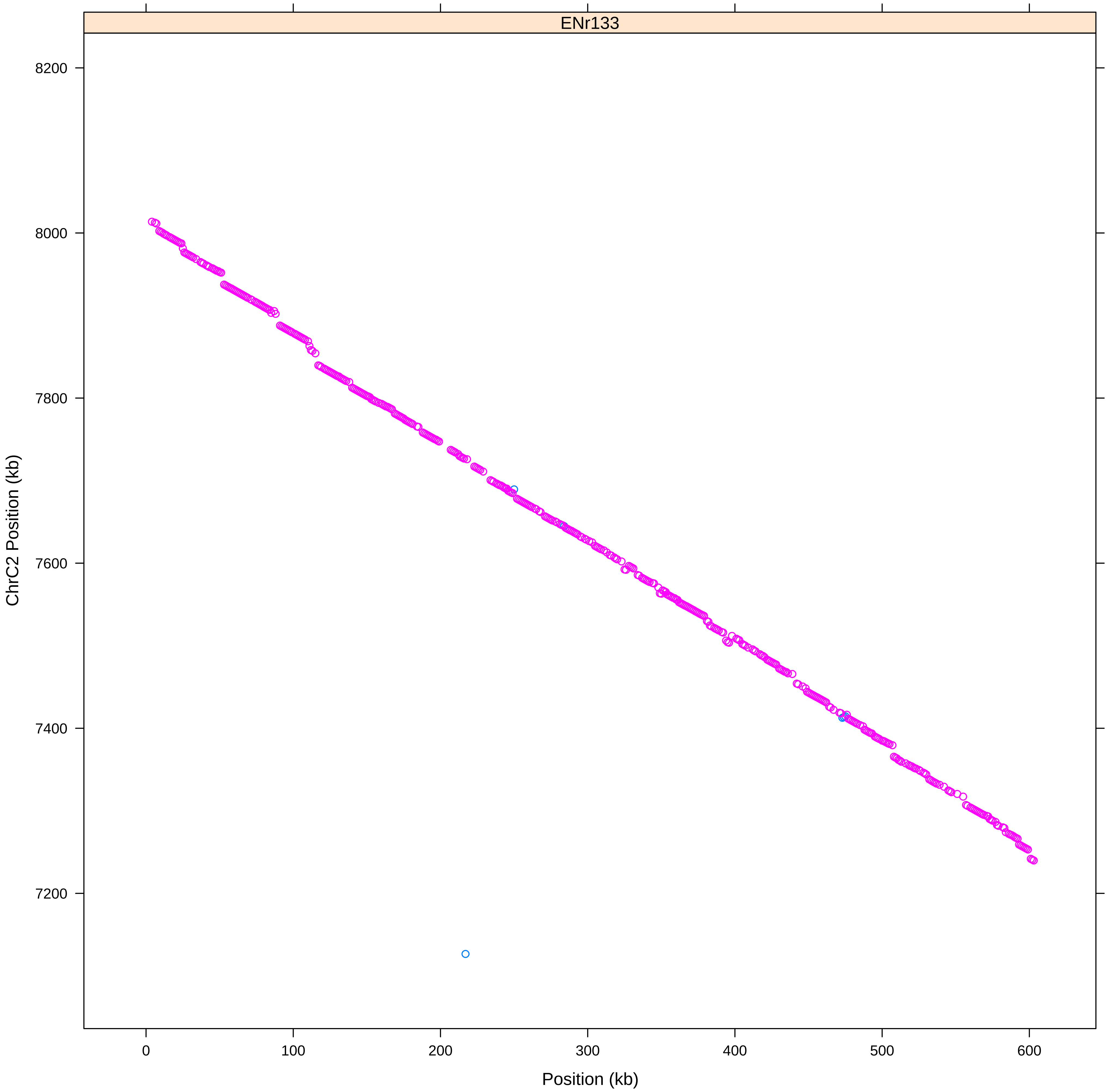

ENr133
8200
8000
7800
7600
7400
7200
ChrC2 Position (kb)
0
100
200
300
400
500
600
Position (kb)

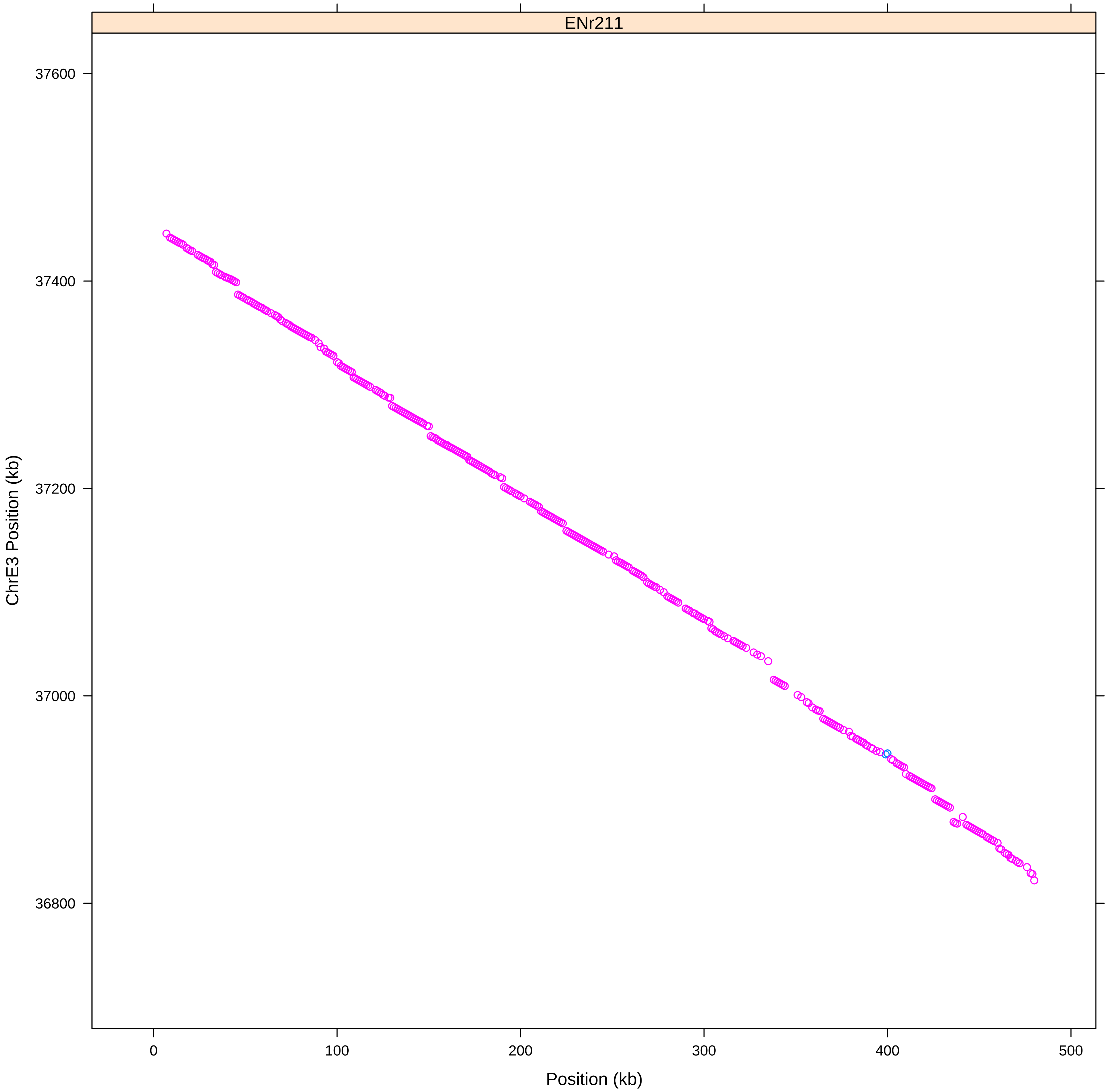

ENr211
ChrE3 Position (kb)
37600
37400
37200
37000
36800
Position (kb)
0
100
200
300
400
500

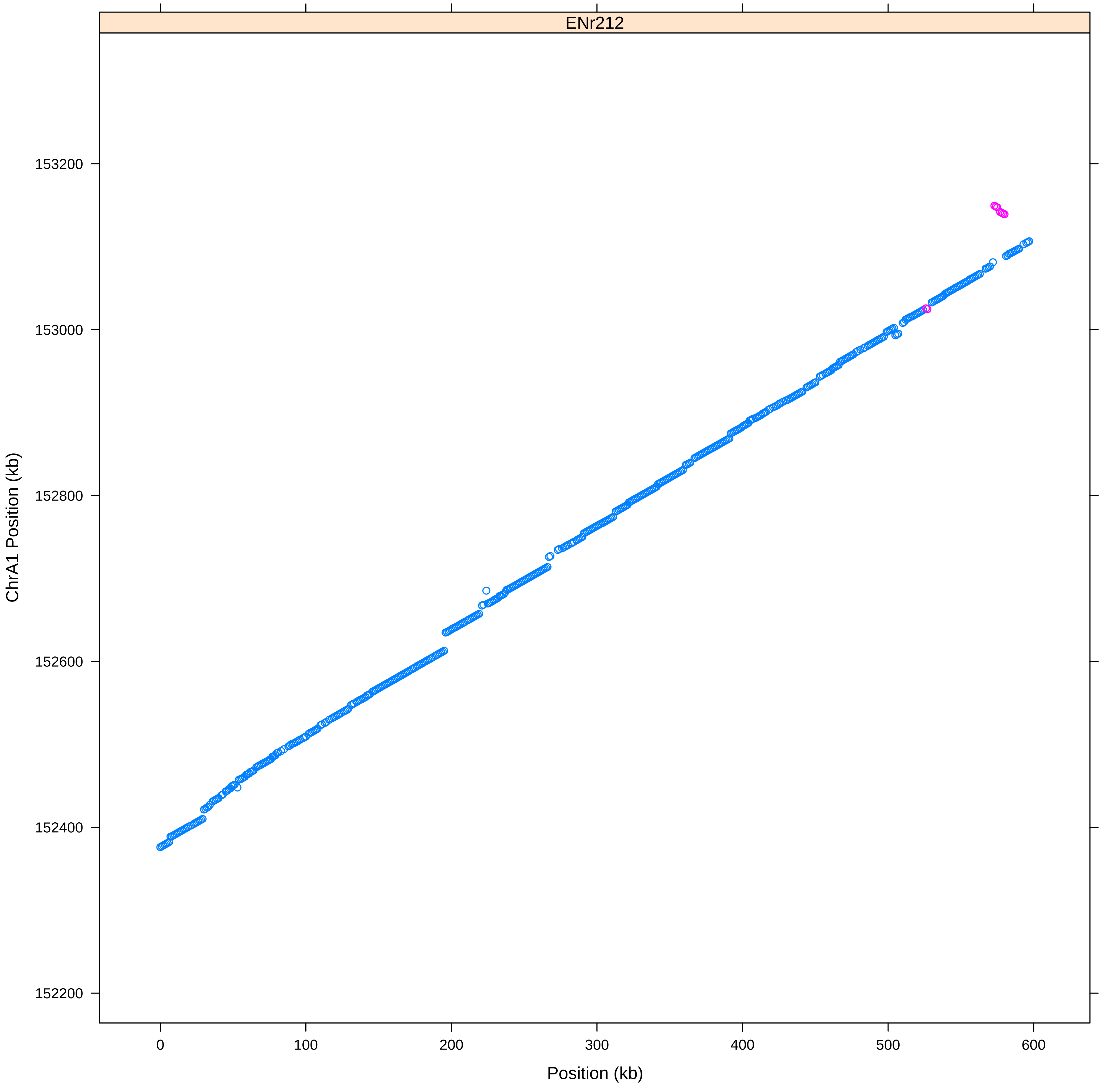

ENr212
ChrA1 Position (kb)
153200
153000
152800
152600
152400
152200
0
100
200
300
400
500
600
Position (kb)

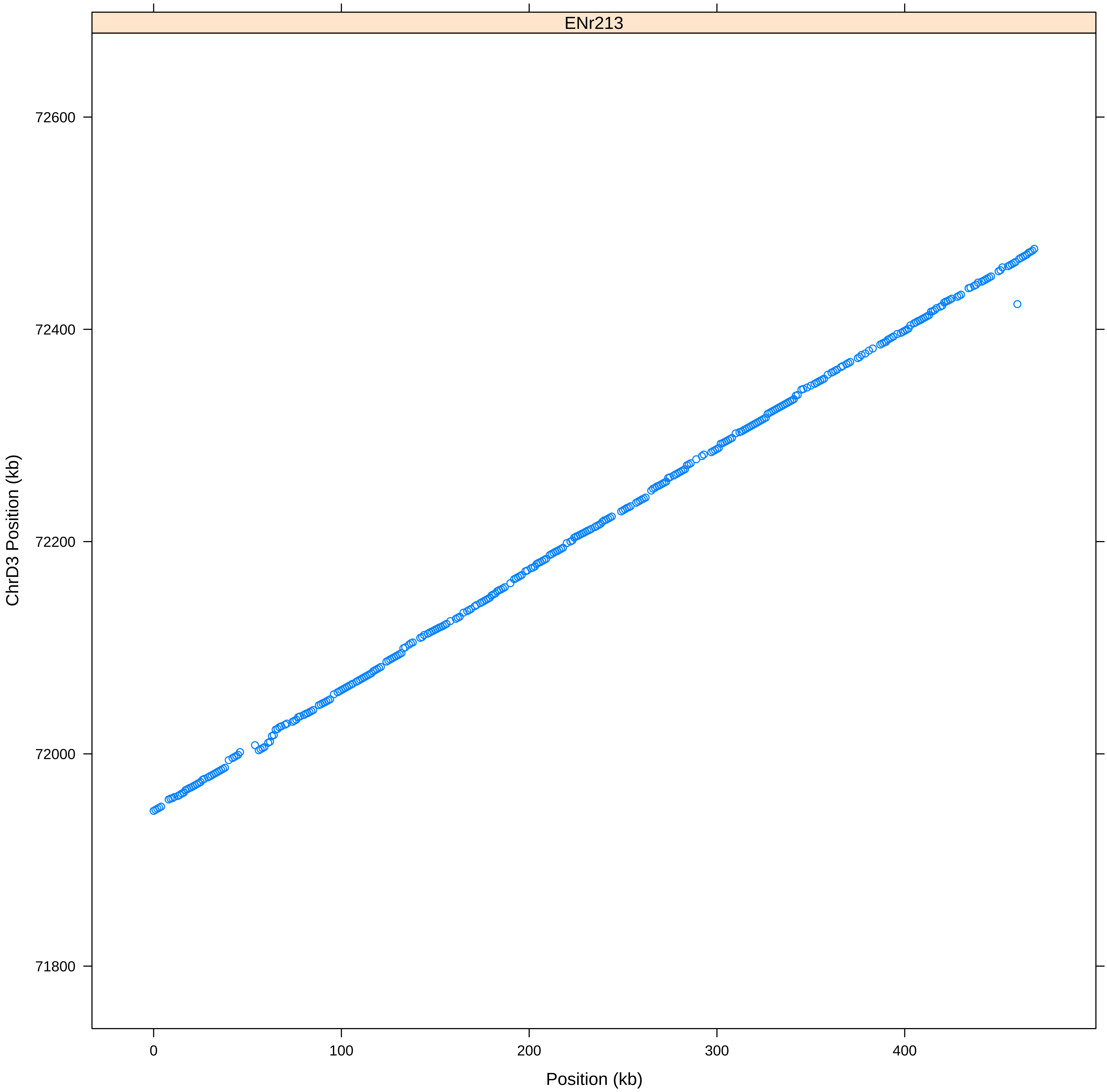

ENr213
72600
72400
72200
72000
71800
ChrD3 Position (kb)
0
100
200
300
400
Position (kb)

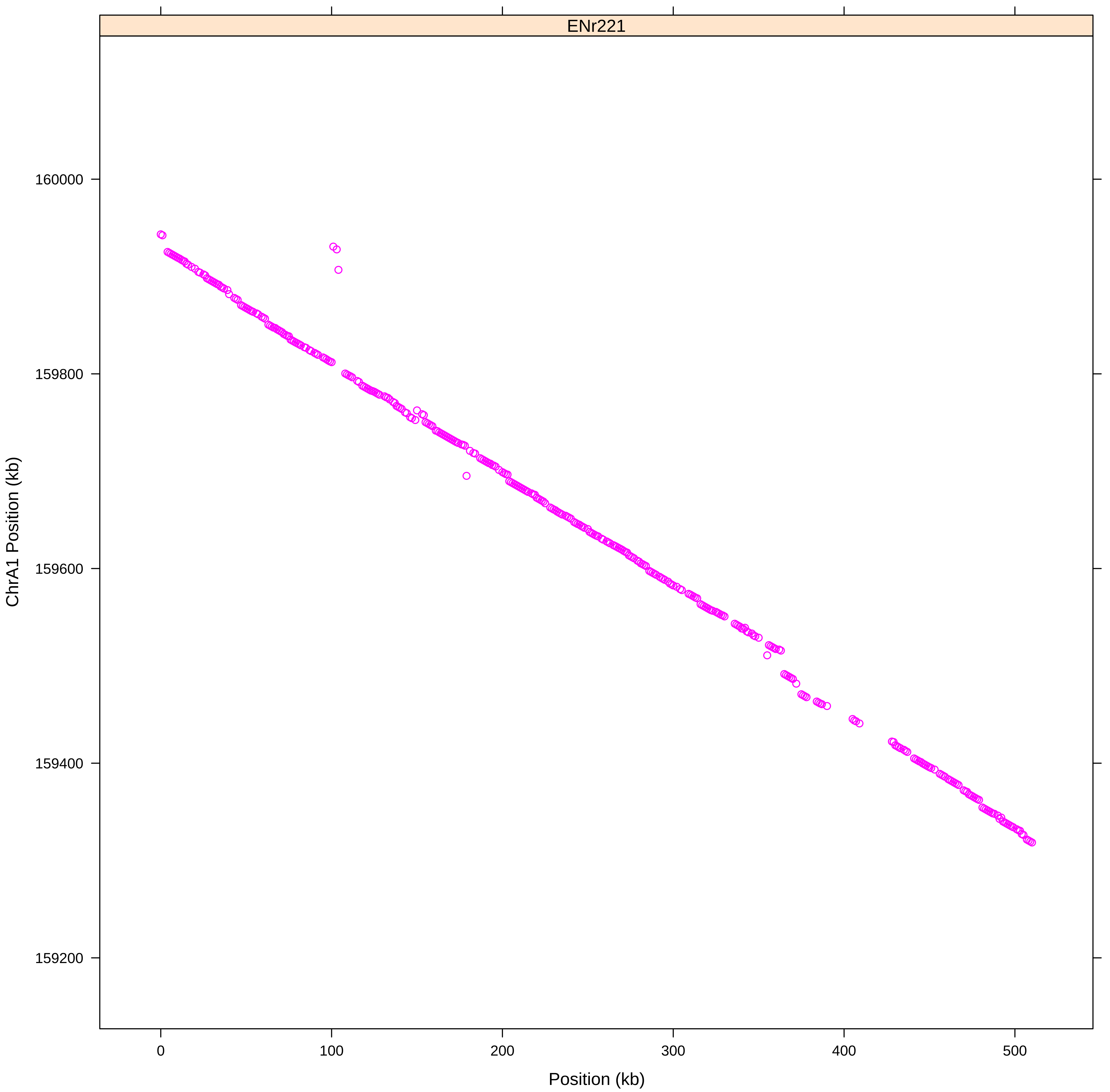
ENr221
ChrA1 Position (kb)
160000
159800
159600
159400
159200
0
100
200
300
400
500
Position (kb)

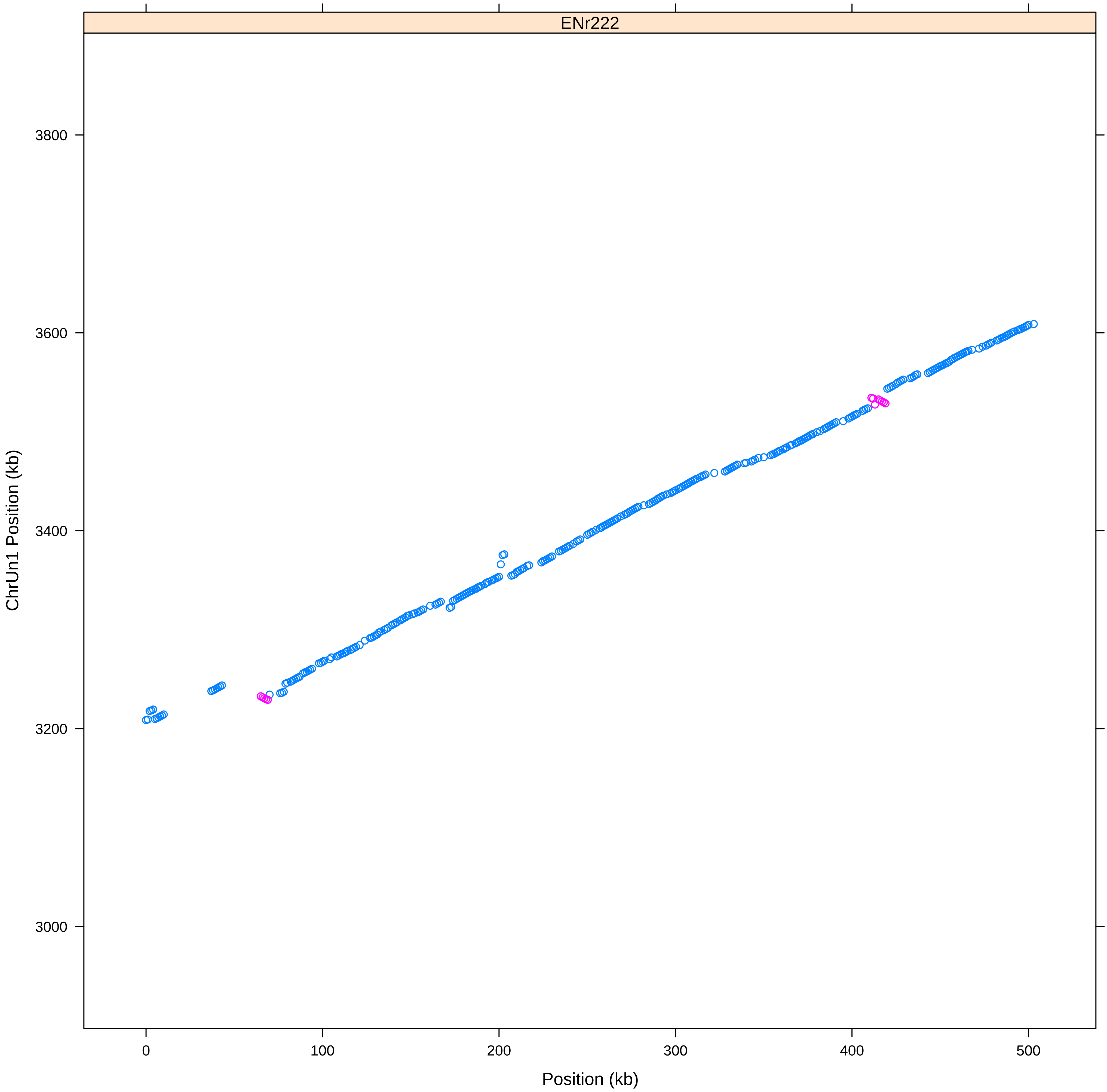

ENr222
ChrUn1 Position (kb)
3000
3200
3400
3600
3800
Position (kb)
0
100
200
300
400
500

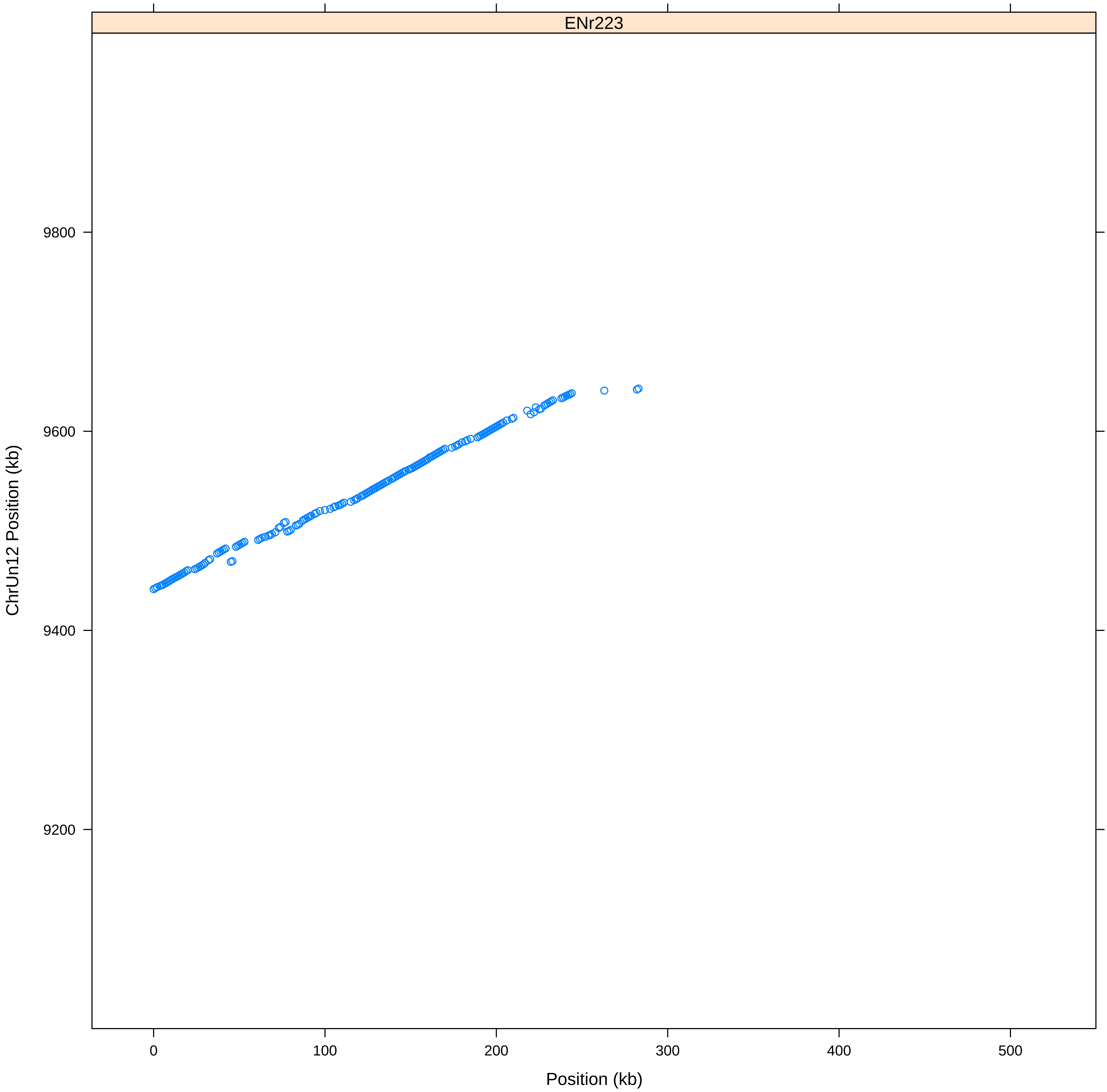

ENr223
9800
9600
9400
9200
ChrUn12 Position (kb)
0
100
200
300
400
500
Position (kb)

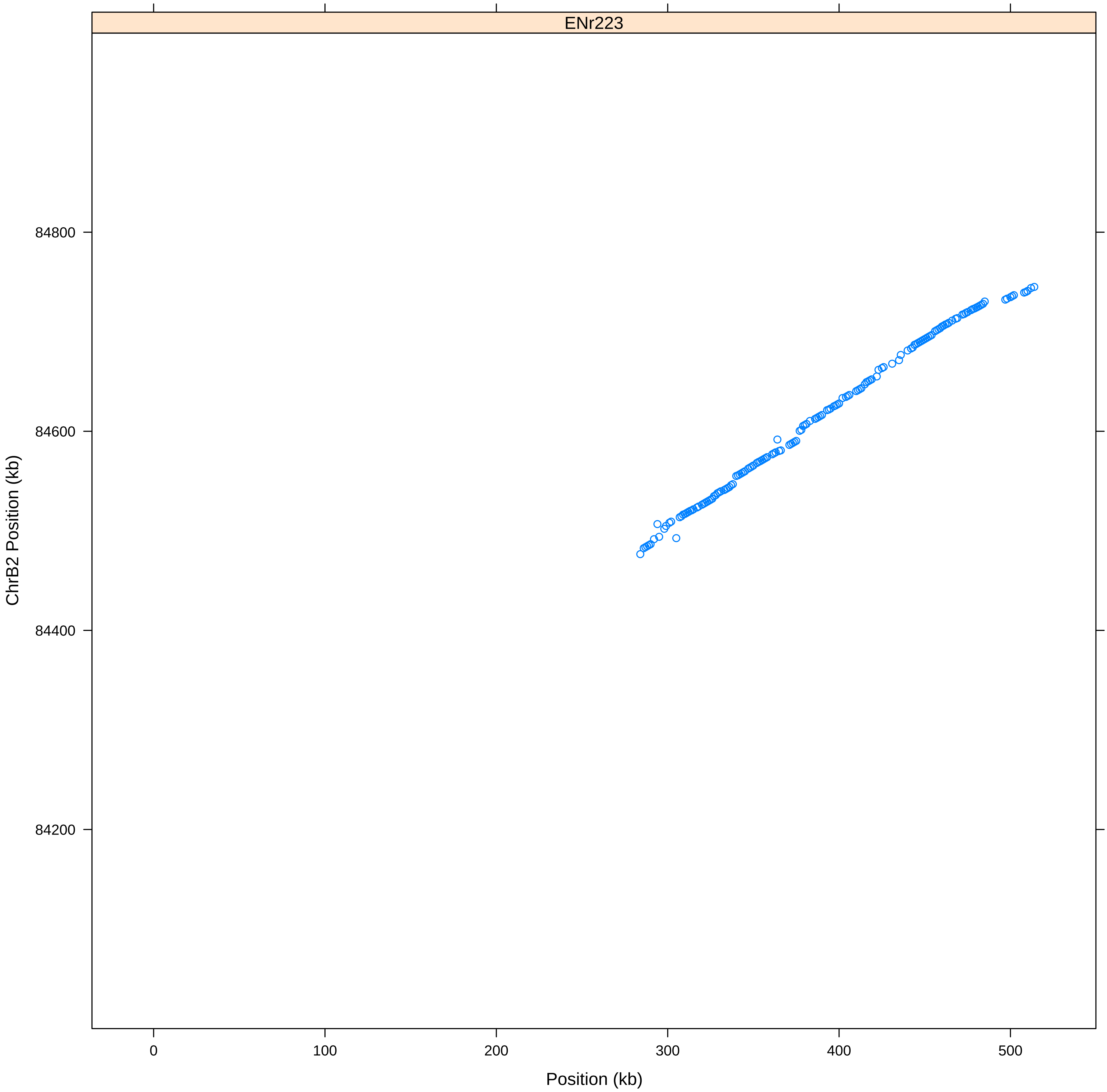
ENr223
ChrB2 Position (kb)
84800
84600
84400
84200
0
100
200
300
400
500
Position (kb)

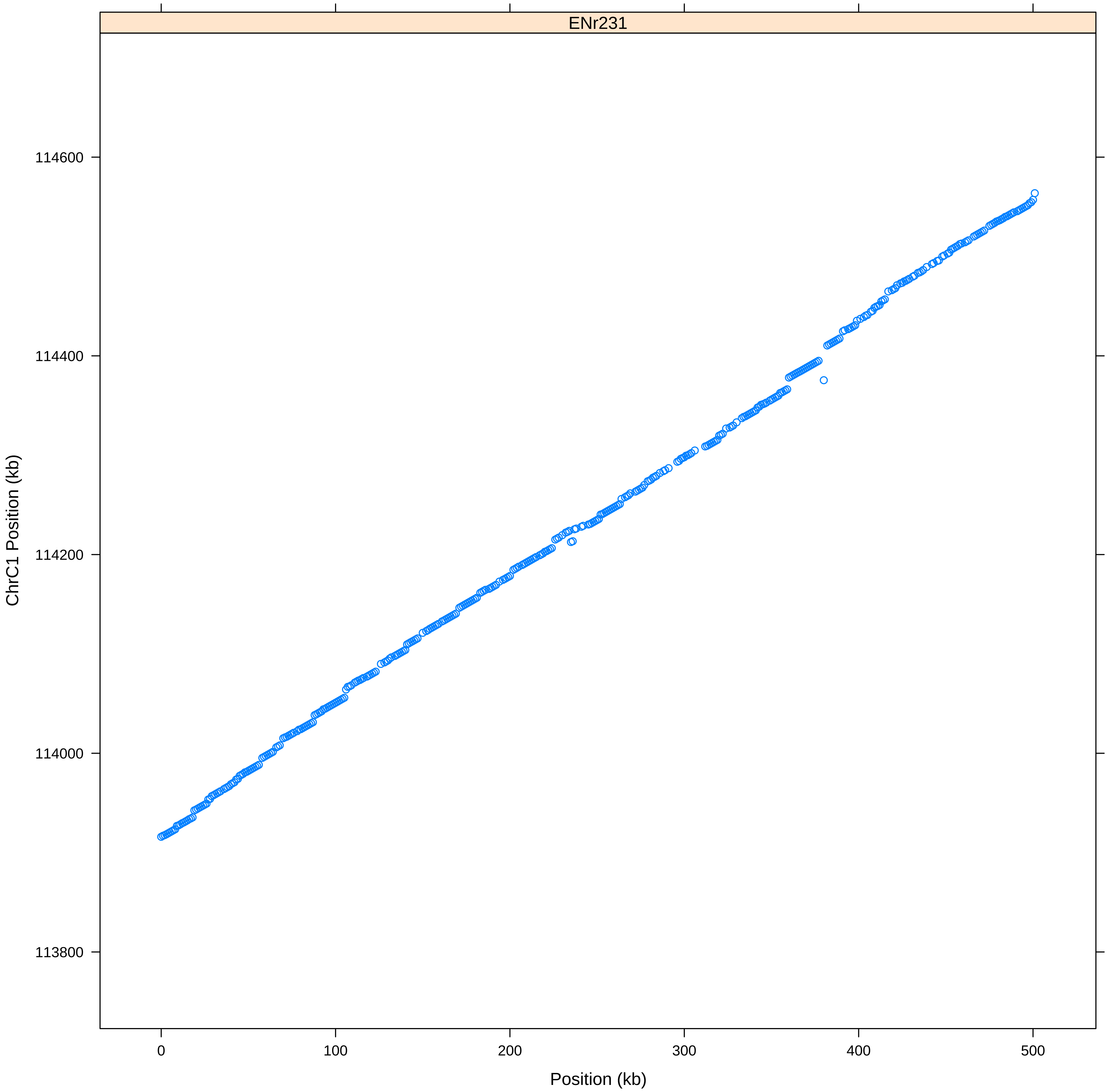
ENr231
ChrC1 Position (kb)
114600
114400
114200
114000
113800
0
100
200
300
400
500
Position (kb)

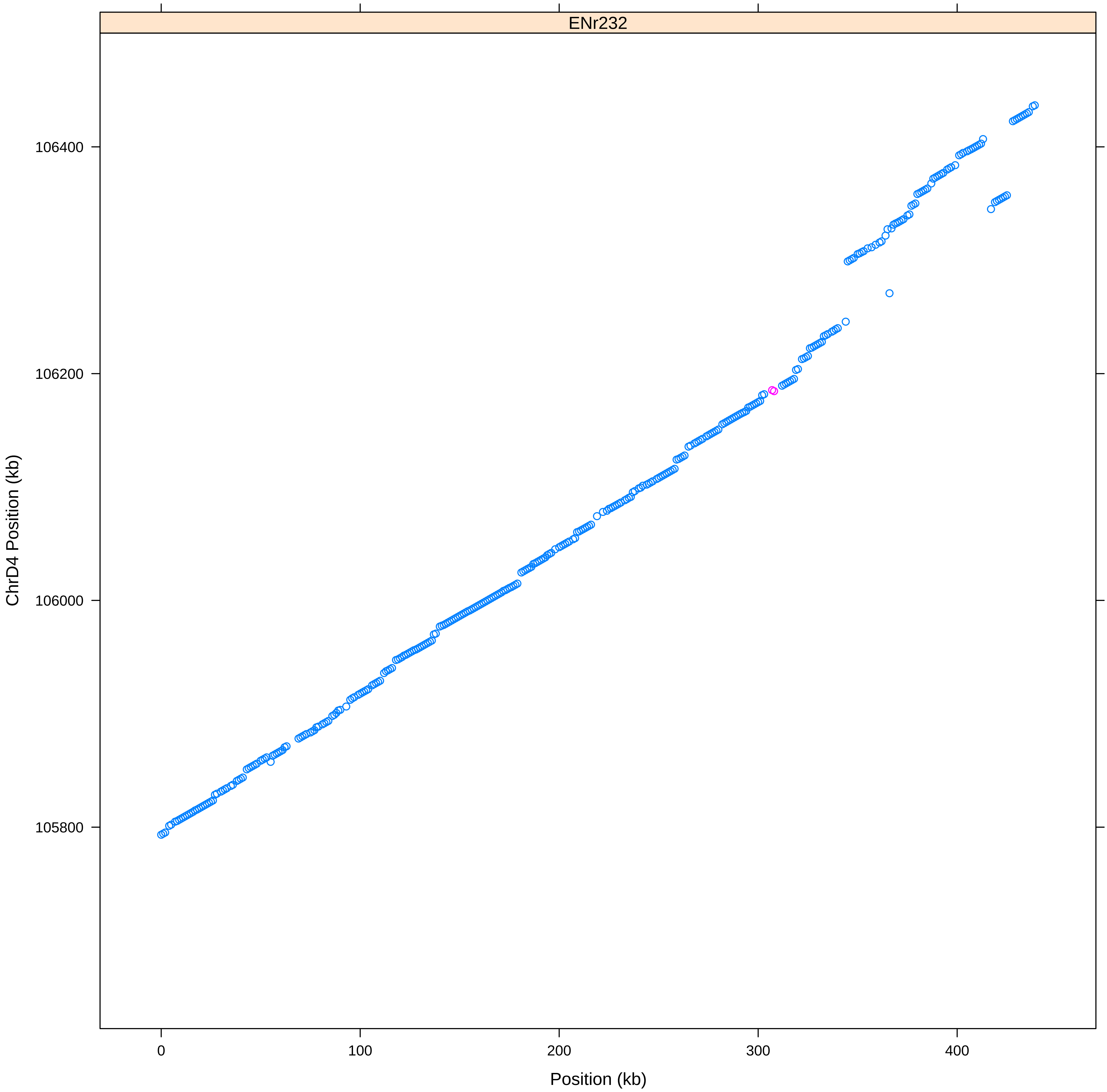
ENr232
ChrD4 Position (kb)
106400
106200
106000
105800
0
100
200
300
400
Position (kb)

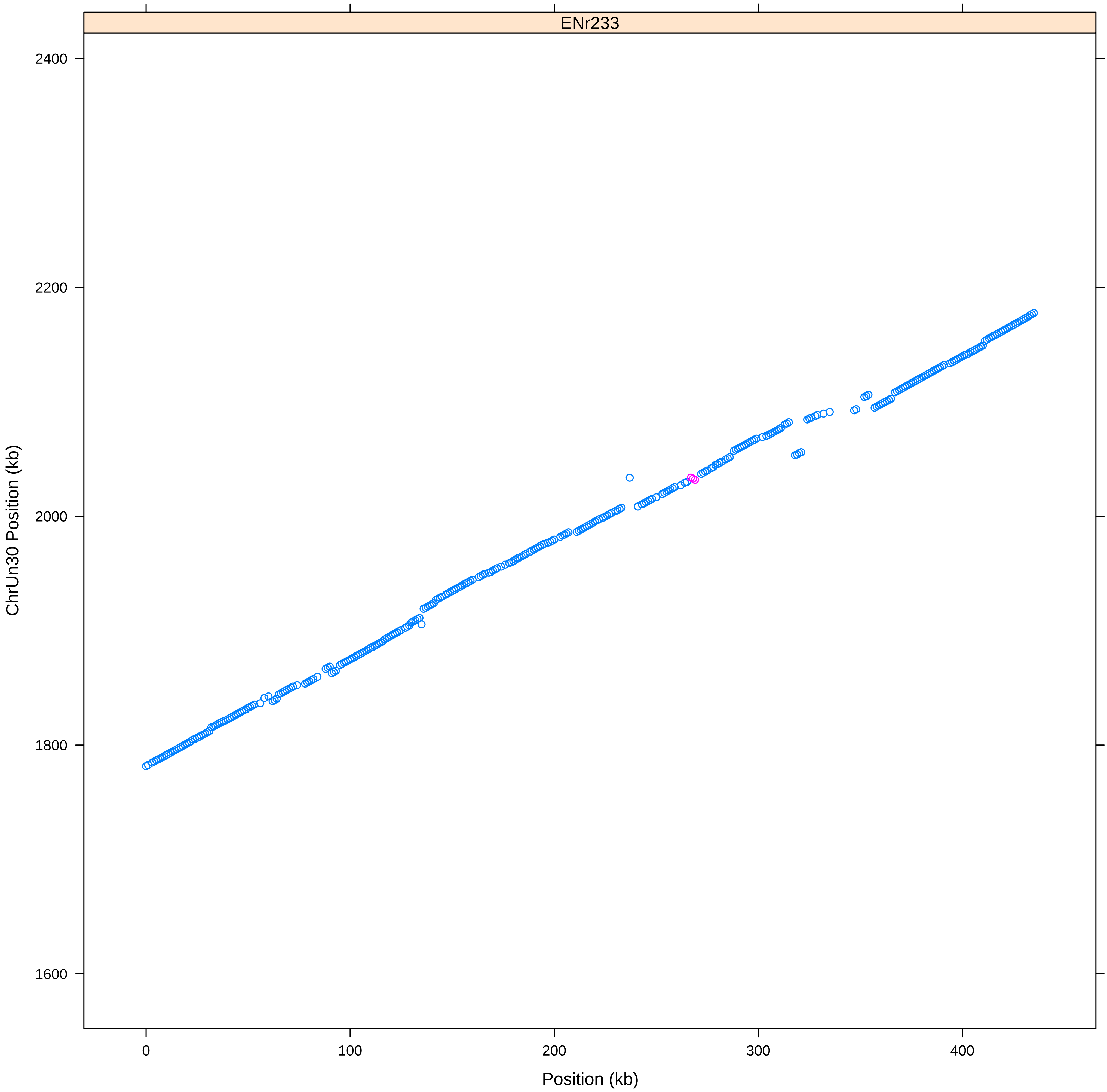
ENr233
ChrUn30 Position (kb)
Position (kb)
1600
1800
2000
2200
2400
0
100
200
300
400

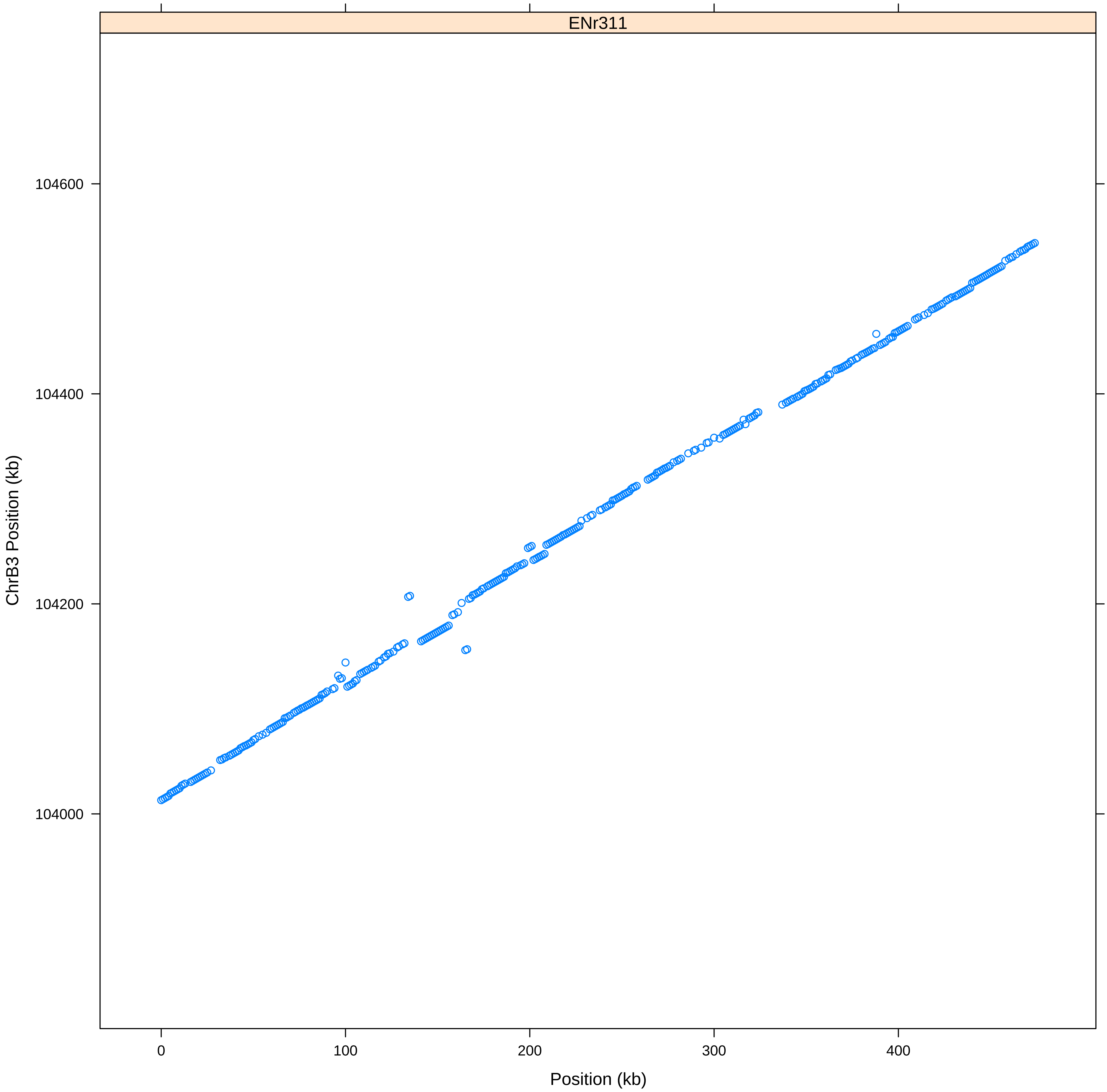
ENr311
ChrB3 Position (kb)
104600
104400
104200
104000
0
100
200
300
400
Position (kb)

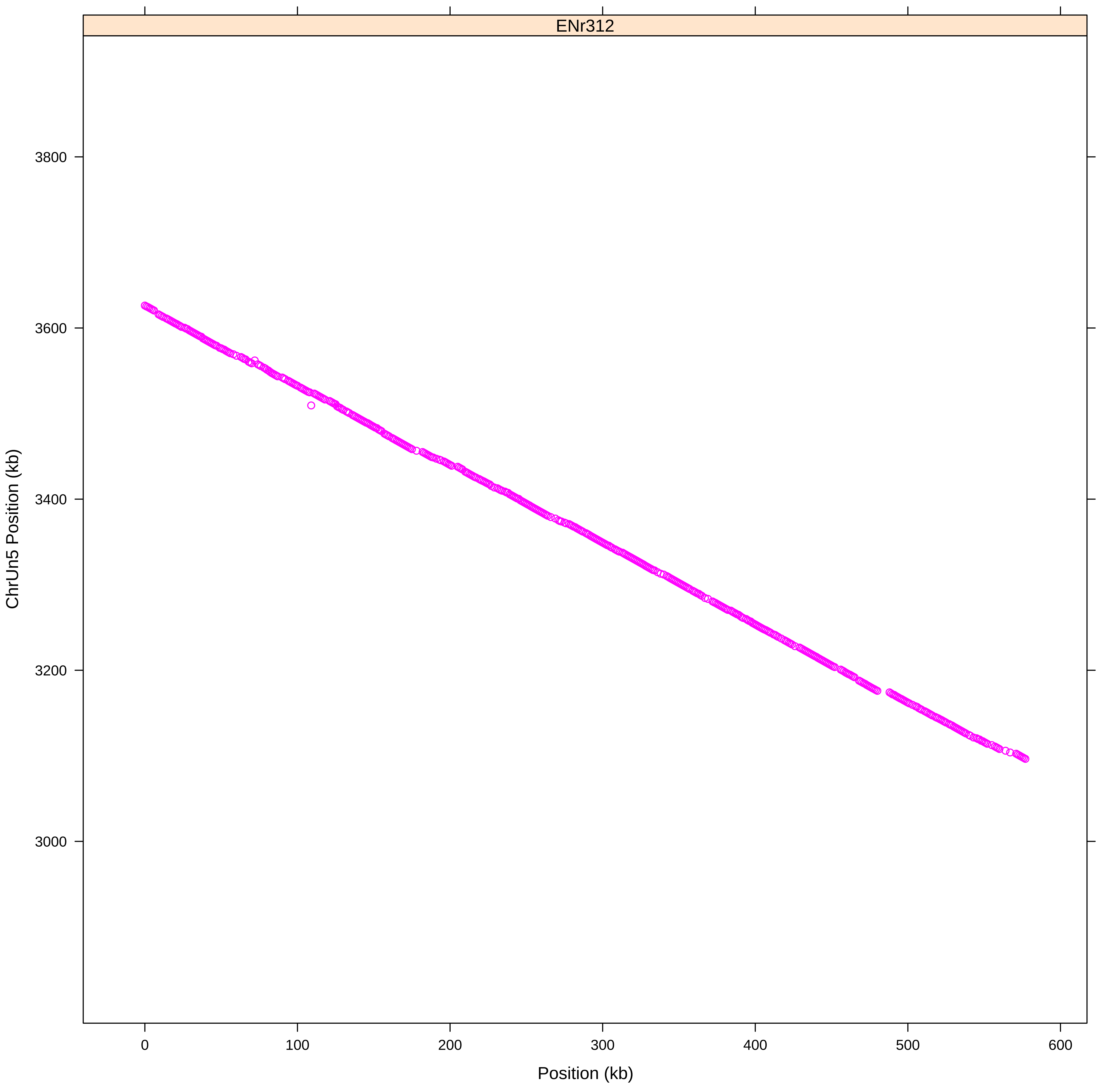
ENr312
ChrUn5 Position (kb)
3000
3200
3400
3600
3800
Position (kb)
0
100
200
300
400
500
600

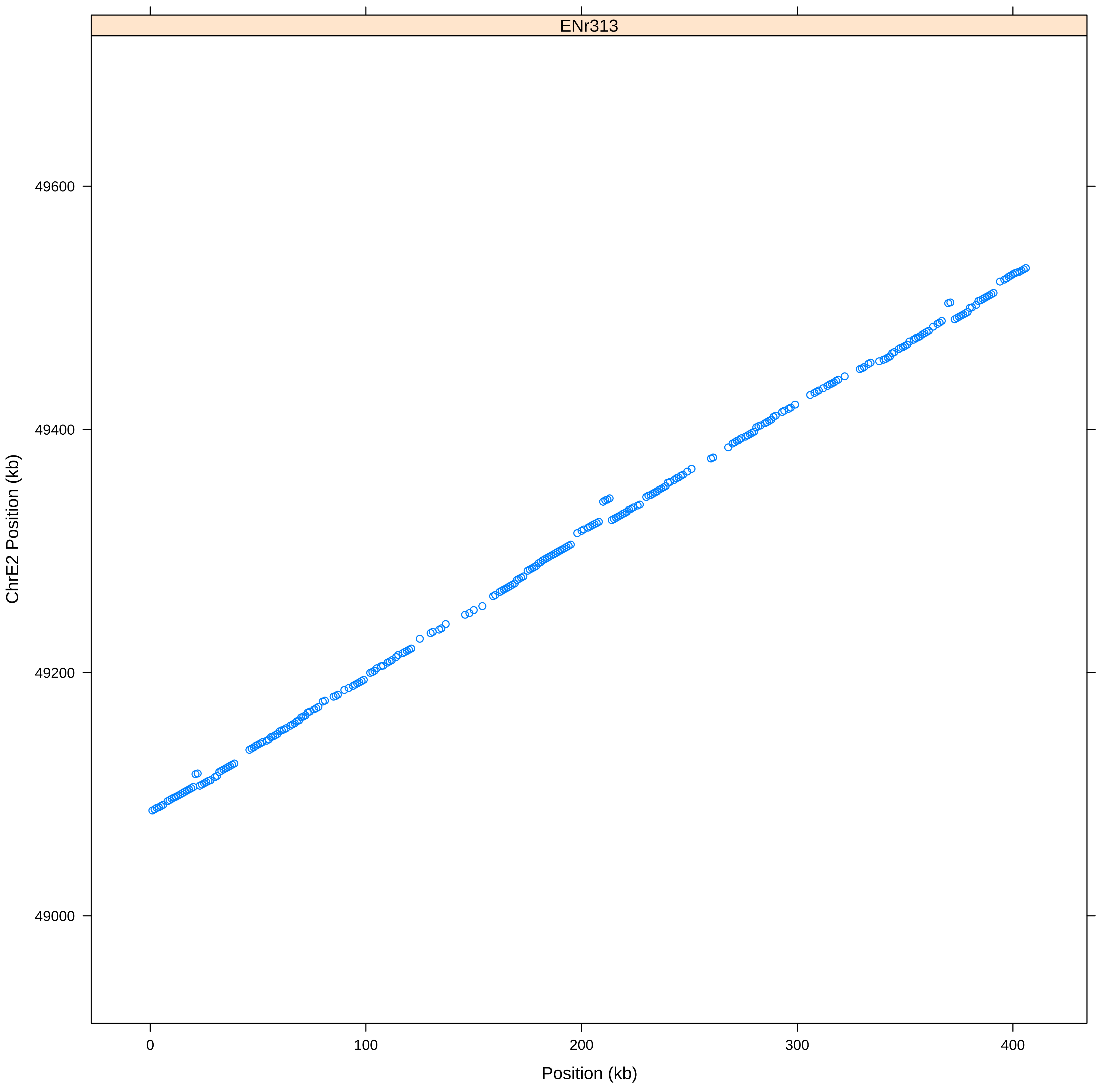
ENr313
ChrE2 Position (kb)
49600
49400
49200
49000
0
100
200
300
400
Position (kb)

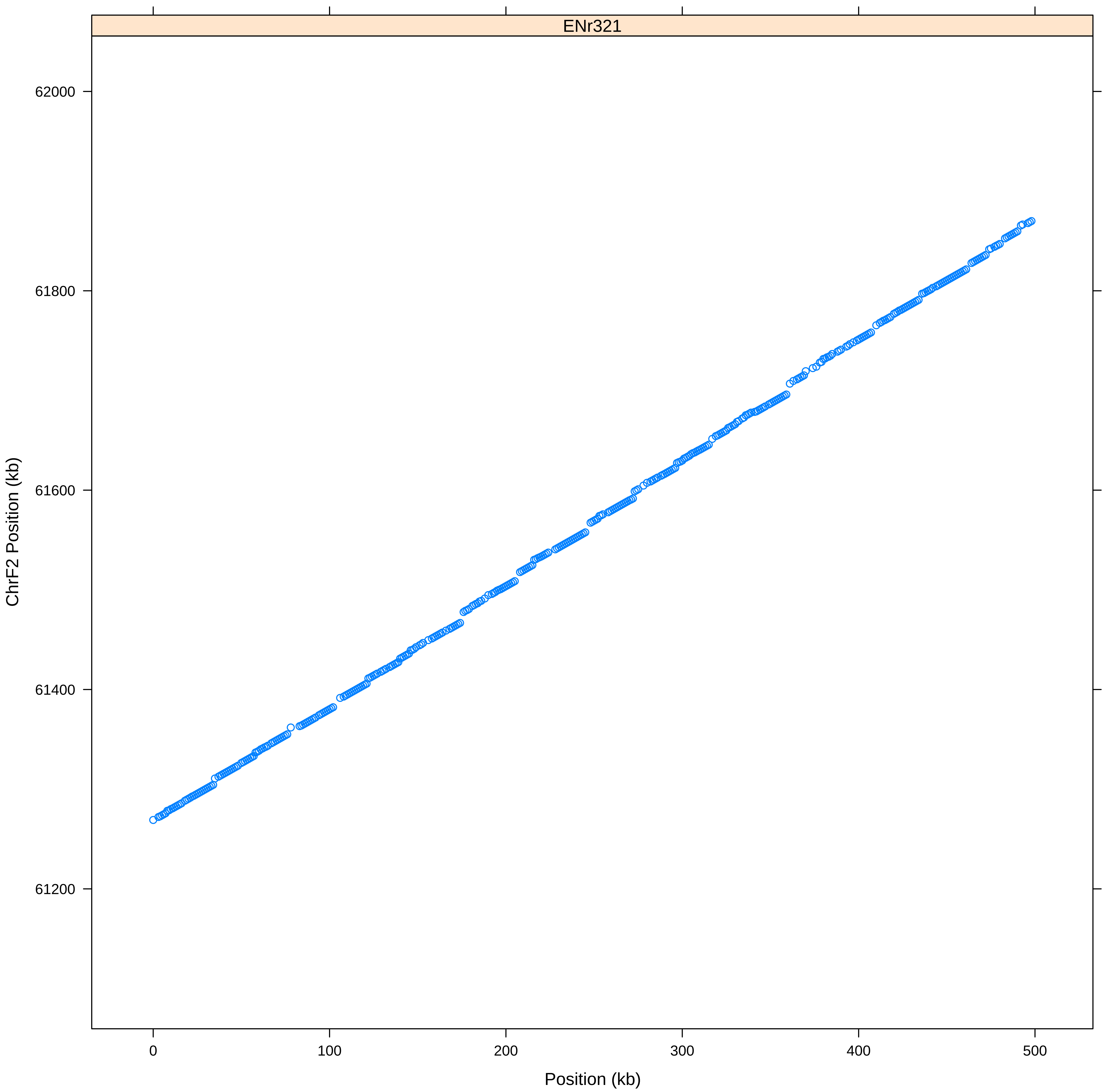
ENr321
ChrF2 Position (kb)
62000
61800
61600
61400
61200
0
100
200
300
400
500
Position (kb)

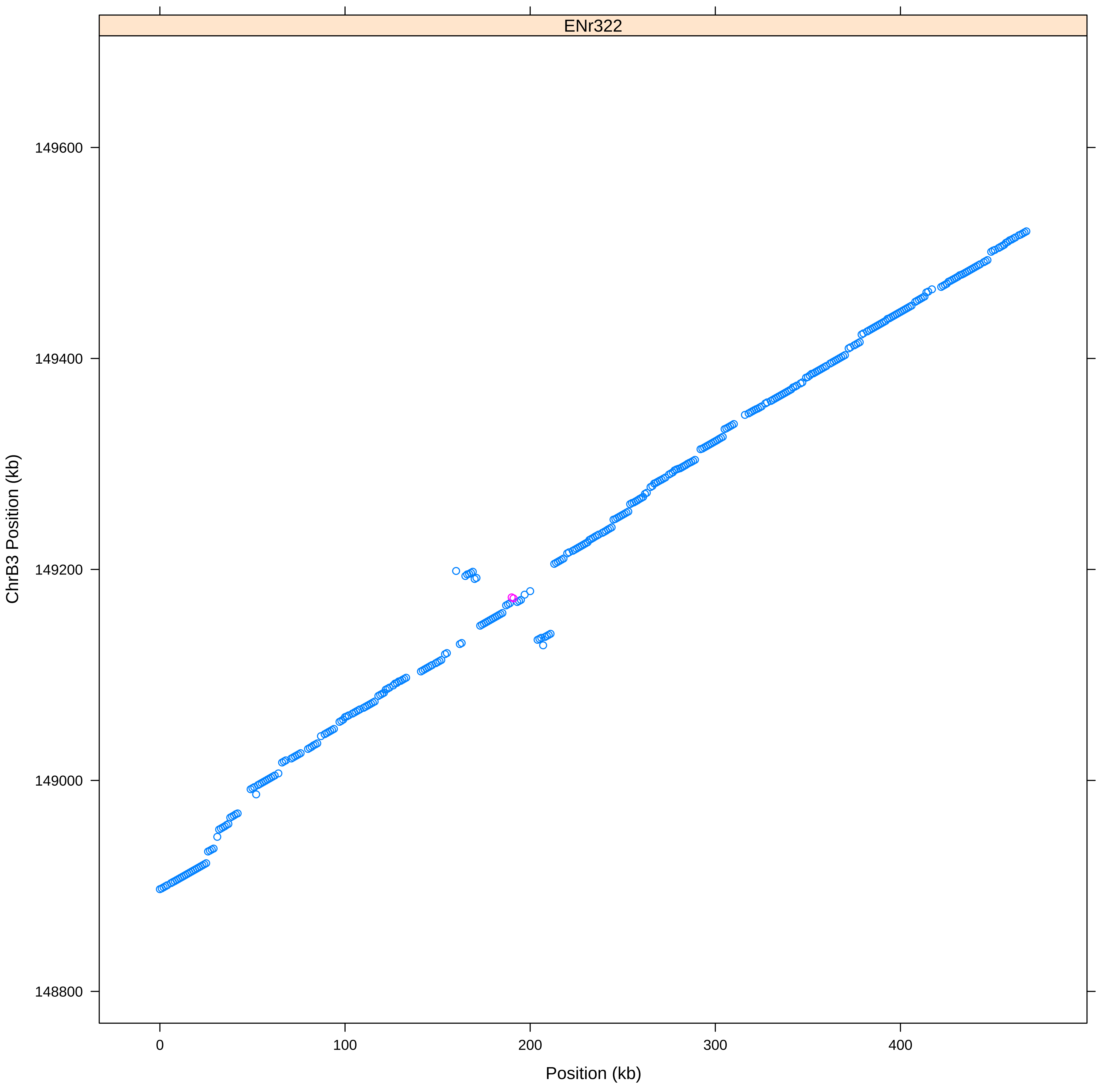
ENr322
ChrB3 Position (kb)
149600
149400
149200
149000
148800
0
100
200
300
400
Position (kb)

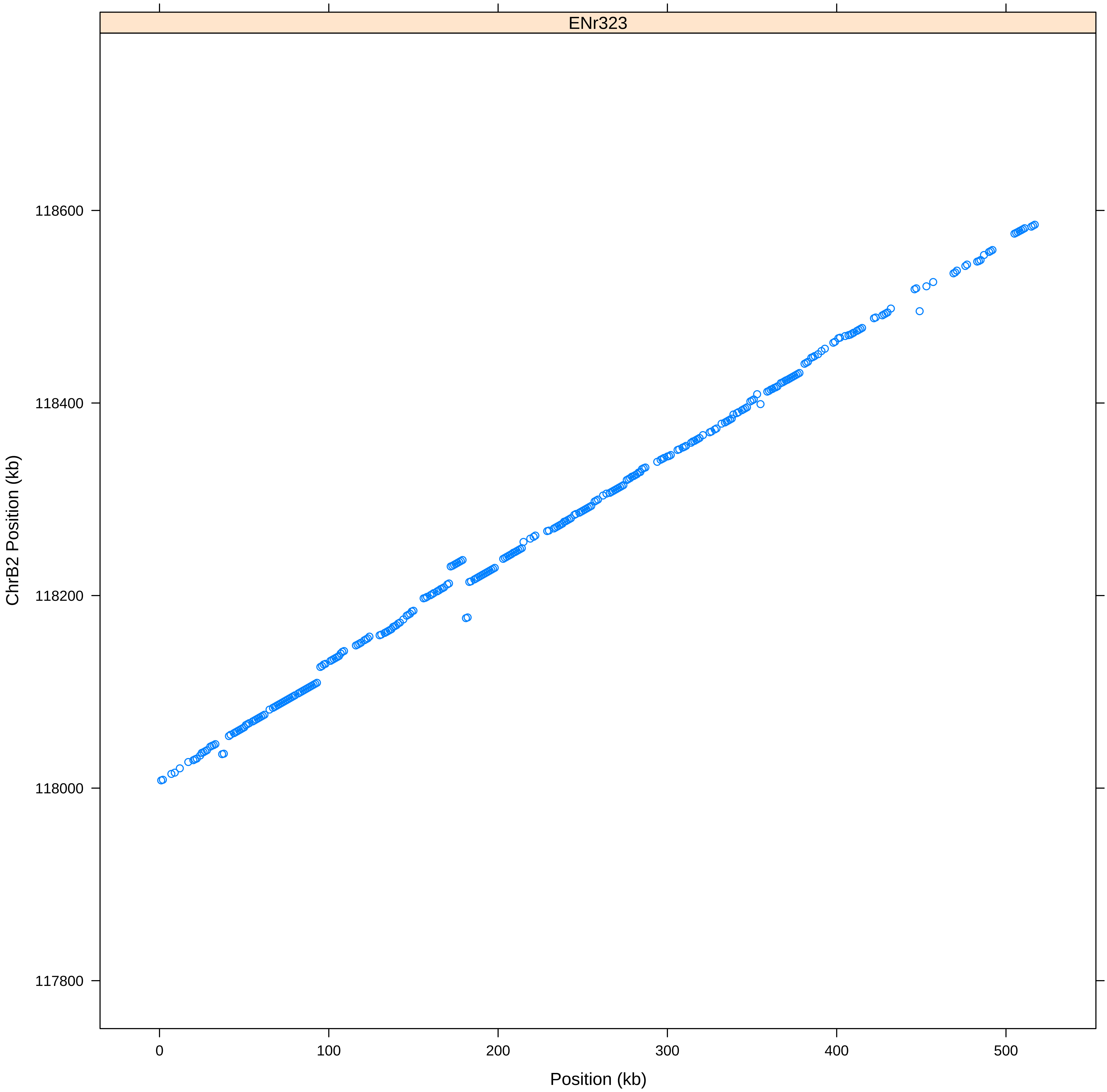
ENr323
ChrB2 Position (kb)
118600
118400
118200
118000
117800
0
100
200
300
400
500
Position (kb)

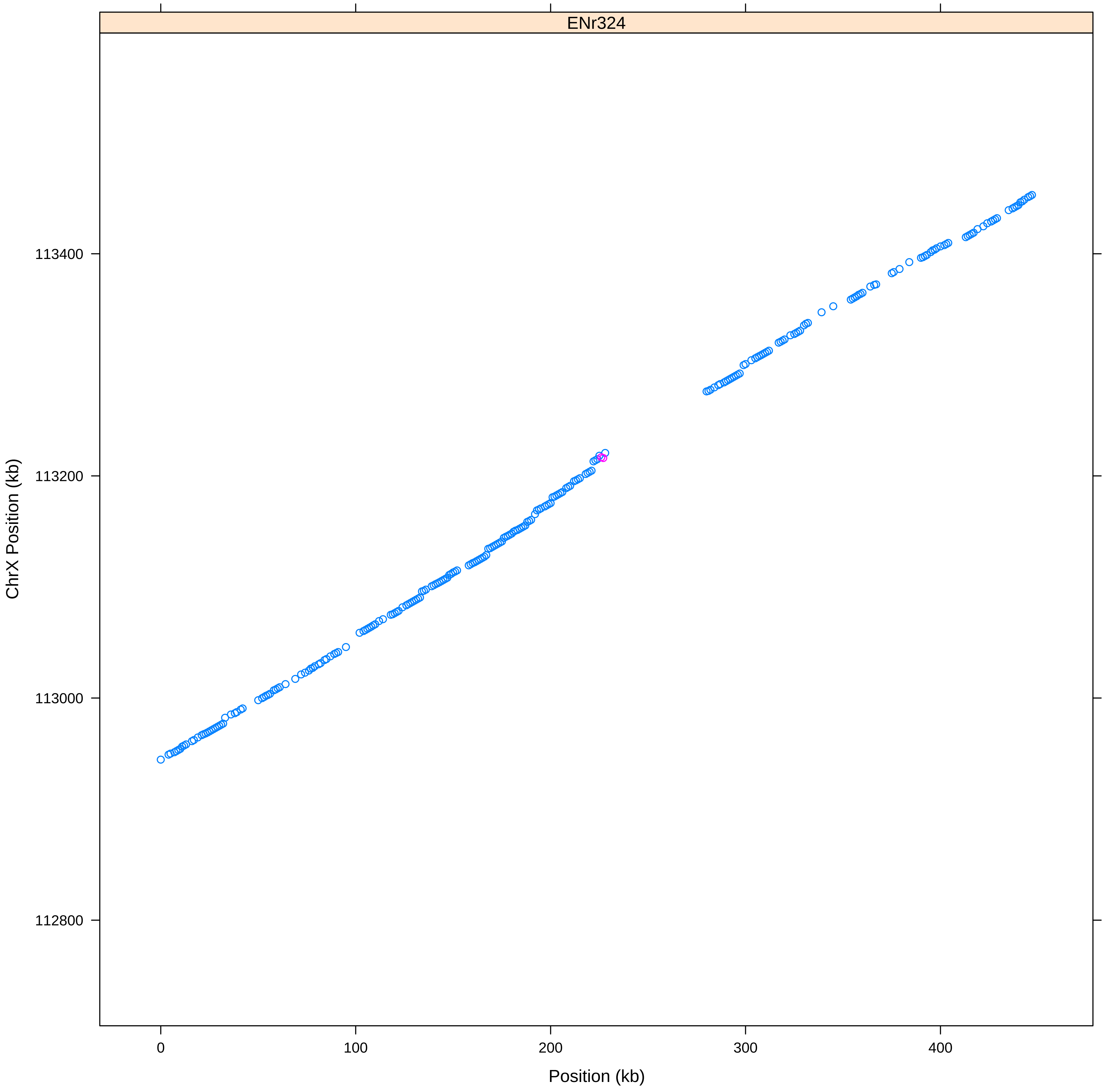

ENr324
ChrX Position (kb)
113400
113200
113000
112800
Position (kb)
0
100
200
300
400

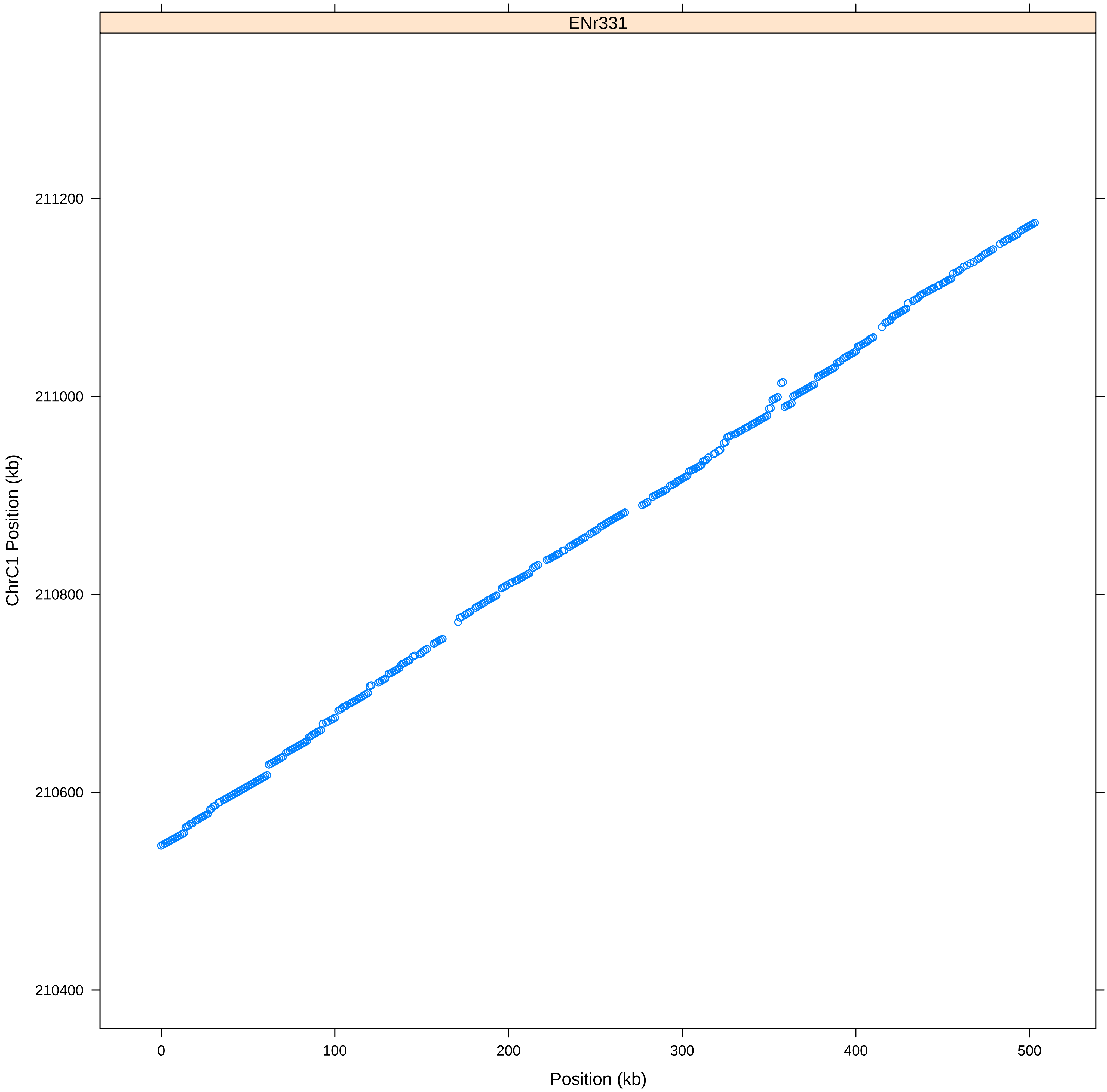

ENr331
ChrC1 Position (kb)
211200
211000
210800
210600
210400
Position (kb)
0
100
200
300
400
500

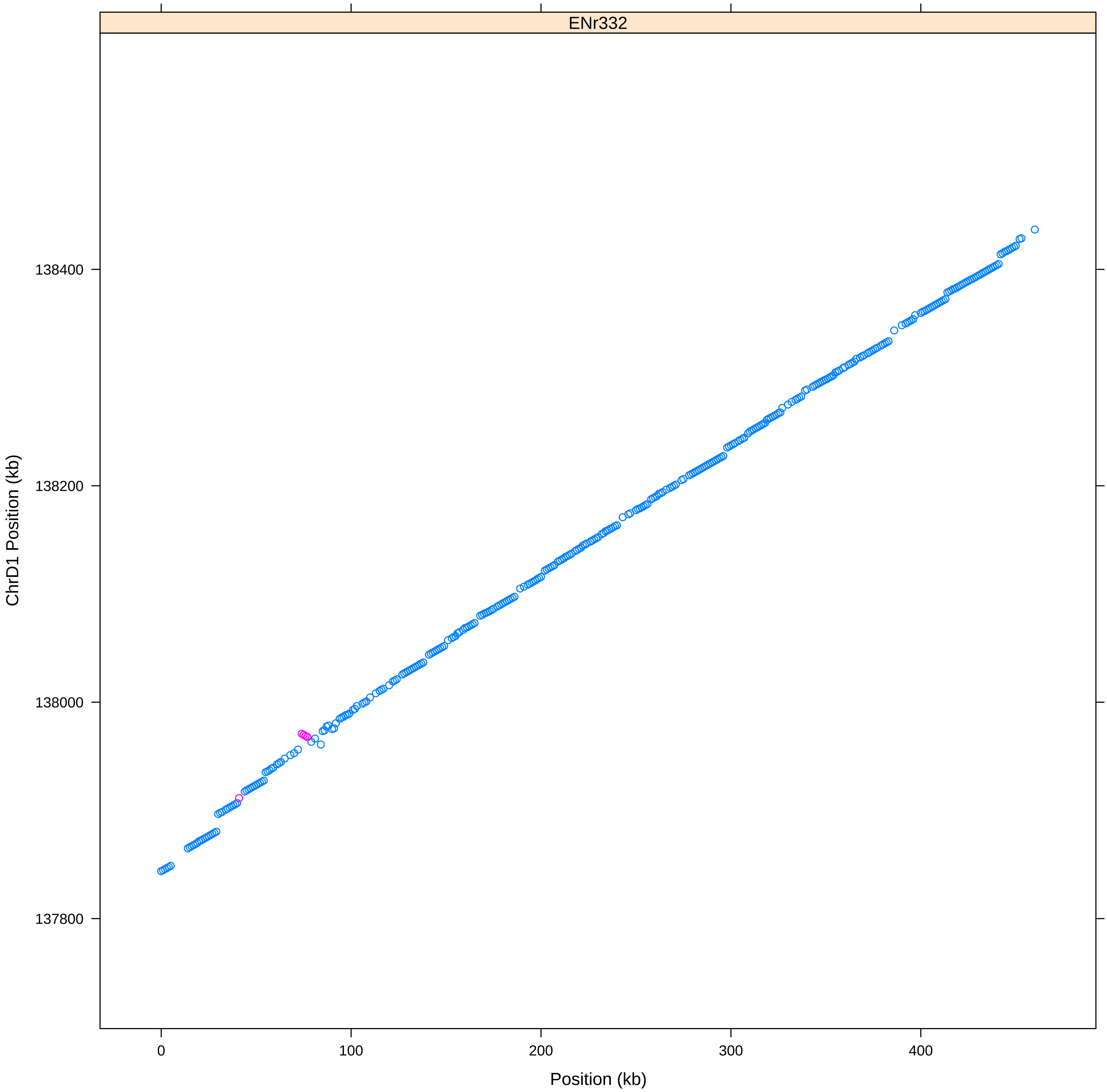
ENr332
ChrD1 Position (kb)
138400
138200
138000
137800
0
100
200
300
400
Position (kb)

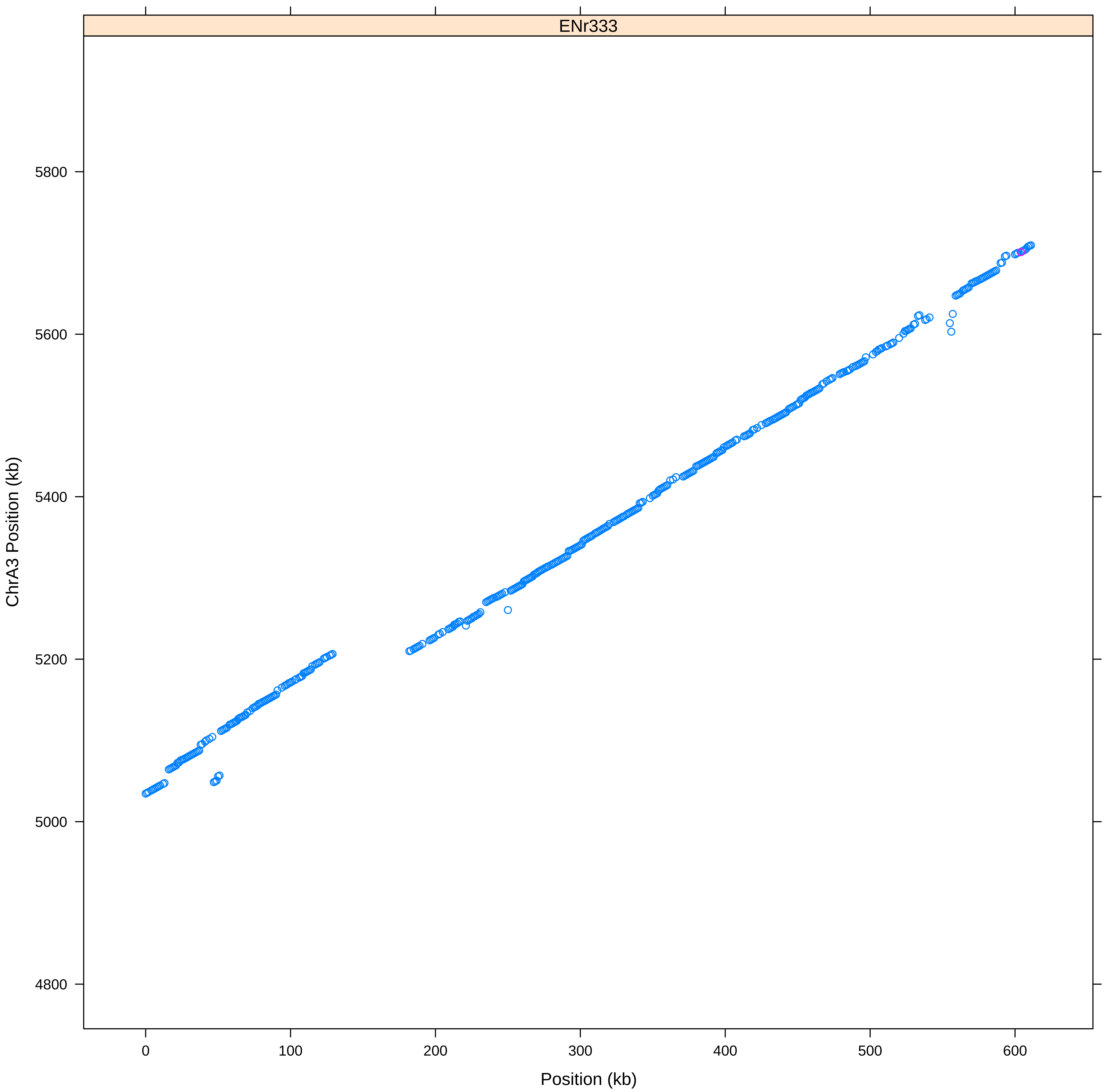

ENr333
ChrA3 Position (kb)
5800
5600
5400
5200
5000
4800
Position (kb)
0
100
200
300
400
500
600

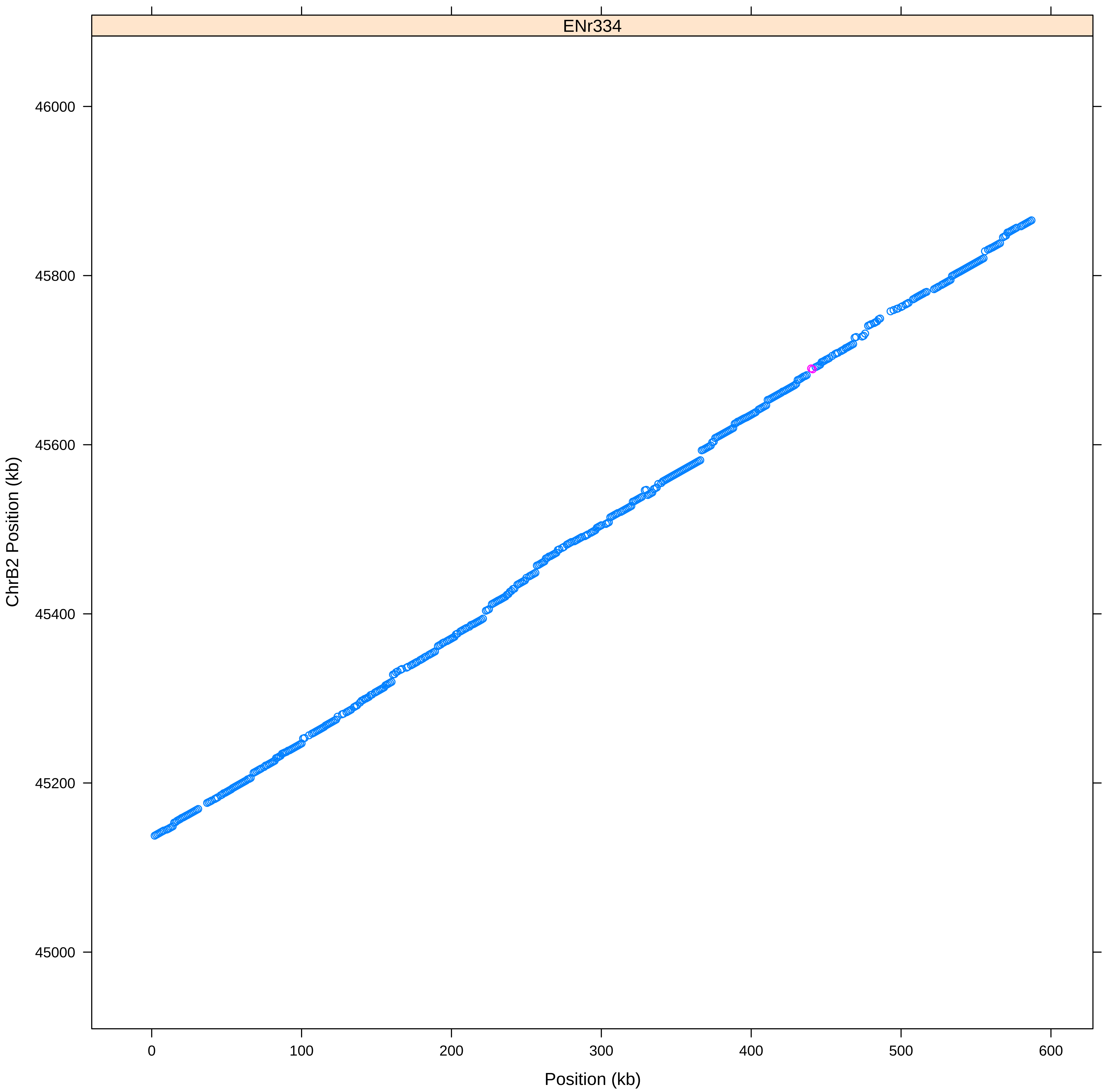

ENr334
ChrB2 Position (kb)
46000
45800
45600
45400
45200
45000
0
100
200
300
400
500
600
Position (kb)
