# Supplementary material for: Light whole genome sequence for SNP discovery across domestic cat breeds
Source: BMC Genomics. 2010 Jun 24;11:406. doi: 10.1186/1471-2164-11-406 (PMC2996934; doi:10.1186/1471-2164-11-406)
Supplement: Additional file 4 — Descriptions of how Additional files 3 and 6 were generated. Methods for generating plots of order and orientation across ENCODE regions and the STRUCTURE analysis of Nancy. [file 1471-2164-11-406-S4.DOC]

*Order and orientation across ENCODE regions*

The order and orientation of sequence along the chromosomes is analyzed in detail by comparing this to ENCODE cat BAC based assemblies generated at NISC. These multi-BAC assemblies (<http://www.nisc.nih.gov/projects/encode/index.cgi?accession_grid=1>) should accurately represent the cat genome within these 500-2,000kb intervals. In Additional file 3, the first page provides an overview of all 44 ENCODE regions. The ENCODE position is on the X axis and the mapped position on a chromosome is on the Y axis, with all chromosomes plotted together in this overview. Each datum represents the positions of an 1,100 base segment of a NISC assembly mapped to a position the WGS assembly, with blue representing forward strand mapping and red representing reverse strand. These 1,100 base segments are taken every 1,000 bases along the NISC assembly, thus overlapping 100 bases on the ends of each segment with the next. The bulk of alignments within each region fall densely along horizontal stretches, and any falling outside are mis-mappings due to either of the following causes: 1) the incorrect placement of a contig within the WGS assembly, or 2) the WGS assembly is only 80% complete, thus a segment in the NISC assembly may not have a corresponding segment in the WGS assembly and gets mapped to some other region of the genome due to some level of similarity. The next 46 pages of Additional file 3 display the ENCODE region and how these 1,100 base segments map at a localized resolution. The ENCODE position is shown on the X axis and the cat chromosome and position on the Y axis. Examining the first region, ENm001, shows a positive sloping series of mapped segments mostly on the forward strand (blue). Four mapped segments are shown in red, indicating local inversions between the two assemblies. Off-diagonal points are local rearrangements, but for ENm001 this is fairly minor. ENm004 spans a chromosome break-point in cat, as well and in other species, e.g. dog and horse, thus shows up on two separate pages in Additional file 3. ENm006 has a fairly odd mapping, but this is because the cat WGS assembly is anchored to large syntenic blocks of the dog genome, thus this represents rearrangements between cat and dog on this fine scale. This will also apply to other regions, like ENm007 and ENm009. ENr223 is split between ChrB2 and ChrUn12. The ChrUn12 portion is not mapped to a cat chromosome, but because the order and orientation of the unmapped syntenic dog chromosome 12 is represented in the assembly this region still represents the segment of the cat genome very well. The other regions show overall good order and orientation given the limitation of a 2.8X WGS assembly.

*Nancy’s placement in STRUCTURE analysis of wild cats*

In order to confirm the subspecies specific affiliation of Nancy, the wildcat used in the current SNP discovery effort, we amplified 18 microsatellite loci in this individual and analyzed the resulting genotype using STRUCTURE. For comparison and verification we included genotypes from over 800 cats typed previously[3] in the STRUCTURE analysis. Included among these 800 individuals are ‘known individuals’ that had been well characterized by mtDNA phylogeny, microsatellite phylogeny, provenance and morphology. These were used as ‘learning samples’ as discussed in the STRUCTURE manual. Additional file 6 Figure S1, shows how the wildcat Nancy in this study clusters with *Felis silvestris cafra*, a southern African wildcat. The cats labeled ‘catus, east’ and ‘catus, west’ are oriental type and European type domestic cats, respectively.
