# Supplementary material for: Light whole genome sequence for SNP discovery across domestic cat breeds
Source: BMC Genomics. 2010 Jun 24;11:406. doi: 10.1186/1471-2164-11-406 (PMC2996934; doi:10.1186/1471-2164-11-406)
Supplement: Additional file 5 — PCR validation results for 94 variants. Table S3 lists the variants by position on the genome assembly, which alleles are expected, and the alleles observed across 8 cats. Pink colored cells indicate the cat(s) from which the alternate allele was discovered in the light whole genome sequence. [file 1471-2164-11-406-S5.DOCX]

Table S3. Validation results from PCR resequencing of 94 variants. Pink coloring indicates the cat(s) with the detected WGS alternate allele relative to the reference allele, thus the alternate allele should be observed in this cat. Three variants required sequencing in Cinnamon because all other cats were homozygous for the alternate allele.

| SNP | Reference | Alternate | Nancy | Pixel | Scooter | Speedy | Tipper | Wishbone | Zeelie | Cinnamon |
| --- | --- | --- | --- | --- | --- | --- | --- | --- | --- | --- |
| chrC1~36864248 | C | G | GG | GG | GG | GG | GG | GG | GG | CG |
| chrD3~44560197 | T | C | CC | CC | CC | CC | CC | CC | CC | CT |
| chrA3~106100570 | G | A | GG | GG | GG | AG | GG | GG | GG |  |
| chrB1~187255897 | G | A | GG | GG | GG | AG | GG | GG | GG |  |
| chrA2~115418161 | G | A | AG | GG | GG | GG | GG | GG | GG |  |
| chrA3~107937682 | G | A | AG | GG | GG | GG | GG | GG | GG |  |
| chrA3~143924685 | G | A | AG | GG | GG | GG | GG | GG | GG |  |
| chrB1~204978075 | G | A | AG | GG | GG | GG | GG | GG | GG |  |
| chrB3~25736392 | C | T | CT | CC | CC | CC | CC | CC | CC |  |
| chrD2~83141120 | G | A | AG | GG | GG | GG | GG | GG | GG |  |
| chrD4~416493 | G | C | CG | GG | GG | GG | GG | GG | GG |  |
| chrD4~56606863 | C | T | CT | CC | CC | CC | CC | CC | CC |  |
| chrE2~16543906 | T | C | CT | TT | TT | TT | TT | TT | TT |  |
| chrC1~208070336 | A | G | GG | GG | GG | GG | GG | GG | GG | AA |
| chrD3~49180835 | G | A | GG | AA | AA | AA | AA | AA | AA |  |
| chrUn6~868199 | G | A | GG | AA | GG | GG | GG | GG | GG |  |
| chrX~135802116 | C | T | CC | TT | CC | CC | CC | CC | CC |  |
| chrB3~125638554 | A | C | CC | AA | AA | AA | AA | AA | AA |  |
| chrB3~145889507 | C | T | TT | CC | CC | CC | CC | CC | CC |  |
| chrC2~50836871 | C | T | TT | CC | CC | CC | CC | CC | CC |  |
| chrD4~38154893 | G | C | CC | GG | GG | GG | GG | GG | GG |  |
| chrUn11~5844851 | C | G | CC | GG | GG | GG | GG | GG | NN |  |
| chrC1~129121324 | G | T | TT | NN | GG | GG | GG | GG | GG |  |
| chrUn1~14250149 | T | C | CC | TT | TT | TT | TT | NN | TT |  |
| chrA1~89494949 | A | G | GG | GG | GG | GG | GG | AG | GG |  |
| chrB1~1507353 | A | C | CC | CC | CC | CC | CC | AC | CC |  |
| chrE2~14514077 | A | C | CC | CC | CC | CC | AC | CC | CC |  |
| chrA1~269358170 | T | C | CC | CC | CC | CC | CT | CC | CC |  |
| chrB4~163434211 | A | G | GG | GG | GG | GG | GG | AG | GG |  |
| chrC1~208913296 | A | G | GG | AG | GG | GG | GG | GG | GG |  |
| chrC1~3866633 | G | C | CC | CC | CC | CG | CC | CC | CC |  |
| chrA2~19469820 | T | C | CC | CC | CC | CC | CC | CC | CT |  |
| chrB3~145164093 | T | C | CC | CC | CC | CC | CT | CC | CT |  |
| chrE1~135394184 | T | G | TT | TT | GT | TT | TT | TT | GT |  |
| chrE2~68958526 | C | T | TT | CT | TT | CT | TT | TT | TT |  |
| chrUn7~2002153 | C | A | CC | AC | AC | CC | CC | CC | CC |  |
| chrA2~127737209 | T | C | CC | CT | CC | CC | CT | CC | CC |  |
| chrA2~66584371 | C | T | CT | CC | CC | CT | CC | CC | CC |  |
| chrB2~24503516 | C | G | GG | GG | GG | GG | NN | CC | GG |  |
| chrD4~5616905 | A | T | TT | AA | AA | AA | AA | AA | AT |  |
| chrA3~28530346 | C | T | CC | CT | TT | TT | TT | TT | TT |  |
| chrD1~82115961 | G | A | AA | GG | AG | GG | GG | GG | GG |  |
| chrD3~77591434 | C | T | CT | TT | TT | TT | TT | CC | TT |  |
| chrD2~112309333 | G | A | AG | GG | GG | GG | AA | GG | GG |  |
| chrD1~121432454 | G | A | GG | AA | GG | GG | AA | GG | GG |  |
| chrD4~70867821 | T | C | CC | TT | CC | CC | TT | CC | CC |  |
| chrD2~80566932 | G | A | AA | NN | AA | AA | GG | GG | AA |  |
| chrC1~50815813 | G | A | AG | AG | GG | GG | AG | GG | GG |  |
| chrC2~94071832 | T | C | CC | CT | CC | CT | CT | CC | CC |  |
| chrD3~18276526 | T | C | TT | CT | TT | CT | CT | TT | TT |  |
| chrC1~77481695 | C | T | CT | CC | CT | CT | CC | CC | CC |  |
| chrB1~94269990 | G | A | AG | AG | AA | AA | AA | GG | AA |  |
| chrB2~168562721 | C | G | CC | GG | CC | CG | CC | CC | CG |  |
| chrD4~59018447 | C | T | CC | TT | CC | CT | CC | CT | CC |  |
| chrF2~974749 | A | G | GG | AG | GG | AG | AA | GG | GG |  |
| chrUn11~96020 | G | A | GG | AG | GG | AA | AG | GG | GG |  |
| chrUn30~724772 | A | C | CC | AC | CC | CC | CC | AC | AA |  |
| chrB3~116725376 | T | C | CC | TT | CT | TT | CT | TT | TT |  |
| chrUn7~355126 | T | C | CT | CC | CC | TT | CC | CT | CC |  |
| chrB2~114075694 | A | G | AA | NN | AA | AG | AG | AA | GG |  |
| chrA3~139311944 | T | C | CC | CT | CC | CC | CC | TT | TT |  |
| chrB3~126951606 | C | T | TT | CC | CC | CT | CC | CC | TT |  |
| chrE1~125778559 | T | C | CC | TT | TT | CT | TT | CC | TT |  |
| chrD4~31690190 | T | C | CC | CC | CC | CC | TT | TT | TT |  |
| chrE1~58582388 | G | A | AA | AA | GG | GG | GG | GG | AA |  |
| chrB3~57417229 | T | A | AA | AT | TT | AA | TT | NN | NN |  |
| chrA3~147325137 | C | T | CT | CC | CT | CC | CC | CT | CT |  |
| chrF1~87653985 | G | A | GG | AG | AG | AG | GG | AG | NN |  |
| chrA1~145764245 | G | C | CG | CC | CC | CG | CC | GG | CG |  |
| chrA3~32523886 | T | C | CC | CT | CT | CT | CC | CC | TT |  |
| chrC1~56249999 | G | A | GG | AG | AG | GG | AA | GG | AG |  |
| chrD4~9816190 | G | A | AG | GG | GG | GG | AG | AG | AA |  |
| chrA2~11653359 | T | G | TT | GG | GT | GG | GG | GT | TT |  |
| chrA2~199242623 | G | A | AA | AA | GG | GG | AG | AA | AG |  |
| chrA3~135490615 | T | G | TT | GG | GT | GT | GG | GG | TT |  |
| chrC2~119374055 | G | A | GG | GG | AA | AA | GG | AG | AG |  |
| chrUn34~4722819 | G | A | AA | GG | AG | GG | AA | GG | AG |  |
| chrA2~126524301 | G | A | AA | GG | GG | AA | AG | AG | GG |  |
| chrA2~41349421 | T | C | TT | TT | CC | CC | TT | CC | CT |  |
| chrB3~149950264 | A | C | CC | AA | AA | AA | CC | CC | AC |  |
| chrUn5~7719572 | A | T | AA | AT | AT | AT | AA | AT | AT |  |
| chrA3~139186800 | T | G | TT | TT | GG | GT | GT | GT | GT |  |
| chrA3~25758760 | G | A | AG | AA | AG | AG | GG | AG | AA |  |
| chrB4~158462078 | A | G | AG | GG | AG | AG | AA | AG | GG |  |
| chrC2~119593341 | A | G | AG | AG | AA | AG | GG | GG | AG |  |
| chrD2~7924743 | A | G | AG | AA | AG | AG | GG | AG | GG |  |
| chrD4~66422082 | A | G | GG | AG | AG | AG | AA | AA | AG |  |
| chrUn9~6160568 | G | A | AG | AG | AA | GG | AA | AG | AG |  |
| chrA1~145174233 | T | C | CC | CC | CT | CT | TT | CT | CT |  |
| chrA3~34024152 | A | G | GG | AG | AA | AG | AG | AA | AG |  |
| chrB2~44092667 | G | A | AG | AA | AA | AG | AG | GG | AG |  |
| chrE1~46436515 | C | T | CC | CC | CT | CT | TT | CT | TT |  |
| chrB1~30644593 | C | G | CC | CC | CC | CC | CC | CC | CC |  |
| chrD4~17710016 | C | T | NN | CC | CC | CC | CC | CC | CC |  |
