# Supplementary material for: Light whole genome sequence for SNP discovery across domestic cat breeds
Source: BMC Genomics. 2010 Jun 24;11:406. doi: 10.1186/1471-2164-11-406 (PMC2996934; doi:10.1186/1471-2164-11-406)
Supplement: Supplementary file 6 [file 1471-2164-11-406-S6.DOC]

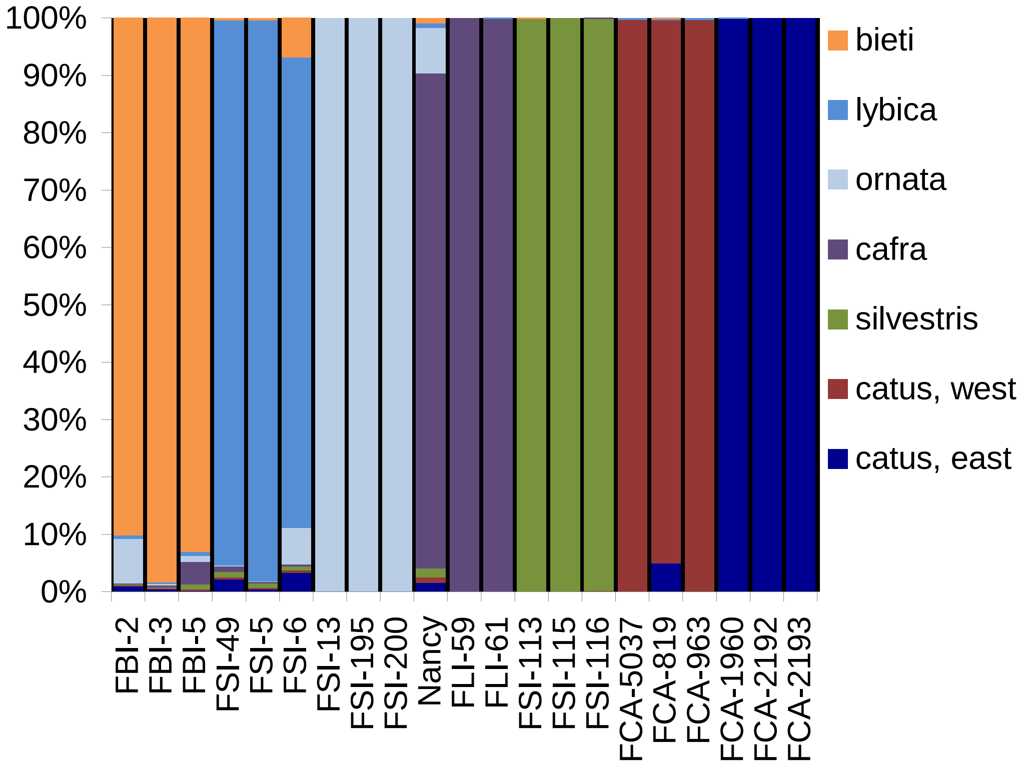

Figure S1. Results of STRUCTURE analysis showing Nancy and representative known-origin wildcat individuals.
